# Systematics and diversification of the Ichthyomyini (Cricetidae, Sigmodontinae) revisited: evidence from molecular, morphological, and combined approaches

**DOI:** 10.7717/peerj.14319

**Published:** 2023-01-13

**Authors:** Jorge Salazar-Bravo, Nicolás Tinoco, Horacio Zeballos, Jorge Brito, Daniela Arenas-Viveros, David Marín-C, José Daniel Ramírez-Fernández, Alexandre R. Percequillo, Thomas E. Lee, Jr., Sergio Solari, Javier Colmenares-Pinzon, Carlos Nivelo, Bernal Rodríguez Herrera, William Merino, Cesar E. Medina, Oscar Murillo-García, Ulyses F.J. Pardiñas

**Affiliations:** 1 Department of Biological Sciences, Texas Tech University, Lubbock, Texas, United States; 2 Instituto de Ecologia, Universidad Mayor de San Andrés, La Paz, Bolivia; 3 Instituto Nacional de Biodiversidad, Quito, Ecuador; 4 Museo de Zoología Escuela de Ciencias Biológicas, Pontificia Universidad Católica del Ecuador, Quito, Ecuador; 5 Universidad Católica de Santa María, Arequipa, Peru; 6 Colección Teriológica, Universidad de Antioquia, Medellin, Colombia; 7 Skuë’ Conservation, Costa Rica Wildlife Foundation, San José, Costa Rica; 8 Escola Superior de Agricultura “Luiz de Queiroz”, Departamento de Ciências Biológicas, Universidade de São Paulo, Piracicaba, São Paulo, Brazil; 9 Department of Biology, Abilene Christian University, Abilene, Texas, United States; 10 Instituto de Biología, Universidad de Antioquia, Medellin, Antioquia, Colombia; 11 Grupo de Estudios en Biodiversidad, Escuela de Biología, Universidad Industrial de Santander, Bucaramanga, Santander, Colombia; 12 Museo de Zoologia, Escuela de Biología, Universidad del Azuay, Cuenca, Ecuador; 13 Instituto de Diversidad y Evolución Austral, Consejo Nacional de Investigaciones Científicas y Técnicas, Puerto Madryn, Chubut, Argentina; 14 Escuela de Biología, Universidad de Costa Rica, San José, San José, Costa Rica; 15 Escuela de Biología, Universidad de El Salvador, San Salvador, San Salvador, El Salvador; 16 Museo de Historia Natural, Universidad Nacional de San Agustin, Arequipa, Arequipa, Peru; 17 Departamento de Biología, Universidad del Valle, Cali, Valle del Cauca, Colombia

**Keywords:** Amazon, Andean, *Chibchanomys orcesi*, *Daptomys*, Ichthyomyini, Neotropics, Sigmodontalia, Subtribes, Water rats

## Abstract

Ichthyomyini, a morphologically distinctive group of Neotropical cricetid rodents, lacks an integrative study of its systematics and biogeography. Since this tribe is a crucial element of the Sigmodontinae, the most speciose subfamily of the Cricetidae, we conducted a study that includes most of its recognized diversity (five genera and 19 species distributed from southern Mexico to northern Bolivia). For this report we analyzed a combined matrix composed of four molecular markers (*RBP3*, *GHR*, *RAG1*, *Cytb*) and 56 morphological traits, the latter including 15 external, 14 cranial, 19 dental, five soft-anatomical and three postcranial features. A variety of results were obtained, some of which are inconsistent with the currently accepted classification and understanding of the tribe. Ichthyomyini is retrieved as monophyletic, and it is divided into two main clades that are here recognized as subtribes: one to contain the genus *Anotomys* and the other composed by the remaining genera. *Neusticomys* (as currently recognized) was found to consist of two well supported clades, one of which corresponds to the original concept of *Daptomys*. Accordingly, we propose the resurrection of the latter as a valid genus to include several species from low to middle elevations and restrict *Neusticomys* to several highland forms. Numerous other revisions are necessary to reconcile the alpha taxonomy of ichthyomyines with our phylogenetic results, including placement of the Cajas Plateau water rat (formerly *Chibchanomys orcesi*) in the genus *Neusticomys* (*sensu stricto*), and the recognition of at least two new species (one in *Neusticomys*, one in *Daptomys*). Additional work is necessary to confirm other unanticipated results, such as the non-monophyletic nature of *Rheomys* and the presence of a possible new genus and species from Peru. Our results also suggest that ichthyomyines are one of the main Andean radiations of sigmodontine cricetids, with an evolutionary history dating to the Late Miocene and subsequent cladogenesis during the Pleistocene.

## Introduction

The rodent subfamily Sigmodontinae comprises the most diverse radiation among Neotropical mammals (see a synthesis in Patton, Pardiñas & D’Elía, 2015 and Pardiñas et al., 2017a). Broadly distributed, these cricetids occupy almost all available habitats in the extensive geography of the Neotropics and portions of the Nearctic, stemming from a diversification process that resulted in 96 recent genera—6 of which have gone extinct in historical times—and about 460 species (Fabre et al., 2012; Patton, Pardiñas & D’Elía, 2015; Pardiñas et al., 2017a; Steppan & Schenk, 2017).

The last three decades of systematic work have produced several hypotheses on sigmodontine relationships (*e.g*., Smith & Patton, 1999; Weksler, 2003; D’Elía, 2003; Jansa & Weksler, 2004; Leite et al., 2014; Salazar-Bravo, Pardiñas & D’Elía, 2013; Parada et al., 2013; Pardiñas, Teta & Salazar-Bravo, 2015; Salazar-Bravo et al., 2016; Steppan & Schenk, 2017; Percequillo et al., 2021; Parada, Hanson & D’Elía, 2021; Pardiñas et al., 2022). These studies agree that the subfamily is composed of two major living groups: the Oryzomyalia *sensu* Steppan, Adkins & Anderson (2004) and the Sigmodontalia *sensu* Leite et al. (2014). Oryzomyalia is composed of predominantly South American forms which, in a relatively short time span, diversified into no less than 11 tribal-level lineages plus a few genera with controversial phyletic positions (*i.e*., *incertae sedis*) and poorly resolved intertribal relationships (see a synthesis in Cazzaniga, Cañón & Pardiñas, 2019; Ronez et al., 2021).

Sigmodontalia is composed of two morphologically and ecologically contrasting lineages: Sigmodontini and Ichthyomyini. The Sigmodontini, restricted to the single genus *Sigmodon* Say & Ord, 1825 (varyingly including or not the fossil form *Prosigmodon*; Barbière, Ortiz & Pardiñas, 2016) ranges from throughout the southwestern and southeastern United States and Mexico, through Central America, to northern South America. Species within *Sigmodon* are markedly pastoral, with hypsodont, laminated molars, and occur in grassy habitats and other open landscapes (Voss, 1992). The genus is present in the fossil record of North America, from the late Pliocene (Early Blancan) and was moderately diverse during the Pleistocene as several extinct species are known (Martin, 1979; Peláez-Campomanes & Martin, 2005).

The other member of the Sigmodontalia is the tribe Ichthyomyini, a group of rodents with strong adaptations to carnivory and semiaquatic life. Ichthyomyine rodents are known from southern Mexico to northern Bolivia, with several species of mostly Andean occurrence. The tribe has the distinction of being the first sigmodontine group to receive a modern systematic revision and to be demonstrably monophyletic by the identification of robust morphological synapomorphies (Voss, 1988). Voss (1988) recognized five genera and 14 species including: *Ichthyomys* Thomas, 1893 (four species), *Anotomys* Thomas, 1906a (1 sp.), *Rheomys* Thomas, 1906b (4 spp.), *Neusticomys* Anthony, 1921 (4 spp., with *Daptomys* Anthony, 1929, as a junior synonym), and *Chibchanomys* Voss, 1988 (1 sp.). Additionally, this author recognized two species groups within *Rheomys* and arranged eight nominal taxa in *Ichthyomys* into four biological species: *I. hydrobates* (Winge, 1891) (with subspecies *hydrobates*, *nicefori* Thomas, 1924a, and *soderstromi* de Winton, 1896), *I. pittieri* Handley & Mondolfi, 1963, *I. stolzmanni* Thomas, 1893 (with subspecies *orientalis* Anthony, 1923 and *stolzmanni*), and *I*. *tweedii* Anthony, 1921 (including *I*. *caurinus* Thomas, 1924b as a junior synonym). Subsequent alpha taxonomic research has augmented the number of ichthyomyine species currently recognized as valid to 19, including *Neusticomys mussoi* Ochoa & Soriano, 1991; *Chibchanomys orcesi* Jenkins & Barnett, 1997; *Neusticomys ferreirai* Percequillo, Carmignotto & Silva, 2005; *Neusticomys vossi* Hanson et al., 2015; and *Ichthyomys pinei* Férnandez de Córdova et al., 2020. Additionally, a new subspecies was recently described from the lowlands of Peru (*Neusticomys peruviensis musseri* Pacheco et al., 2020).

Among other unresolved issues in ichthyomyine systematics is the generic status of *Daptomys*. The genus *Daptomys*, with *D*. *venezuelae* as type species, was described by Anthony (1929) to include forms that “…appear(ed) to be intermediate between the highly specialized *Ichthyomys* and *Anotomys* on the one hand, and the more generalized *Rheomys* and *Neusticomys* on the other” (Anthony, 1929:4). Voss (1988) compared characters that distinguished *D*. *venezuelae* from *Neusticomys monticolus* (type species of *Neusticomys*) and concluded that the observed phenetic differences were not “…equivalent to the phenotypic differences that separated other ichthyomyine genera…” (Voss, 1988: 343) and proposed to treat *Daptomys* as a junior synonym of *Neusticomys* (Voss, 2015; Brito & Pardiñas, 2017).

Recent fieldwork has resulted in major range extensions for several species of ichthyomyines and provided new information concerning their biology and natural history. Consequently, we now know that some species formerly considered to be geographically restricted are much more broadly distributed and occupy a wider range of habitats than previously thought, for example, *Neusticomys mussoi* (see Rodriguez-Posada, 2014), *Ichthyomys stolzmanni* (see Brito, Tenecota & Pozo-Zamora, 2016), *Anotomys leander* (see Marín-C & Sanchez-Giraldo, 2017), *Neusticomys peruviensis* (see Medina et al., 2015; Gonzales, Arce-Merma & Zeballos, 2017; Pacheco et al., 2020), and *Ichthyomys tweedii* (see Ramírez-Fernández, Durán & Fernández-Vargas, 2020). Importantly, these efforts have also made dozens of fresh specimens available for morphological and molecular analyses (*e.g*., *Neusticomys oyapocki*; Catzeflis et al., 2017), opening a window of opportunity to advance hypotheses on the systematics and phylogenetics among ichthyomyine genera and species.

These new specimens, and a strong multi-institutional collaborative effort allowed us to address several salient issues concerning ichthyomyine systematics. First, we aim to evaluate the monophyly, the composition, and the structure of the Ichthyomyini employing a multi-loci molecular dataset. For this purpose, we included representatives of all recognized genera and other taxa for which no nuclear data were previously assessed or for which no molecular data were ever generated. Second, we used a matrix of previously compiled and newly generated morphological data to incorporate in our analyses a handful of ichthyomyine taxa for which no molecular data were available. On the assumption that combining morphological and molecular matrices results in more accurate phylogenies, we present a total-evidence phylogenetic analysis for the tribe. Third, we explore hypotheses about the tempo of tribal diversification in the context of late Neogene/early Quaternary geological and ecological events that offer new insights into major questions regarding the evolutionary history of this fascinating group of rodents.

## MATERIALS AND METHODS

### Studied specimens

This contribution is based on museum specimens, as well as animals collected over the last 10 years (Table S1). Collecting permits that allowed the study include, in alphabetic order: Bolivia (Colección Boliviana de Fauna, Resolución 14 August 1992), Brazil (Instituto Brasileiro do Meio Ambiente e dos Recursos Naturais Renováveis (IBAMA), Pacajá: 519/2014, Proc. 02001.001182/2014-65, Instituto Chico Mendes de Conservação da Biodiversidade [ICMBio], PN Pacaás Novos: 36753-3); Colombia (Autoridad Nacional de Licencias Ambientales del Ministerio de Ambiente y Desarrollo Sostenible de la República de Colombia: Resolución 1070 [28 August 2015], Resolución 01711 [30 December 2016], Resolución 0200 [13 April 2015]); Costa Rica (R-SINAC-PNI-SE-002-2018); Ecuador (Ministerio del Ambiente, Resolución 002-IC-FAUFLO-DRFN-PMA, Resolución 010-IC-FAU/FLO-DRFN-P/MA, Resolución 012-IC-FAU-DNBAPVS/MA, Resolución MAE-DPAC-2014-0280, Resolución 2-12 IC-FAU-FLO-DPACMA), El Salvador (Resolución MARN-DEV-GVS-AIMA-087-2017), Peru (Dirección General de Flora y Fauna Silvestre [DGFFS], Servicio Nacional de Áreas Naturales Protegidas, Zona Reservada Sierra del Divisor and Santuario Histórico de Machupicchu: RD 503-2011-AG-DGFFS, RJ 003-2013-SERNANP-ZRSD, RJSHM-No. 054-2012-SERNANP-JEF).

### Molecular data

**Taxonomic** **sampling**: We included sequence data from 59 individuals belonging to 15 of the 19 currently recognized species in the Ichthyomyini; the only nominal ingroup taxa not represented in our molecular dataset include: *Ichthyomys pittieri*, *Ichthyomys pinei*, *Neusticomys venezuelae* (Anthony, 1929), and *Rheomys underwoodi* Thomas, 1906b. Sequences generated as part of this study were supplemented with the few sequences available from previous studies and/or GenBank to maximize coverage of ingroup and outgroup taxa. In the outgroup, we included representatives of most extant genera in the Sigmodontinae (except for *Mindomys* and *Gyldenstolpia*) plus several representatives of other cricetid subfamilies.

Molecular markers were selected to complement coverage from GenBank and include one mitochondrial (cytochrome-b, *Cytb*), and three nuclear markers: exon 10 of the growth hormone receptor (*GHR*), exon 1 of interphotoreceptor retinoid binding protein (*RBP3*, aka *IRBP*), and the single exon of the recombination activating gene 1 (*RAG1*). In addition to the high-signal-to noise ratio for phylogenetic inference in these markers, there is evidence that they show minimal signal conflict, allowing even limited genetic sampling to yield trees very close to those from larger datasets (Steppan & Schenk, 2017).

**Figure 1:**
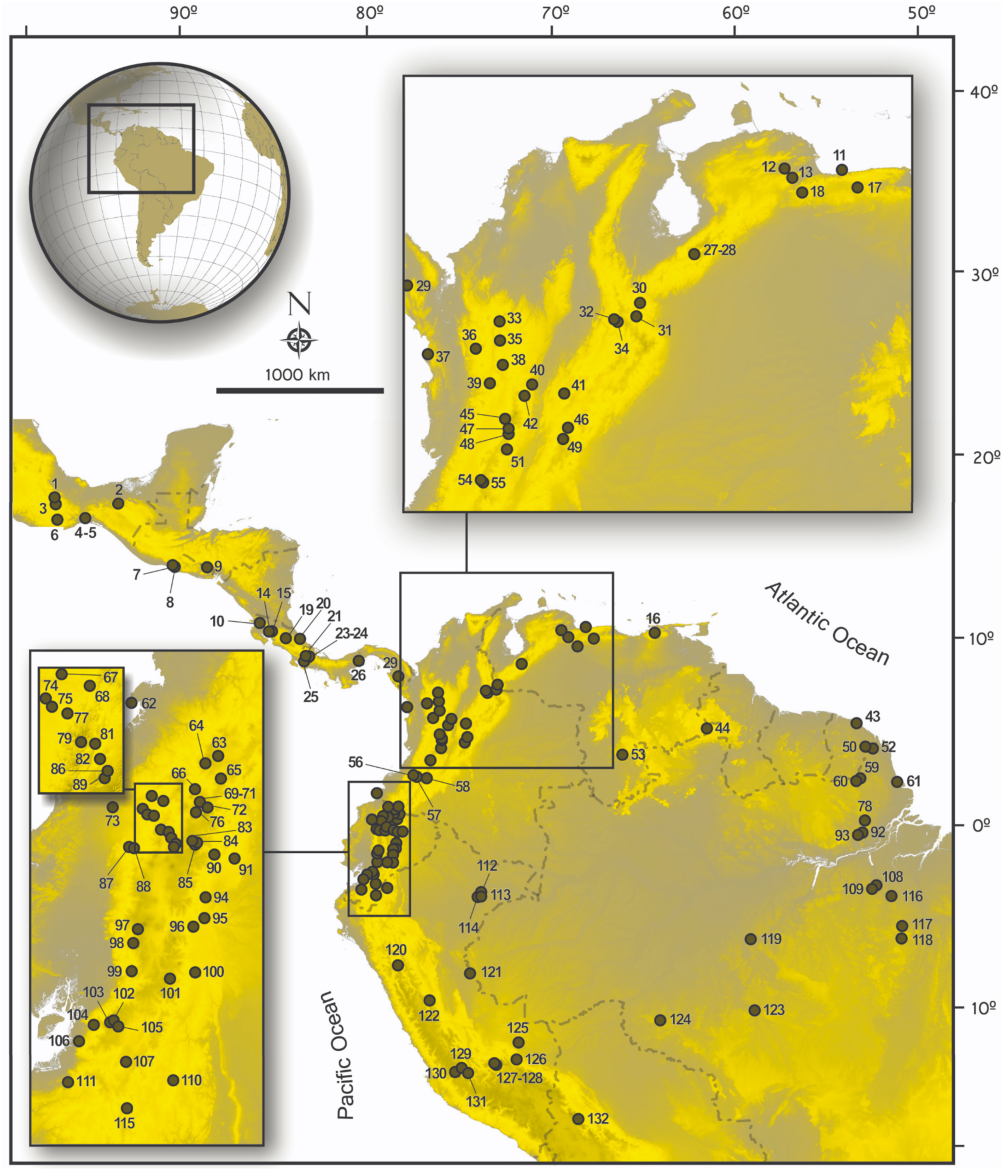
Map of collecting localities of ichthyomyines specimens used in the present study. Map of localities of specimens of the Ichthyomyini included in genetic and morphological analyses in the present study. Numbered localities correspond to those of Table S1.

For most specimens, DNA was isolated from tissue that had been frozen or preserved in ethanol in the field using the Qiagen DNeasy Tissue Extraction Kit (Qiagen, Inc., Valencia, CA, USA) and following the manufacturer’s protocol. In some cases, DNA was isolated from toe clips or skin samples following protocols presented in Giarla, Voss & Jansa (2010). Extracted DNA was used as a template in polymerase chain reaction (PCR) amplifications. We amplified and sequenced *Cytb* with primers and methods described elsewhere (Pardiñas et al., 2014; Salazar-Bravo et al., 2016). Amplification of *RBP3* was performed following the methods of Jansa & Voss (2000), *GHR* following Rowe & Honeycutt (2002), and *RAG1* following Steppan, Adkins & Anderson (2004), or Steppan et al. (2007). Cycling conditions were set as follows: 94 °C for 3 min, followed by either 35 or 40 cycles of 94 °C for 45 s, 50 °C (*Cytb*), 56 °C (*GHR*) or 58 °C (*RBP3*) for 45 s, 72 °C for 90 s, and final extension at 72 °C for 10 min. The mtDNA C*ytb* region was amplified with the primers MVZ05 and MVZ14. The *RBP3* region was amplified with the primers IRBP111A1 and IRBP1531 (see Jansa & Voss, 2000 and Stanhope et al., 1992, respectively for primer sequences). The *GHR* region was amplified with the primers *GHR*50F and *GHR*end, or *GHR*5 and *GHR*6. For *RAG1*, cycling conditions were set as follows: 94 °C for 3 min, followed by touchdown cycles of 94 °C for 45 s, 61 °C for 45 s, 72 °C for 30 s (5 cycles), after which annealing temperatures were 59 °C for 45 s (5 cycles), 57 °C for 45 s (5 cycles), 55 °C for 45 s (5 cycles), and 53 °C for 45 s (15 cycles), and final extension at 72 °C for 5 min. A portion (ca. 1,300 bp) of the 5′–end region of the *RAG1* was amplified with primers S70 and S142 (Steppan et al., 2007). Amplified products were sequenced in both directions either at Macrogen Korea (samples processed in Ecuador and Peru), the Servicio de Secuenciación y Análisis Molecular (SSiGMol), Universidad Nacional de Colombia (for samples processed in Colombia), and Macrogen USA for the remainder of the samples. Sequences were visualized, reconciled and translated into proteins to proof for stop codons using SeqMan (DNASTAR, 2003). The map in Fig. 1, shows the geographic provenance of specimens used in this study and the Table S1 presents a list of taxa, museum codes for specimens examined, and access codes for gene segments included in this study.

### Phylogenetic analyses of DNA sequences

Since the markers selected were protein-coding genes, alignment was non-problematic and was conducted with Muscle v3.8 (Edgar, 2004) in Mesquite v3.61 (Maddison & Maddison, 2019) using the default values for all alignment parameters. Ambiguous regions in the alignments of *RAG1* and *GHR* were cleaned with trimAl version 1.4 (Capella-Gutierrez, Silla-Martinez & Gabaldon, 2009).

We combined the mitochondrial and nuclear exon sequences into two concatenated super matrices. In the extended supermatrix, we included all ichthyomyine sequences, plus outgroup sequences (133 terminals, 4,284 sites, with 31.1% of missing character states); this matrix was used to evaluate general patterns of relationships among taxa and, importantly, to assess potentially overlooked diversity in the tribe. The 2^nd^ matrix (the ingroup supermatrix) incorporated only one representative per ingroup species and was employed in combination with morphological data to test within-tribe hypotheses of relationships. This matrix had 24 terminals, 56 morphological and 4,652 DNA molecular characters, with 41% of missing character states.

Aligned, curated sequences were subjected to phylogenetic analyses using maximum likelihood (ML, Felsenstein, 1981) and Bayesian inference (BI, Ronquist et al., 2012). The data sets were analyzed using partitioning schemes and substitution models identified by PartitionFinder (Lanfear et al., 2012). BI analyses were conducted with MrBayes on the CIPRES Science gateway (Miller, Pfeifer & Schwartz, 2010) and included gene specific unlinked models in the analyses of combined data sets, assuming uniform interval priors for all parameters except base composition. The model of molecular evolution used was SYM+I+G for all genes and all codon positions, except for 3^rd^ base position in *Cytb* gene for which GTR+I+G was selected. Analyses consisting of four independent runs, each with three heated and one cold Markov chains, were allowed to proceed for 10 million generation and sampled every 1,000 generations; these analyses were repeated five times. The first 25% of trees were discarded as “burn-in” and the remaining trees were used to compute a 50% majority rule consensus tree and to obtain posterior probability (PP) estimates for each clade. Branches with posterior probability [PP] ≥0.95 were considered well supported. All analyses were checked for convergence by plotting the log-likelihood values against generation time for each run, using Tracer 1.7.1 (Rambaut et al., 2018). After each of the runs, we confirmed that all parameters had effective sample sizes greater than 200. The maximum-likelihood trees were calculated using IQ-TREE 1.6.12 (Trifinopoulos et al., 2016) using the models selected by PartitionFinder and ultrafast nodal bootstrap values (Hoang et al., 2018) for the analyses. Because Bayesian PP values tend to be less conservative estimates of node reliability than nonparametric bootstrap values [BS], inclusion of both support values on our trees represents the upper and lower bounds, respectively, of node reliability (Douady et al., 2003).

### Combined molecular and morphology (total evidence) analyses

We followed the morphological character descriptions of the integument, skull, postcranial skeleton, and soft anatomy provided by Voss (1988). Using these descriptions, along with the study of several new specimens, we modified the definition of some characters and states, and added new traits—especially from the molar dentition—which resulted in a new matrix of 56 discrete morphological characters. The morphological data matrix included one character from the general external bauplan, 14 characters of the integument, 14 cranial characters, 19 dental characters, five soft-anatomy characters, and three postcranial skeletal characters (Appendix 1). Characters were scored for all currently recognized species and individuals from two presumed new ichthyomyine species, resulting in a matrix with 19% polymorphic characters for one or more of the 24 taxa represented. In these analyses we also scored LSU 14406, currently identified as *Chibchanomys orcesi* by Voss (2015) to assess the phylogenetic placement of this specimen. Morphological data was only available for ingroup taxa.

**Appendix 1:** Morphological characters used in the phylogenetic analyses

Unless otherwise noted, all characters describe conditions for adult individuals. For most of the listed characters, a brief description is provided and, if necessary, illustrations are also included.

We concatenated our matrix of discrete morphological and molecular data for members of the Ichthyomyini on PAUP*. This combined matrix included a total of 4,708 characters with 56 morphological characters and 4,652 DNA characters. In order to incorporate taxa for whom we lacked molecular data (*i.e*., *Ichthyomys pittieri*, *Ichthyomys pinei*, *Neusticomys venezuelae*, *Rheomys underwoodi*, and LSU 14406) into a comprehensive analysis of the relationships among members of the tribe, molecular characters were scored as missing (“?”).

We considered three partitions for this data set, two as described above for molecular characters and one partition for morphological data following a Mkv+G model of discrete character evolution (Lewis, 2001). We conducted three BI analyses on MrBayes version 3.2.7a (Ronquist et al., 2012) using this set of partitions, and running four Markov chains for 20 million generations, sampling trees every 10,000 generations. We assessed stationarity of the chains on Tracer v1.6 (Rambaut et al., 2018); trees generated before stationarity were discarded as “burn-in” (25% of the trees in our analyses). The three Bayesian analyses gave the same tree topology.

### Molecular dating

Molecular dating analyses were performed with the fossilized birth-date (FBD) model (Heath, Huelsenbeck & Stadler, 2014; Zhang et al., 2016) on a combined matrix of morphology and molecular data reduced to include only one individual per species of the Ichthyomyini, and representative outgroup taxa. Based on the analyses of the concatenated dataset LSU 14406 was considered conspecific with “*Chibchanomys*” n. sp. from Peru, and not included in the dating analysis. The FBD model takes advantage of the Bayesian approach to incorporate information concerning speciation, extinction, and preservation/recovery sampling processes, with the aim of uniting extinct and extant species with a single evolutionary model (Gavryushkina et al., 2014). Fossil calibrations used in this study are listed in Table 1.

**Table 1:** Calibration points, based on fossil cricetid occurrences, used for dating analyses.

#	Taxon	Age in Ma	Remarks
1	*Reithrodon*	3.79 ± 0.5	Oldest confident record for the genus *Reithrodon*; basal levels of the Chapadmalal Fm (Buenos Aires, Argentina); absolute age from dated impactites (Prevosti et al., 2021).
2	*Graomys*	3.79 ± 0.5	Oldest record for the genus *Graomys* based on *Graomys dorae* Reig, 1978; basal levels of the Chapadmalal Fm (Buenos Aires, Argentina).
3	*Scapteromys aquaticus* /*tumidus*	1.86 ± 0.09	Oldest record for a morphotype indistinguishable from *Scapteromys aquaticus* or *Scapteromys tumidus*; based on a specimen collected in Necochea (Buenos Aires, Argentina); age according to paleomagnetic profile (Prevosti et al., 2021)
4	*Kunsia/Scapteromys* (split)	2.75 ± 0.25	Based on *Scapteromys hershkovitzi* Reig, 1994 (see Pardiñas, 2013); absolute age base on fission track analysis conducted in Olavarría (M. de los Reyes, 2020, personal communication).
5	*Akodon montensis*	0.88 ± 0.105	Remains attributed to this species collected near Mar del Plata (Buenos Aires, Argentina; see Reig, 1987); age according to paleomagnetic data (Brunhes-Matuyama limit).
6	*Necromys*	2.4 ± 0.2	San Andrés Fm; recorded as *Bolomys* sp. in Reig (1994); age according to paleomagnetic data.
7	*Kraglievichimys formosus* (Phyllotini)	4.40 ± 0.05	Based on remains collected in Irene Fm, Quequén Salado river (Buenos Aires, Argentina; see Pardiñas et al., 2017b; Barbière, Ortiz & Pardiñas, 2019) absolute age from dated impactites (Prevosti et al., 2021).
8	*Sigmodon curtisi*	2.00	Oldest record of genus *Sigmodon stricto sensu* (see Peláez-Campomanes & Martin, 2005).
9	*Akodon johannis*	1.40	Treated as the oldest record of genus *Thaptomys* (see Reig, 1987).
10	*Akodon lorenzini*	2.80	Treated as the oldest record of genus *Akodon* (see Reig, 1987).
11	*Neotoma sawrockensis*	4.70	Applied to the split between *Neotoma* and *Hodomys* (Martin & Zakrzewski, 2019)

We followed the recommended settings in the BEAST v2 tutorial (Heath, 2015) with the following exceptions: For the tree, origin was set at 18My based on the time of split between Cricetidae and Muridae as shown in Steppan & Schenk (2017), diversification rate = 0.3, turnover = 0.2 and sampling proportion = 0.3 based on the number of species from the family Cricetidae included in the analysis. We assumed an exponential prior for the uncorrelated lognormal relaxed-clock rate, with mean = 1 and standard deviation = 1, as these parameters broadly encompass the commonly cited estimate of 10^−9^ substitutions/site/million years for mammalian nuclear gene evolution (*e.g*., Kumar & Subramanian, 2002) as well as the average mammalian Cytb neutral substitution which is nearly 60 times higher than the nuclear one (Nabholz, Glémin & Galtier, 2008). The same substitution models implemented in the Bayesian analysis were applied in BEAST for 60 million generations, sampling every 1,200 generations. Using the CIPRES Science Gateway portal (Miller, Pfeifer & Schwartz, 2010), several runs were completed and after evaluating their performance using Tracer (*i.e*., ESS > 200), trees from the best two were combined and annotated using LogCombiner and TreeAnnotator, respectively.

### Estimating diversification parameters

We constructed a diversity-through-time (DTT) plot based on the time-scaled maximum-clade-credibility tree derived from the BEAST analysis and evaluated the fit of a birth-death model to these branching times using the cladogenetic diversification rate shift model (ClaDS, Maliet & Morlon, 2022) as implemented in the PANDA package (https://github.com/hmorlon/PANDA.jl) in Julia (Bezanson et al., 2017). We incorporated sampling probabilities for clades in the phylogeny by considering the estimated species diversity of each containing clade based on recent and ongoing revisionary work (for example, in *Rheomys*, *Ichthyomys*, and *Daptomys*).

Convergence of the MCMC, was monitored by running three chains simultaneously; after discarding the first quarter of the iterations runs were stopped using the Gelman statistic (Gelman et al., 2014) for the four hyperparameters and the lineage-specific rates for each branch in the reconstructed phylogeny. ClaDS stops the algorithm when the Gelman statistics reaches values below 1.05 for all the parameters. The posterior density of the model parameters was assessed with maximum *a posteriori* (MAP) estimates, which were then used as point estimates for each of the parameters. To do so, we apply the function *density* from the R-package stats (R Core Team, 2021) to our MCMC chain and recorded the parameter values for which the maximum density was reached.

### Biogeographical analyses

Biogeographical analyses were performed with the programs *rase* (Quintero et al., 2015) and BioGeoBEARS (Matzke, 2014) as implemented in RASP v4.2 (Yu, Blair & He, 2020). Because these methods use different approximations, concordance in their results is taken as evidence of biogeographic history. To perform the biogeographical analyses, we used the resulting consensus tree from the BEAST analysis.

The *rase* method uses the entire known species distribution to infer the geographical location of putative ancestors in a Bayesian framework. The geographical distributions of the species of interest were extracted from Patton, Pardiñas & D’Elía (2015) and Pardiñas et al. (2017a); in the cases of presumed new species, we used the known sampled localities for these taxa. No assumptions were made on the correlation between dispersal rates and either longitude and/or latitude. We ran *rase* for 10,000 iterations, and after discarding the first 1,000 as “burn-in” we logged every 10^th^ iteration and obtained the posterior distributions of ancestral nodes and migration rates as longitudinal (σ2x) and latitudinal (σ2y) degrees^2^ over Mya. We plotted the trace to evaluate the MCMC results, estimated the mean and posterior densities for each of the estimated parameters and confirmed that the algorithm converged to the posterior distribution with the R package *coda* (Plummer et al., 2006). We applied evenly spaced time slices (approximately 1.5 Mya intervals) to infer the location of the ancestral nodes. The *rase* and *coda* packages were run in R 4.0.4 using the interface R-Studio.

In addition, putative ancestral geographic distributions in the Ichthyomyini were inferred with the BioGeoBEARS package implemented in RASP v4.2 (Yu, Blair & He, 2020). This ML-based approach calculates values for biogeographical state changes along branches of a phylogeny to estimate ancestral areas of occupation. First, using likelihood ratio tests we evaluated the fit of our data to six basic distinct biogeographic models: DEC, DEC+J, DIVALIKE, DIVALIKE+J, BAYAREALIKE, and BAYAREALIKE+J and used the Akaike Information Criterion (AICc) and Akaike weights (AICc_wt) to select the best model. The founder-event speciation parameter (+J) was evaluated despite the cautionary advice of Ree & SanMartín (2018) and attending to recommendations by Matzke (2021). The model selected was the dispersal–extinction–cladogenesis model (DEC; Ree & Smith, 2008). As suggested by these authors, analyses were run excluding ranges that represent noncontiguous or widely disjunct distributions; in addition, only most-likely scenarios were mapped.

A total of six areas were defined for the ancestral range estimation broadly corresponding to bioregions A, B, I, J and K of Maestri, Upham & Patterson (2019). On account of previous zoogeographic interpretations of ichthyomyine distributions, we divided their bioregion “I” in two units to emphasize the known effect of the Atrato River on the distribution patterns of icthyomyine rodents (Voss, 1988). Maestri, Upham & Patterson (2019) sigmodontine bioregions were based on species ranges collected from Patton, Pardiñas & D’Elía (2015). Despite the acknowledged shortcomings of the so-defined bioregions (*e.g*., overestimation of geographic ranges for many species), they are likely to better reflect the particular ecological and evolutionary history of sigmodontine rodents over the Neogene than what would be achieved by using more general biogeographic categorizations such as the ecoregions of Morrone (2014), for example.

### New zoological taxonomic names

The electronic version of this article in Portable Document Format (PDF) will represent a published work according to the International Commission on Zoological Nomenclature (ICZN), and hence the new names contained in the electronic version are effectively published under that Code from the electronic edition alone. This published work and the nomenclatural acts it contains have been registered in ZooBank, the online registration system for the ICZN. The ZooBank LSIDs (Life Science Identifiers) can be resolved, and the associated information viewed through any standard web browser by appending the LSID to the prefix http://zoobank.org/. The LSID for this publication is: urn:lsid:zoobank.org:pub:92FE6565-5569-40EF-89B9-211DA7A16508. The online version of this work is archived and available from the following digital repositories: PeerJ, PubMed Central and CLOCKSS.

## Results

We generated a total of 101 new DNA sequences, all of which are now deposited in GenBank (File S1). For the first time, exemplar species from all currently recognized ichthyomyine genera are represented by at least four molecular markers. Sequence alignments of *Cytb* and *RBP3* did not require gaps (except in a handful of genera for the latter), but those of *GHR* and *RAG1* required insertion of multiple base-pairs. Amino-acid translations of these genes did not contain any premature stop codons. Potentially anomalous DNA assemblies and/or contaminations were assessed by building phylogenetic trees for individual genes; as expected, topological conflicts for gene trees based on *Cytb vs*. nuclear exons were relatively common, although none was well-supported (1 > PP > 0.95). Subsequent analyses relied on concatenation of the mitochondrial and nuclear exon super-matrices, on the premise that the much faster-evolving mitochondrial *Cytb* helped on reconstructing relationships within/among species and within genera, with slower-evolving nuclear exons providing information on the deeper nodes (*e.g*., among genera) of ichthyomyine and outgroup taxa.

### Molecular phylogenetics of the extended supermatrix

**Figure 2:**
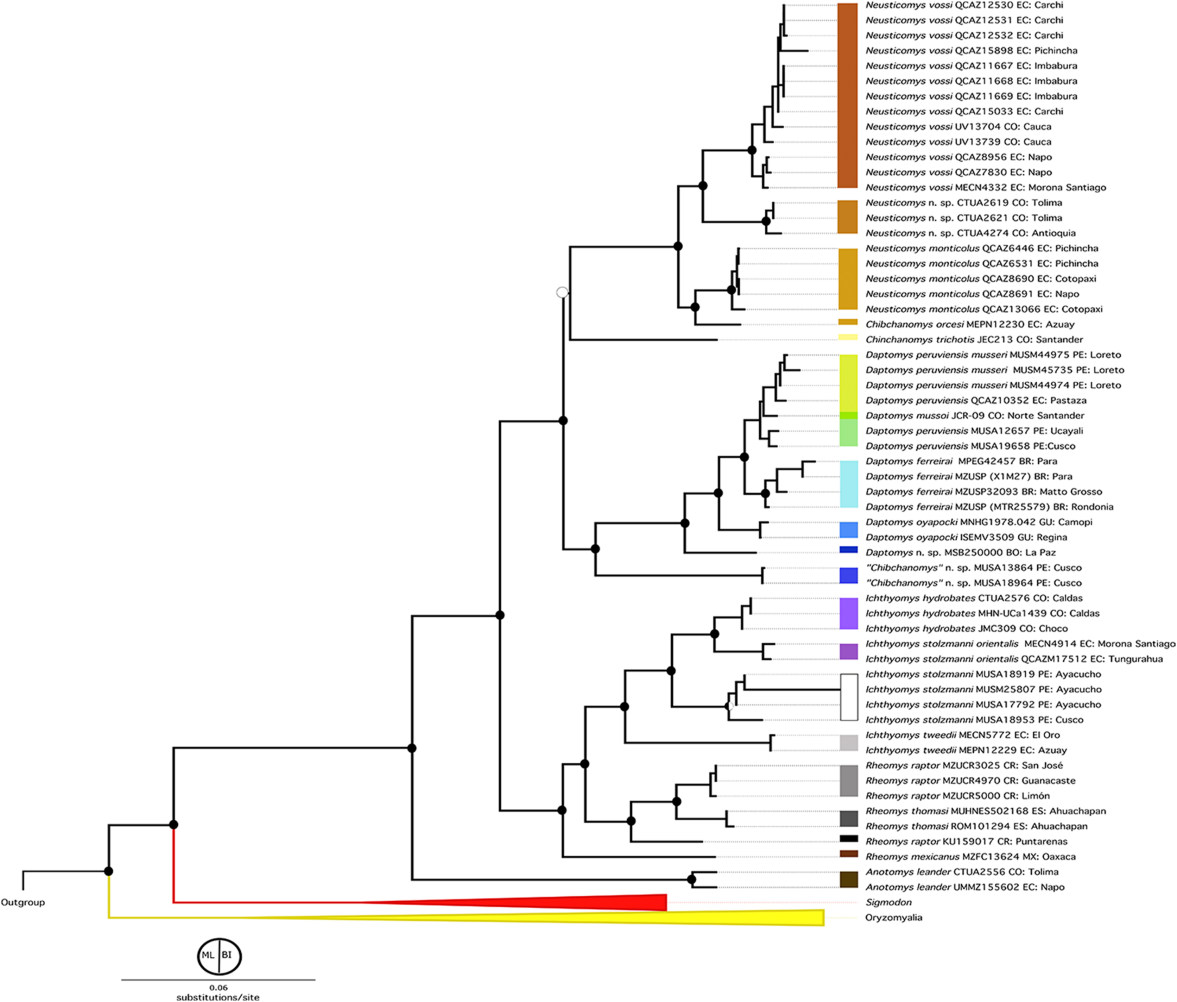
Phylogenetic hypothesis (ML) for the Ichthyomyini based on a concatenated database of molecular characters. Maximum likelihood phylogenetic inference for the Ichthyomyini, based on the combined analysis of one mitochondrial (*Cytb*) and three nuclear markers (*RBP3*, *RAG1*, and *GHR*) for 59 ichthyomyine specimens and multiple outgroup taxa in IQ-TREE. Using the same best-fit model selected by PartitionFinder, Log-likelihood of the tree: −6,3187.3930 (s.e. 1,387.0787). Nodal support from the ML bootstrap pseudo-replicates are indicated at each node along with Bayesian posterior probabilities (BI). White wedges indicate bootstrap values ≤ 75% and black indicates bootstrap frequencies ≥ 75%. For BI, white indicates PP < 0.95, whereas black indicates PP ≥ 0.95. Scale at the bottom represents substitutions per site. Terminals are named with original, museum-based identifications, followed by museum/identification voucher numbers and geographic information (two-letter country code and Department/State). Two-letter country codes as follows: EC, Ecuador; CO, Colombia; PE, Peru; BR, Brazil; GU, French Guiana; BO, Bolivia; CR, Costa Rica; ES, El Salvador. Colored boxes next to names indicate presumed species limits, recognized based on original identifications and/or monophyly.

The topologies obtained by both BI and ML analyses were highly congruent and recovered the previously recognized Sigmodontalia and Oryzomyalia as the two major clades at the base of the radiation of Sigmodontinae, each with strong support (Fig. 2). In all analyses, the tribe Ichthyomyini was recovered as a monophyletic group sister to Sigmodontini with strong support (BS = 99/PP = 1).

Within Ichthyomyini, *Anotomys* was recovered as the earliest divergence, with strong nodal support in both approaches. The remaining ichthyomyine taxa were grouped into two clades: one that included *Rheomys* + *Ichthyomys* and one that included *Chibchanomys* (*sensu lato*) + *Neusticomys* (*sensu lato*). *Ichthyomys* was resolved as monophyletic, but *Rheomys* was paraphyletic with respect to *Ichthyomys*, because *Rheomys raptor* + *Rheomys thomasi* was resolved as the sister group to *Ichthyomys*. *Neusticomys* (*sensu lato*) was resolved in two main clades, one comprising Andean species (*Neusticomys sensu stricto*), and the other grouping eastern-Andean and lowland forms (herein referred to *Daptomys*; see Appendix 2). *Chibchanomys* (as currently recognized) is apparently not monophyletic, because the three analyzed species do not form a clade on this tree.

**Appendix 2:** Proposed new classification for the Tribe Ichthyomyini

New classificatory arrangement for the Tribe Ichthyomyini based on total evidence phylogenetic analysis, with the definition of the taxa recognized, including diagnosis, contents, and remarks.

Within *Rheomys*, specimens currently identified as *R*. *raptor* from the Costa Rican localities of Guanacaste, Limón and San José were recovered as more closely related to *R. thomasi* from El Salvador (as represented by two individuals from different localities), than to another Costa Rican specimen identified as *R. raptor* from Puntarenas (KU159017). Within *Ichthyomys*, our analyses yielded four well-defined clades, most of them with high nodal support. The first one, sister to the remaining species, included samples from southwestern Ecuador assigned to *I*. *tweedii*. The second comprises samples identified as *I*. *stolzmanni* from the Peruvian departments of Ayacucho and Cusco; the third grouped samples from the Colombian departments of Chocó and Caldas identified as *I*. *hydrobates*; and the fourth clustered eastern-Andean foothill samples that are currently identified as *I*. *stolzmanni orientalis*.

Specimens resembling *Chibchanomys* from southeastern Peru (two MUSA specimens herein considered to represent an undescribed species) and *C. trichotis* (the type species of *Chibchanomys*, represented in our study by JEC213, a specimen from northeastern Colombia) were not resolved within the same clade. In fact, the MUSA specimens were retrieved (with strong support) as the sister group to *Daptomys*, whereas *Chibchanomys trichotis* was weakly resolved as the sister species of *Neusticomys* + *Chibchanomys orcesi*. Patterns of relationships within *Neusticomys* and *Daptomys* additionally suggest the presence of currently unrecognized species diversity. Within *Neusticomys*, our sampling of individuals and localities of nominal taxa suggests the existence of three well supported lineages, with *N*. *monticolus* (*sensu stricto*) sister to *Chibchanomys orcesi*, plus a clade formed by an unnamed taxon from the Colombian departments of Antioquía and Caldas that is closely related to *N. vossi* from Ecuador.

Within the strongly supported (BS = 100/PP = 1.0) genus *Daptomys*, our analyses recovered a first split leading to an undescribed species from the cloud forest of the Bolivian Department of La Paz. The latter is sister to a larger clade including *D*. *oyapocki* from Guyana, which is sister to a clade formed by *D*. *ferreirai*, and a clade grouping samples referred to as *D*. *peruviensis* and *D*. *mussoi*.

### Total-evidence analysis

**Figure 3:**
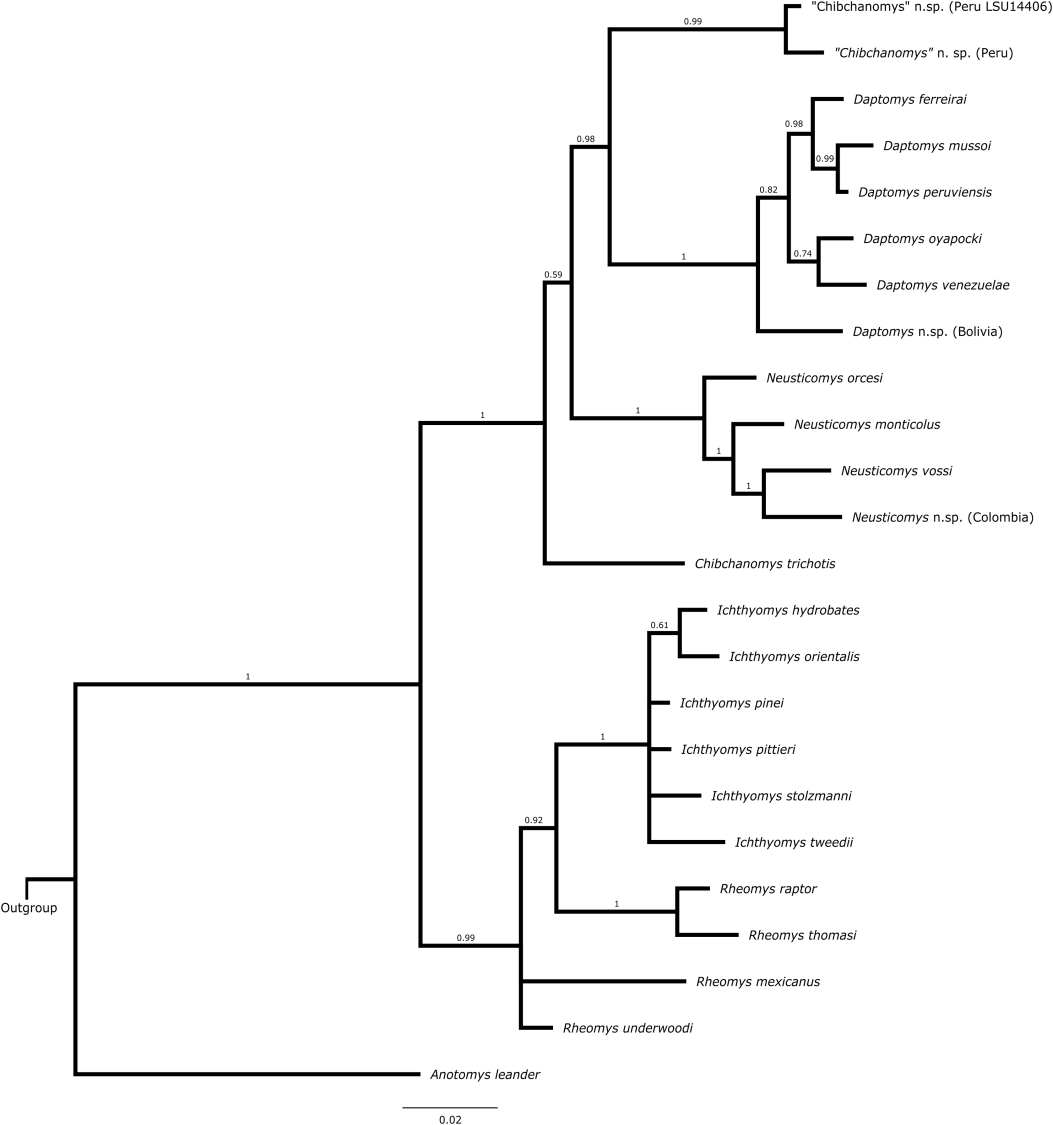
Total evidence phylogenetic analysis for the Ichthyomyini. Bayesian majority-rule consensus depicting the phylogenetic relationships among members of the Ichthyomyini based on combined DNA and morphological data (total evidence approach). A total of 56 morphological characters and four protein-coding genes (4,652 bp) were included in the analysis. Values adjacent to nodes are Bayesian posterior probabilities (PP).

The topology from the total-evidence analysis was partially congruent with those derived from the analyses of molecular-only matrix (Fig. 3). *Anotomys* was recovered as the sister group to all remaining ichthyomyine taxa with strong support. The remaining taxa formed two groups, each with strong support (PP > 0.99): one including a monophyletic *Ichthyomys* and a paraphyletic *Rheomys*, and the other including all the remaining taxa. Within the second clade, *Chibchanomys orcesi* was resolved as closely related to *Neusticomys* with strong support (PP = 1.0), whereas *Daptomys* and “*Chibchanomys*” n.sp. (including LSU 14406) formed another strongly supported group (PP = 0.987). *Chibchanomys trichotis* was also resolved as part of this group, but with weak support (PP = 0.59).

### Estimates of diversification parameters

**Figure 4:**
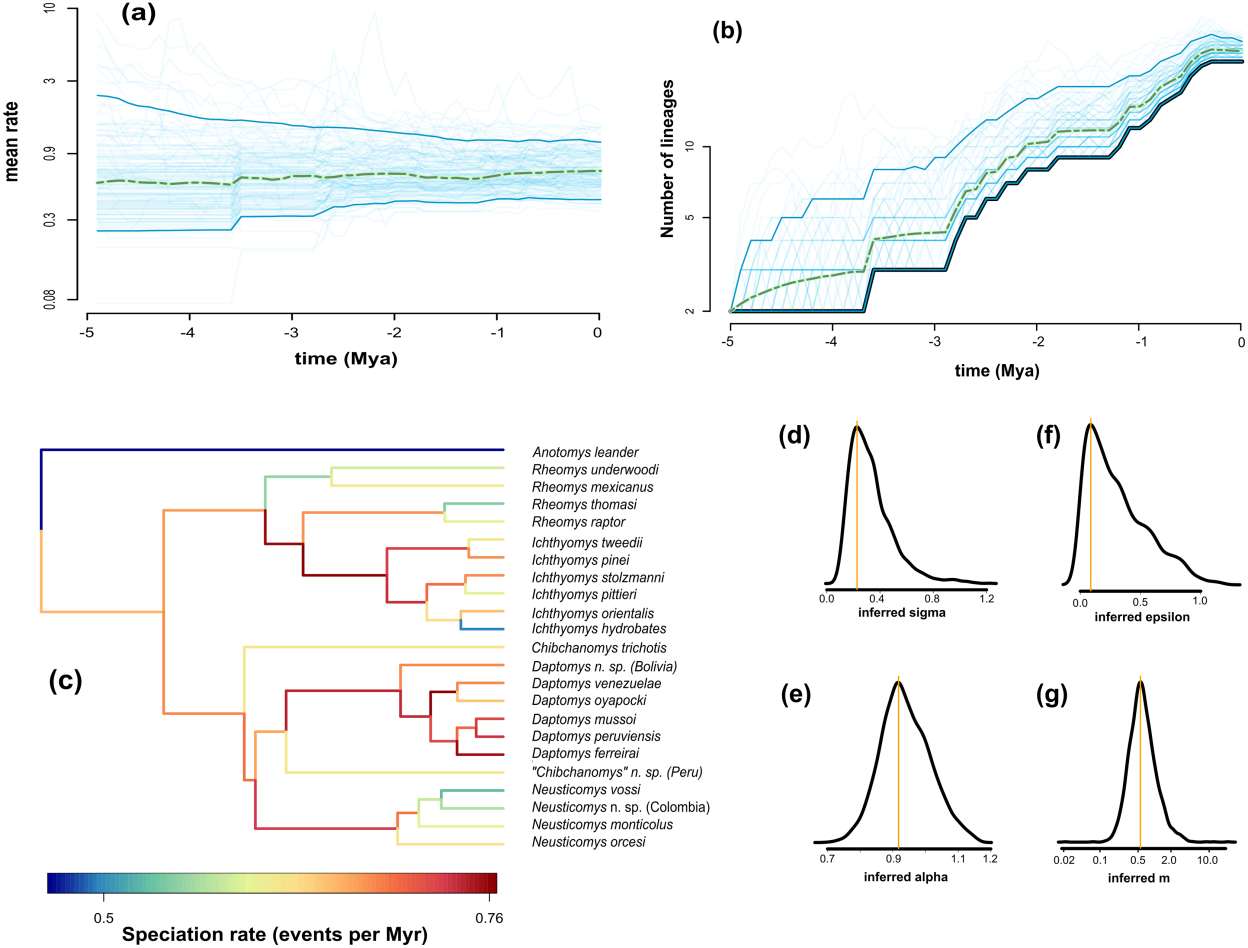
Fit of ClaDS to the Ichthyomyini. Inferred mean speciation rate (A) and number of lineages (B) through time, with individual MCMC iterations (thin blue line), the 95% credibility interval for each time point (thick blue line), and the mean for each time point (dotted green). Time runs from the past (starting at 0) to the present (crown age, here 5 Myrs). (C) Inferred lineage-specific speciation rates for the icthyomyine phylogeny. Marginal posteriors of the heterogeneity parameter *σ* (D), the trend parameter *α* (E), the turnover rate *ε* (F), and the mean change in rate *m* = *αe^σ^*^2/2^ (G). The orange lines indicate the value of the MAP estimate.

We found the variability in speciation rates to be low, as indicated by the σ value (Fig. 4D, σ = 0.23⁠), which is confirmed by a narrow spread of branch-specific speciation rates (Fig. 4C, from 0.41 to 0.78 events per million years). There is a general tendency for rates in daughter lineages to be about the same as in more ancestral ones, as can be seen from the trend parameter α (Fig. 4E, α = 0.92⁠) and the mean relative change in speciation rate m = α×^eσ^2/2^ (Fig. 4G, m = 0.51⁠). The estimated mean speciation rate was relatively constant through time, with only a small but noticeable decline at about 1.5 million years before the present (Fig. 4A); additionally, we found a very low level of extinction (Fig. 4F, ε = 0.089⁠). Consequently, the diversity-through-time curve is very similar to the LTT plot (Fig. 4B). This plot indicated an interval of zero net diversification between 1.1 and 1.8 Mya, which is coincident with the dip in mean speciation rate identified in Fig. 4A.

### Date estimates and biogeography

The topology recovered by BEAST closely matched that of the molecules-only (super matrix) analysis. Sigmodontalia (Ichthyomyini + Sigmodontini) was estimated to have originated in the late Miocene, about 7.89 Mya. The sister group to the Sigmodontalia, the crown Oryzomyalia, was estimated to have originated slightly later at 6.34 Mya. The age of the crown Ichthyomyini was estimated at ca. 4.96 Mya (range 4.16–5.79 Mya); other dates are presented in Table 2 and Fig. S1.

**Table 2:** Divergence times for the most recent common ancestor (MRCA) of representative clades. Summary of mean ages and 95% Highest Posterior Densities (HPDs) estimated with an uncorrelated log normal relaxed clock and FBD diversification model for a combined dataset for the Ichthyomyini and outgroup taxa. Ages in millions of years ago (Mya).

Taxon	Mean age	95% HPDs
Sigmodontinae	8.95	7.75–10.18
Sigmodontalia	7.89	6.74–9.09
Oryzomyalia	6.34	5.80–9.64
Ichthyomyini	4.96	4.16–5.79
*Sigmodon*	2.92	2.76–3.00
Ichthyomyina	3.65	3.13–4.22
*Rheomys* + *Ichthyomys*	2.56	2.07–3.05
*Ichthyomys*	1.25	0.94–1.57
*Chibchanomys trichotis/Neusticomys/* *“Chibchanomys”/Daptomys*	2.78	2.32–3.27
*Daptomys*	1.10	0.84–1.37
*Neusticomys*	1.13	0.86–1.43

**Figure 5:**
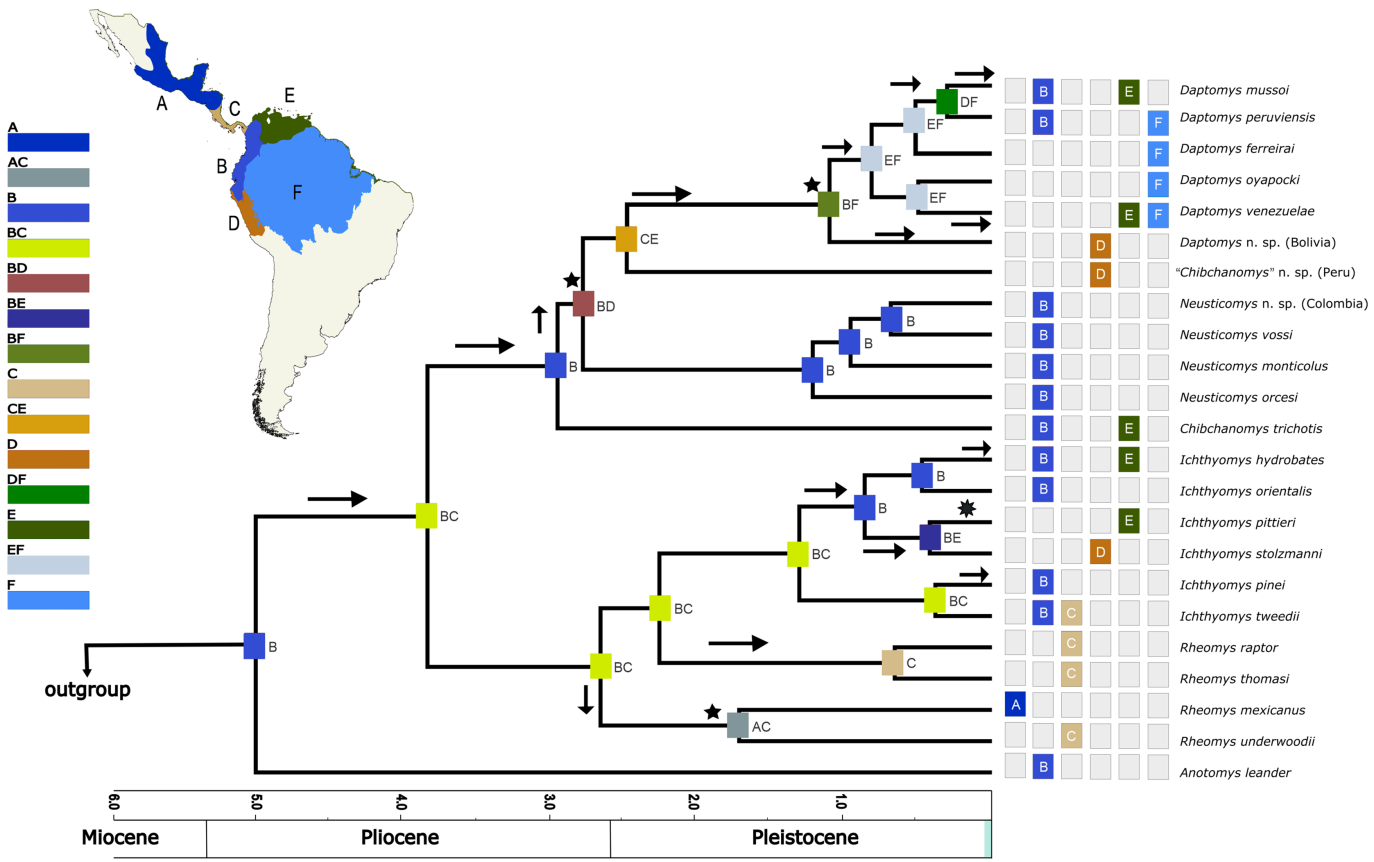
Divergence time estimates obtained from four analyses in BEAST, with ancestral range estimations based on DEC analysis. Bayesian maximum clade credibility chronogram based on the combined analyses of four nuclear, one mitochondrial protein-coding genes, and 56 morphological characters for the Ichthyomyini and outgroup taxa. Hypothesized ancestral biogeographic ranges for Ichthyomyini were estimated based on the dispersal-extinction-cladogenesis (DEC) model as implemented in RASP 4.0. States at nodes represent the ancestral bioregion (or combination thereof) occupied immediately before diversification. Arrows indicate instances where the model suggested diversification *via* dispersal, whereas the stars indicate instances of diversification by ancestral range vicariance. Six-point stars indicate both, suggested speciation by dispersal and vicariance. Numbers above epochs indicate Ma. The blue box next to Pleistocene indicates the Holocene.

Based on the AICc_wt, the model that best fitted the data was DEC (Table 3). The results from the DEC analyses (Fig. 5) suggested the northern Andes (area B) as the most-likely area for the origin of the Ichthyomyini in general and for the genus *Anotomys*, in particular, but the northern Andes plus southern Central America (region BC) for the origin of the remainder of the tribe. Most processes of diversification resulted from dispersal events (arrows in Fig. 5).

**Table 3:** Comparison of the different models of ancestral range estimation performed with BioGeoBEARS. The model that best fits the data (highlighted in bold and itallics below) was DEC, and was therefore the model used to reconstruct the ancestral ranges in the evolution of the tribe Ichthyomyini.

Model	LnL	P	d	e	j	AICc	AIC_wt
*DEC*	***−49.87***	***2***	***0.14***	***0.047***	***0***	***104.3***	***0.96***
DEC + J	−51.72	3	0.044	1.0e−12	0.037	110.7	0.040
DIVALIKE	−57.31	2	0.086	0.0095	0	119.2	0.0006
DIVALIKE + J	−54.33	3	0.051	1.0e−12	0.035	115.9	0.0029
BAYAREALIKE	−62.61	2	0.15	0.37	0	129.8	2.8e−06
BAYAREALIKE + J	−56.95	3	0.034	1.0e−07	0.053	121.2	0.0002

**Note:**

LnL, ln(likelihood); # Params, = Number of parameters; d, dispersal rate (*i.e*., the rate of range addition along a phylogenetic branch); e, extinction (*i.e*., the rate of local range loss along a phylogenetic branch); AICc, corrected Akaike Information Criterion; AICc_wt, weight of the models based on the AICc.

**Figure 6:**
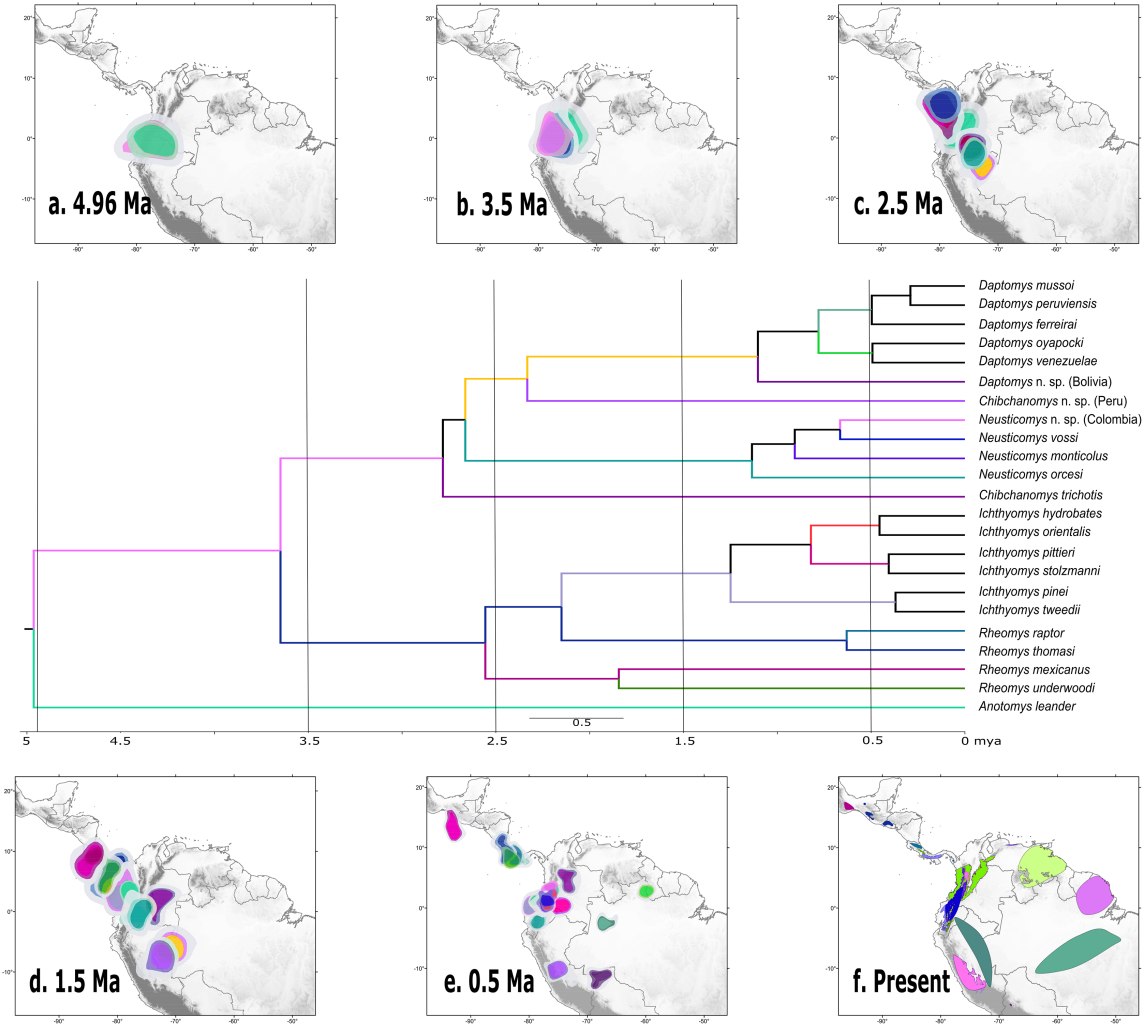
Ancestral ranges in the evolution of the Ichthyomyini estimated using *rase*. Ancestral estimation according to *rase* at six time slices, from 4.96 Ma to the current distribution of members of the tribe Ichthyomyini. (A–E) Plot the 10%, 15%, and 25% highest posterior density for each extant branch (10% with highest color intensity). (F) plots current species distributions. The branches of the phylogenetic tree are color coded as in (A–F) to identify its position in the map. For further discussion, see main text.

According to the *rase* results, the ancestor of the Ichthyomyini was distributed in an area presently covering the foothills of the Nudo de Pasto (Fig. 6A), at a latitudinal mean (latM) of 0.8252 and a longitudinal mean (lonM) of −76.95. The ancestor of the lineage leading to the remainder of the Ichthyomyini (other than *Anotomys*) originated in the same general area (latM = 0.6077, lonM = −76.92), but apparently about 1.3 Mya later (Fig. 6B). The ancestor of *Rheomys* + *Ichthyomys* underwent a north-westward migration (Fig. 6C; turquoise), while the ancestor of *Chibchanomys* + *Neusticomys sensu* lato migrated south-eastward (Fig. 6D; purple). The estimated dispersal rates for ichthyomyines were about 44% greater for longitudinal (mean of 51.95 degrees²/Ma, SD = 8.95) than latitudinal displacements (mean of 35.95 degrees²/Ma, SD = 8.26).

## Discussion

The tribe Ichthyomyini was the first suprageneric group of sigmodontine rodents to receive a modern and thorough systematic revision (Voss, 1988). In that study, tribal monophyly was supported by a number of morphological characters (*e.g*., the disposition of the mystacial vibrissae, possession of well-developed masseteric tubercles, the contents of the infraorbital foramen, the attachment of the nuchal ligament to the third thoracic vertebra). These traits were considered to be uniquely derived among the Sigmodontinae (Voss, 1988) despite the fact that no formal phylogenetic analysis was conducted in sufficiently a broad taxonomic context to support relevant character optimizations. Our analyses included molecular data for representatives of all currently recognized ichthyomyine genera (*Anotomys*, *Rheomys*, *Ichthyomys*, *Chibchanomys*, and *Neusticomys*) and 15 of 19 recognized ichtyomyine species. Additionally, all currently recognized ichthyomyine species were scored for an expanded suite of morphological characters that included external, cranial, dental, soft-anatomical, and postcranial traits. Regardless of the optimality criteria used, Ichthyomyini was resolved as a monophyletic group with strong support. Therefore, our results favor the hypothesis that the origin of the suite of character states identified by Voss (1988) as diagnostic for the Ichthyomyini, accompanied the evolution and diversification of the tribe.

The incorporation of morphological data was crucial for assessing the position of four currently recognized taxa lacking molecular data: *Ichthyomys pittieri*, *Ichthyomys pinei*, *Daptomys venezuelae*, and *Rheomys underwoodi*. In addition, the total-evidence analysis resulted in a hypothesis of relationships among ichthyomyine genera and species that was strongly supported at most of the deeper nodes (Fig. 3). In particular, there is strong support for two basal lineages, one composed of the genus *Anotomys* and another that includes the remaining. Consistent with this result, we formally designate *Anotomys* as the type genus of a new subtribe (Anotomyina) and refer the remaining taxa to the subtribe Ichthyomyina (Appendix 2). Additionally, our results justify restoring *Daptomys* to generic status, and they suggest the presence of a new genus from central Peru. These and other taxonomic innovations are discussed at length in Appendix 2.

### Sigmodontalia: a monophyletic, morphologically disparate, supratribal assemblage

Our results strongly support the monophyly of Sigmodontalia as the clade that includes the most recent common ancestor of *Sigmodon* and Ichthyomyini plus all their descendent species. Our analyses estimated a split between Sigmodontini (currently represented only by *Sigmodon*) and Ichthyomyini at some time prior to the estimated time of diversification of the Oryzomyalia, an inference that also agrees with other studies with a much-reduced representation of ichthyomyine taxa (*e.g*., Steppan, Adkins & Anderson, 2004; Schenk, Rowe & Steppan, 2013; Parada, Hanson & D’Elía, 2021).

The extreme morphological disparity between the two lineages of Sigmodontalia has seldom been discussed (but see Weksler, 2003; Pardiñas et al., 2022). This most likely reflects the fact that recognition of the clade is relatively recent and was completely based on molecular data (Weksler, 2003; Jansa & Weksler, 2004; Parada et al., 2013; Leite et al., 2014). *Sigmodon*, a genus currently composed of about 13 recent species of remarkably similar external appearance, consists of quintessential grazing rats with short vibrissae, narrow hindfeet, short tails, and flat-crowned teeth (*e.g*., Voss, 1992, Patton, 2017). In contrast, members of the Ichthyomyini are a diverse assemblage of forms with modifications for freshwater carnivory that include long and abundant mystacial vibrissae, natatory fringes of hair on the margins of webbed hindfeet, and strongly tubercular molars (Voss, 1988). The morphological distinction between these two groups is also mirrored by their ecological differences. Species in *Sigmodon* are terrestrial rats, mostly associated with open-vegetation formations and grasslands, and with an herbivorous diet restricted to grasses and forbs (Voss, 1992). By contrast, ichthyomyines are semi-aquatic rats, found along streams bordered by evergreen rain forest, and with carnivorous diets (Voss, 1988).

Weksler (2003), who first intimated the monophyletic nature of the Sigmodontalia based on molecular characters, suggested the presence of lateral crater papillae on the phalli as a putative, non-homoplastic, morphological synapomorphy for the clade based on work reported by Hooper (1959), Hooper & Musser (1964) and Voss (1988). In a much more comprehensive cladistic analysis of morphological characters, Pacheco (2003) suggested that the presence of lateral crater papillae in sigmodonts and ichthyomyine taxa was likely a non-homologous character convergence. The lack of morphological character data for our outgroup taxa prevents us from formally addressing this point by the optimization of this character change on the phylogeny we recovered. However, as Pacheco (2003) indicated “…all sigmodontines other than ichthyomyines and *Sigmodon* lack these papillae”. Further, an examination of his Appendix 2 (Taxon-Character Matrix) shows that, except for *Nelsonia neotomodon* for whom this character state is missing, all important outgroup taxa (including Tylomyinae), also lack these papillae. Therefore, we agree with Weksler (2003) in that the presence of lateral crater papillae on the phalli of sigmodonts and icthyomyines should be considered as a synapomorphy for the Sigmodontalia.

The clear disparity in morphological diversity between these sister taxa evokes the signal of an adaptive radiation in the Ichthyomyini, likely the result of ecological opportunity (EO). EO is defined as any event that could make niche space available, including the emergence of key innovations that allow species to interact with the environment in a novel way (*i.e*., new niche dimensions; Stroud & Losos, 2016). Importantly, the ecological theory of adaptive radiation makes two main predictions: (a) an early burst of lineage diversification, accompanied by divergence in ecologically relevant phenotypic traits, (b) followed usually by a density-dependent slowdown in the rate of speciation and phenotypic diversification resulting from increased competition as ecological niches become filled (*e.g*., Schluter, 1996; Gavrilets & Losos, 2009; Glor, 2010; Yoder et al., 2010). Our results from the Diversification Through Time analyses do not appear to provide compelling evidence for the existence of bursts of lineage diversification in the ichthyomyine radiation. In fact, our analyses highlight low variability in the speciation rates through time, which is also supported by a narrow spread of branch-specific rates (Fig. 4). This suggest that ichthyomyine lineages have accumulated roughly as expected given constant rates of lineage birth and death through time (with a small but significant decline at 1.5 Mya).

Given the unique ecological niche occupied by the Ichthyomyini and the morphological diversity present within the group, in particular when compared to the Sigmodontini, these results are surprising. However, morphological diversity can theoretically be unrelated to rates of diversification (Adams et al., 2009), and comparative analyses across muroid rodents on continents suggests that phylogenetic diversity and phenotypic disparity may be decoupled (Alhajeri, Schenk & Steppan, 2016). For the entire sigmodontine radiation studies have shown the signal of early strong diversification (Fabre et al., 2012, Steppan & Schenk, 2017), much of which may have been associated with biogeographic regional transitions in South America (Schenk & Steppan, 2018). However, studies that incorporated the analyses of diversification rates and morphological disparity argue that non-adaptive processes—biogeographical history and community ecology—explain the high diversity currently observed in the group without the need to invoke adaptive radiation processes (Maestri et al., 2017; Maestri, Upham & Patterson, 2019). Detailed analyses of morphological and ecological diversification in the ichthyomyine radiation, in the phylogenetic, temporal, and geographic context investigated herein is the subject of a forthcoming companion paper.

### Divergence-date estimation and biogeography

We calibrated the phylogeny in this study with 10 fossil constraints, six of which have never been used in previous attempts to date the diversification of the Sigmodontinae. Our estimated divergence dates match reasonably well with recently published estimates based on the analyses of much larger datasets and different calibration points, for example Steppan & Schenk (2017) and Parada, Hanson & D’Elía (2021). Divergence-time estimates derived from this analysis suggest that the basal split within the Sigmodontinae crown clade occurred in the late Miocene (8.95 Mya; 95% credible interval = 7.75–10.18, Fig. S1). The basal split within Ichthyomyini crown clade occurred in the early Pliocene (4.96 Mya; 95% credible interval = 4.16–5.79, Fig. S1).

Our biogeographical analyses strongly suggest a northern-Andean origin for the ichthyomyine crowngroup, with the ancestral areas of the basal nodes for *Anotomys* and subsequently for *Daptomys* + *Chibchanomys* + *Neusticomys* in the same general area, whereas the area of origin for *Rheomys* + *Ichthyomys* comprises both the Northern Andes and the southern portion of Central America. Ichthyomyine diversity is higher in the northern Andes than anywhere else in the Neotropics (Voss, 1988, 2015), a pattern that had already been recognized by Reig (1984, 1986). In the context of tribal phylogeny presented here, however, the lowlands on the periphery of the Amazon basin are also of high diversity, currently with six species, all of them belonging to *Daptomys*.

### Comparison in the tempo and area of diversification of the members of the Ichtyomyini and the Oryzomyini

The most diverse tribe within the Sigmodontinae is the Oryzomyini, with 31 genera and 148 living species (Brito et al., 2020; Percequillo et al., 2021). Despite the differences on species numbers, a comparison between the biogeographic patterns of the Oryzomyini and the Ichtyomyini is relevant, as these groups share three important characteristics: (a) their distributions include North, Central and South America, (b) a deep history related to the northern Andes, and (c) a complex history of inter-American movements. While the first remains true and the second has been questioned, our data allow the third aspect to be further evaluated here. Prior discussions on the time and diversification of either clade were completed without the benefit of a time calibrated phylogeny for ichthyomyines (*e.g*., Hershkovitz, 1966; Weksler, 2006; Voss, 1988; Percequillo et al., 2021).

Within oryzomyines, *Oryzomys* and “*Handleyomys*” (*sensu* Weksler, 2015) are predominantly North and Central American lineages, like *Rheomys* is for the ichthyomyines; the two mentioned genera of oryzomyines are more diverse, with few species occurring in South America, while *Rheomys* has no representatives in the continent. The bulk of the diversity of oryzomyines is widely distributed in all major South American habitats and biomes, differing from that of the ichthyomyines which is concentrated in the Andes and the Amazon regions. While the northern Andes remain as the potential “area of original differentiation” (AOD, thereafter) for the Ichthyomyini under the rigor of phylogenetic and biogeographic methods and concepts here employed, the same approaches on oryzomyines revealed that the ancestral area for the tribe lies on the Amazon basin (**e.g*.*, Maestri, Upham & Patterson, 2019). A re-evaluation on the patterns of species diversity for oryzomyines (*e.g*., Valencia-Pacheco et al., 2011; Prado & Percequillo, 2013; Prado et al., 2015) pointed that the Amazon basin is nearly as species-rich as the northern Andes, which questions the latter’s presumptive role as AOD for the tribe. As with ichthyomyines, with three genera endemic to this region (50% of generic diversity), oryzomyines, with seven genera, also exhibit great diversity in the northern Andes (24% of generic diversity).

Soon after the origin of *Anotomys* in the northern Andes (4.16–5.79 Mya), the ancestor of the remaining ichthyomyine lineages occupied an area formed by the northern Andes plus Central America. From this region two lineages diverged (3.13–4.22 Mya): one gave origin to *Neusticomys*, *Chibchanomys thrichotis* and *Daptomys* (around 2.78 Mya), lineages that never invaded Central or North America again. The other lineage, whose ancestor occupied a broader area (northern Andes + Central America), diversified in part of the genus *Rheomys* (*R. underwoodi* + *R. mexicanus*) around 2.56 Mya, subsequently invaded North America, originating the remaining species of the genus *Rheomys* (ca. 0.63 Mya), and later colonized South America again (the northern Andes) and gave rise to *Ichthyomys* (around 1.25 Mya). Thus, this radiation that originated in South America, dispersed only once to Central and North America, where it experienced a moderate level of diversification.

Oryzomyines, and the ancestors of most internal clades (*Scolomys*, *Zygodontomys*, groups “B,” and “D” *sensu* Weksler, 2006) on the other hand most likely originated in the Boreal Brazilian region (the north bank of Rio Amazonas and the Guiana Shield) in the Miocene (8.93–5.38 million years ago, Percequillo et al., 2021). From here, there were multiple invasions to the northern Andes and also multiple invasions of Central and North America, and at different times throughout the evolution of the group (see Percequillo et al., 2021), supporting the observation that “current oryzomyine diversity in Central and North America is the product of independent colonizations made by the different clades within oryzomyines that are currently found in that area” (Weksler, 2006: 89). Therefore, although similar in some aspects of their patterns of distribution, the biogeographic history of these two tribes are quite distinct. Of notice, the estimated divergence time for the Ichthyomyini (4.16–5.79 Mya) matches reasonably well with those estimates for the divergence of major clades in the Oryzomyini (clades B(C+D) and C+D, see Percequillo et al., 2021: Table 1) in the late Miocene to early Pliocene.

### The long-fuse hypothesis for the evolution of the Ichthyomyini

Our results suggest that most of the processes of diversification in Ichthyomyini were the result of dispersal events more than vicariance-based processes, although we acknowledge the oversimplification of this dichotomy (as in Matzke, 2013). The *rase* analyses identified the origin of the ichthyomyine crowngroup in the northern Andes. The bulk of the ichthyomyine radiation, which is estimated to have started at around 4.96 Mya, appear to have been restricted to the northern Andes for at least 2.5 Mya (Figs. 6A–6C). After the mark of the 2.5 Mya, the tribe started diversifying in earnest and the number of forms doubled to about six major lineages that gave rise to the diversity we currently observe (Fig. 6). In summary, our data suggest that the ichthyomyine diversification was circumscribed to the northern Andes for about half of their evolutionary history (a long fuse). Moreover, our analyses also suggest that speciation rates were relatively constant through time, with only a small but noticeable decline at about 1.5 million years before the present, and very low levels of extinction. Although these statements are tempered by the almost complete absence of the fossil record—we have no direct way of estimating the effect of extinctions on the true dynamics of diversification (speciation-extinction)—it is instructive to consider the extrinsic and intrinsic factors associated with this pattern.

In the northern Central and northern Andes (north of 20°S), present day elevations were achieved during or soon after the late Miocene (Garzione et al., 2008; Gregory-Wodzicki, 2000), and there is evidence that the northern Andes reached c. 4,000 m, their maximum mean paleo-elevation at 6 Mya (Bermúdez et al., 2015). This means that the central portion of the northern Andes was likely at its current elevation and with somewhat similar environments to current day páramos (Ochoa et al., 2012) when Ichthyomyini started its process of diversification. Andean orogeny in Ecuador, Colombia, and Venezuela was likely influenced by collision with island arcs and oceanic plateaus (Iturralde-Vinent & MacPhee, 1999; Londoño, Cleef & Madrinan, 2014; O’Dea et al., 2016; Prevosti & Forasiepi, 2018) with the concomitant formation of stratovolcanos and extensive production of volcanic rocks and pyroclastic materials (Hall, 1977). In Colombia, the rise of the Eastern Cordillera, involved several steps creating a mosaic of habitats, both in terms of elevational ranges as well as the expansion of high elevation habitats (Bermúdez et al., 2015).

Under this scenario, we hypothesize that the Ichthyomyini evolved in the northern Andes, and that its original radiation was triggered by the progressive invasion into exclusive Andean environments. This evolutionary process also affected several other members of the Sigmodontinae, largely including Thomasomyini (*e.g*., *Aepeomys*, *Chilomys*, *Thomasomys*), Oryzomyini (*e.g*., *Mindomys*, *Nephelomys*, *Pattonimus*, *Tanyuromys*), Neomicroxini (*i.e*., *Neomicroxus*) (Reig, 1986; Voss, 2003; Pine, Timm & Weksler, 2012; Timm, Pine & Hanson, 2018; Brito et al., 2020; Cañón et al., 2020; Percequillo et al., 2021), as well as north Andean echimyids (Fabre et al., 2017).

After analyzing the patterns of morphological variation among and between populations of ichthyomyine rodents, Voss (1988) concluded that elevation-related ecological conditions (temperature, flow rate) imposed stringent adaptive constraints on the cranio-skeletal evolution of these cricetids and acted synergistically to determine specific morphotypes. He further hypothesized that “primitive ichthyomyines were short-tailed with short distal limb elements, small hindfeet, incisor-emphasized dentitions, and uninflated braincases” (Voss, 1988: 469), and suggested that *Daptomys venezuelae*, provided “a credible living model of the ancestral phenotype” (Voss, 1988: 469). The implication from his statements is that evolution worked from taxa with putative plesiomorphic traits (that is, with no or few specializations for aquatic living), occurring in lowland habitats, to forms that were much more specialized and highly adapted to an amphibious carnivory lifestyle in more highland environments. Our results do not support the hypothesis of an overall gradualistic directional change in the morphological evolution of the tribe espoused by Voss (1988:469). Instead, our time-calibrated phylogeny suggests that the morphotype associated with highly specialized amphibious carnivory evolved on several occasions throughout the evolutionary history of the Ichthyomyini: in *Anotomys*, in *Rheomys* + *Ichthyomys*, and in *Chibchanomys*. Furthermore, in at least one instance, clades that include groups with highly specialized traits for amphibious carnivory (*e.g*., *Rheomys* and *Ichthyomys*) evolved simultaneously with lineages with less specialized traits (*e.g*., *Daptomys* and *Neusticomys*) in further contradiction to Voss’s hypothesis.

Finally, an aspect that merits comment is the statement that evolutionary rates are faster in invertebrate-eaters (Martinez et al., 2018, 2020). Ichthyomyines constitute an extreme experiment, within sigmodontines, specialized on amphibious carnivory. As such, they present unique adaptations, including molar loss and an earless condition, strongly paralleling conditions observed in hydromyine murids (Helgen, 2005). The loss of upper third molars is reported most frequently in Amazonian species (*D. ferreirai*, *D. oyapocki*; Percequillo, Carmignotto & Silva, 2005; Catzeflis et al., 2017), suggesting that this trait appeared later in the diversification of the tribe. However, the lack of ears is autapomorphic of *Anotomys*, an early offshoot in the process of diversification in the group. This apparent temporal mosaic in the acquisition of the traits that characterize the ichthyomyine body-plan reflects idiosyncratic rates of morphological change rather than a general process involving all members. Differential rates are more probably associated to several environmental pressures. Again, the lack of fossils for the tribe, except those very late (*e.g*., *Anotomys* in the Holocene of Ecuador; Fejfar et al., 1993), represents a strong impediment to a confident assessment of the patterns of evolutionary change. Importantly, when ancient paleontological evidence is available, for example in *Sigmodon* (Peláez-Campomanes & Martin, 2005), it more often reflects morphological stasis than rapid changes. Certainly, *Sigmodon* as a mostly grass and forbs eater (Cameron & Spencer, 1981; Martinez-Chapital et al., 2017), is inadequate to contrast predictions associated to animalivorous rodents. Conversely, *Onychomys* a specialized invertebrate–eater, representing an old tribal-lineage within the Neotominae (see Kelly et al., 2022), shows no substantial dentary change since the Pliocene (Carleton & Eshelman, 1979).

With the valuable results obtained here, product of the collaborative effort of several researchers integrating an international and interdisciplinary team, a new tribe of sigmodontines is added to those that received a detailed exploration combining morphological and genetic evidence. Now, Ichthyomyini can be fully compared with the previously addressed Oryzomyini (Weksler, 2006; Pine, Timm & Weksler, 2012; Percequillo et al., 2021), Phyllotini (Steppan, 1995; Salazar-Bravo, Pardiñas & D’Elía, 2013; Carrizo & Catalano, 2015), and Abrotrichini (Teta et al., 2017). Several other diverse tribes (*e.g*., Akodontini, Thomasomyini) are waiting similar approaches to produce a well-supported integrative phylogeny of the subfamily.

## Appendix 1

List of the morphological characters used in the phylogenetic analyses. Unless otherwise noted, all characters describe conditions for adult individuals. Basic source for morphological information about ichthyomyines is the comprehensive monograph of Voss (1988) complemented, when needed, by Anthony (1929). For most of the listed characters, a brief description is provided and, if necessary, illustrations are also included.

### General morphology

Character 1. Body size: (0) small (<~50 g); (1) large (>~50 g). Body mass is seldom reported in ichthyomyines, even in recently collected animals (*e.g*., Pacheco & Ugarte-Núñez, 2011; Santillán & Segovia, 2013; Marín-C, Corrales-E & Valencia, 2018); in addition, some degree of spurious increment on this measurement is expected because most animals are primarily recorded wet. According to the available data, weights ranged from about 15 to 140 g (*e.g*., Voss, 1988; Leite, da Silva & Gardner, 2007; Brito & Ojala-Barbour, 2016; Percequillo et al., 2017; Table 2). To divide this universe in two states is a conservative approach, only possible after a preliminary exploration of available masses indicated a smooth bimodal distribution. To further explore weight distribution in the ingroup, we plotted the standardized log transformed weight data for 77 individuals of 17 species of ichthyomyines (Table S1). This plot showed a normal distribution with an average log(weigth) = 1.65814. Back transformed, this value corresponds to a body mas of 47.4 g. A conservative approach was to use the rounded-up value of 50 g as a cut-off. Most of the ichthyomyines are below 50 g (and, even indeed, 30 g), including all the members of *Anotomys*, *Chibchanomys*, *Neusticomys* (except *N. venezuelae*) and some *Rheomys* (*i.e*., *R. raptor* and *R. thomasi*). Above 50 g are the species of *Ichthyomys*, even including the smaller representatives, such as *I. pittieri* (>65 g; see García et al., 2012). In addition, two species of *Rheomys*, *R. mexicanus* and *R. underwoodi*, are heavier than 50 g (*R. mexicanus*, for example, was recorded with 88 g; see Santos-Moreno et al., 2003).

### External morphology

Character 2. Body pelage: (0) pelage glossy and grizzled-brownish; (1) pelage dull and gray-black. The description and coding of this character follows Voss (1988, ch. 1).

Character 3. Guard hairs: (0) comparatively few in number or not abundant; (1) long and abundant. Perceptible differences in the amount and quality of guard hairs were highlighted by Anthony (1929), also in association with the “hispid” or “soft” condition of the whole pelage. Abundant guard-hairs interspersed with wool hairs are found in *Ichthyomys* spp., *Rheomys mexicanus* and *Rheomys underwoodi*; the remainder taxa are characterized by few fine scattered guard hairs.

Character 4. Ventral countershading: (0) pelage not countershaded; (1) pelage lightly countershaded; (2) pelage countershaded. Based on Voss (1988, ch. 2), but modified to three states. Ventral fur colored like dorsal fur is found in species of high-Andean and lowland *Neusticomys*, *Chibchanomys* n. sp., and *Rheomys raptor*. Lightly countershaded with under parts washed with pale gray, or silvery fur, or even copper colored is recorded in *Chibchanomys orcesi*, *Rheomys underwoodi*, and some Amazonian *Neusticomys* (*N. peruviensis*, *N. oyapocki*, *N. ferreirai*). Finally, pelage countershaded with underparts white or cream different of blackish, darker or grey-brown dorsum characterizes *Anotomys*, *C. trichotis*, *Ichthyomys* and *Rheomys mexicanus* and *R. underwoodi*. A perceptible degree of association between this trait and general body shape can be argued, being markedly countershaded those forms with deeper aquatic specializations.

**Figure 7:**
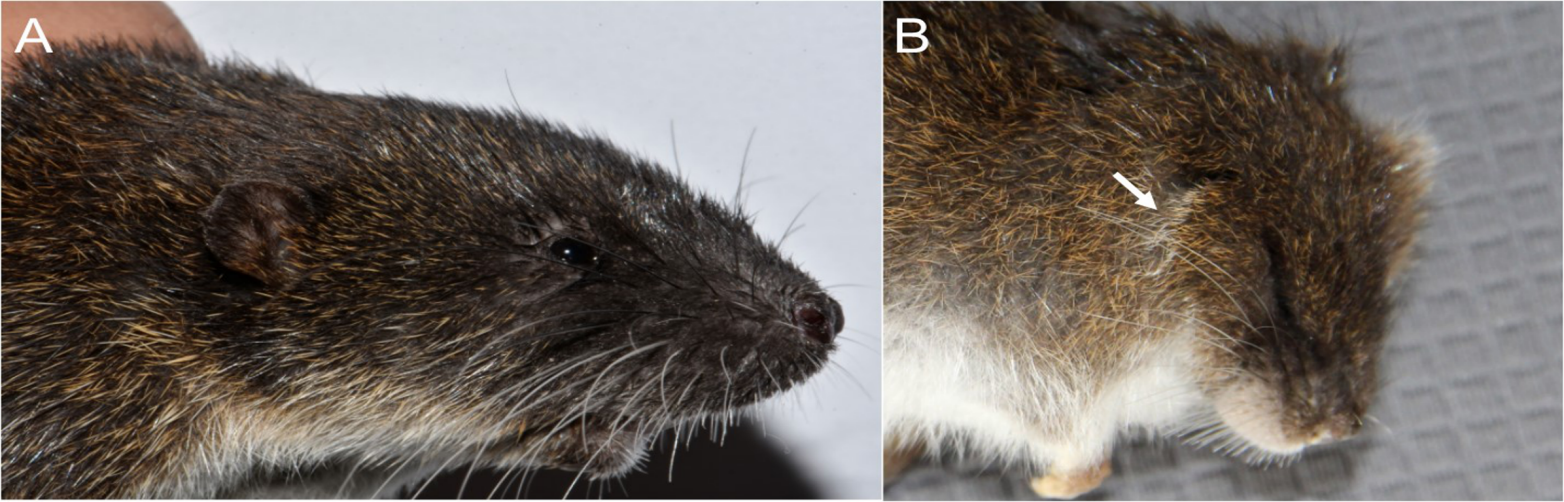
Details of the head in two species of ichthyomyine rodents. The figure illustrates the variation on the presence of pale spots on the auricular region in species of the Ichthyomyini (character 5). (A) Absent (*Ichthyomys tweedii*; MECN 5772); (B) present (*Rheomys underwoodi*; APG sn).

Character 5. Pale spots on auricular region: (0) absent; (1) present. Previous authors (Braun, 1993; Weksler, 2006) treated differentially pre-post- or subauricular patches of whitish or pale hairs in reference to phyllotines and oryzomyines. Here we opted for a general location of this spots “on the auricular region,” as in one of the most conspicuous examples of the presence of this trait lacks pinnae (*Anotomys*). *Ichthyomys stolzmanni* is polymorphic for this trait. The remaining taxa lack auricular spots (Fig. 7).

**Figure 8:**
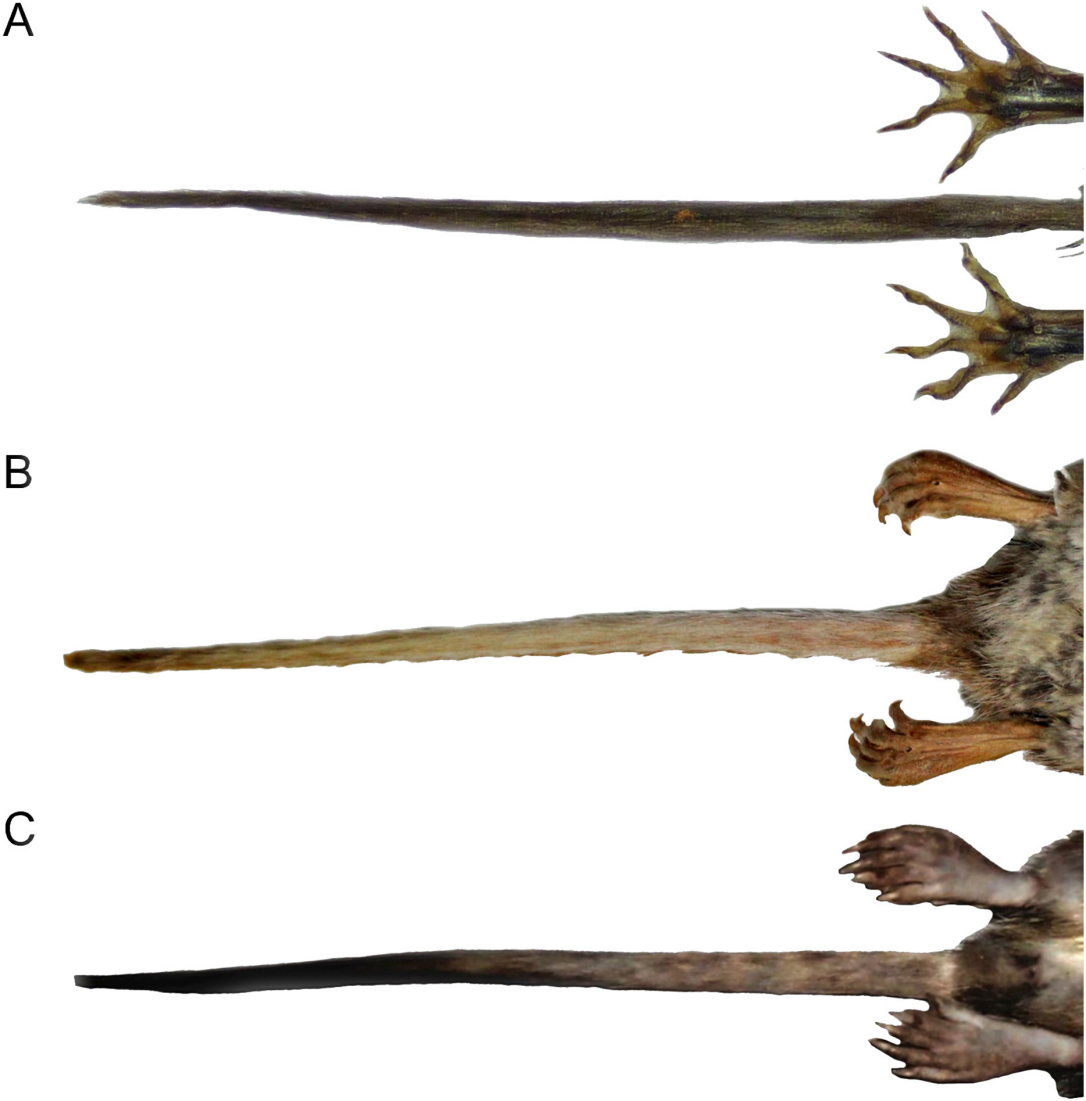
Details of the ventral tail coloration in the Ichthyomyini. The figure illustrates the three types of ventral tail coloration in the Icthyomyini (character 6). (A) Unicolored (*Rheomys raptor*; MZUCR 5000); (B) entirely bicolored (*Ichthyomys pinei*; MZUA 234); (C) bicolored except distal inch (*Ichthyomys orientalis*; MECN 4914).

Character 6. Tail color: (0) unicolored dorsoventrally; (1) entirely bicolored dorsoventrally; (2) bicolored dorsoventrally, except distal inch. Based on Voss (1988, ch. 3), but modified to three states (Fig. 8). Although the coloration of the tail was traditionally employed in ichthyomyine systematics, this character is probably affected by ontogenetic and individual variability. Our state 2 was added to describe the condition recorded in some species of *Ichthyomys* which exhibit the distal inch of the tail darker all-around (*e.g*., Férnandez de Córdova et al., 2020). Strongly bicolored tails are exclusive of a few ichthyomyines such as some species of *Ichthyomys* and the two large-bodied *Rheomys* (*i.e*., *R. mexicanus* and *R. underwoodi*).

Character 7. Philtrum: (0) present; (1) absent. The description and coding of this character follows Voss (1988, ch. 4). The philtrum is absent in *Anotomys*, *Chibchanomys*, and the two large-bodied *Rheomys* (*i.e*., *R. mexicanus* and *R. underwoodi*).

Character 8. Pinnae: (0) large, visible above the fur of the head; (1) small, concealed beneath the fur of the head; (2) absent. Based on Voss (1988, ch. 3), but modified to three states (see also Anthony, 1929; Ellerman, 1941). Large or “complete” pinnae, showing a structural pattern similar to other sigmodontine rodents are found in *Neusticomys* and the two small-bodied *Rheomys* (*i.e*., *Rheomys thomasi* and *Rheomys raptor*). A small and incomplete ear, concealed in fur is found in *Chibchanomys*, *Ichthyomys*, and large-bodied *Rheomys*. *Anotomys leander* is the single example of an ichthyomyine (and even, a sigmodontine) without pinnae.

Character 9. Superciliary vibrissae: (0) present; (1) absent. The description and coding of this character follows Voss (1988, ch. 6). In the context of the tribe, the presence of these vibrissae is an autapomorphic condition of *Anotomys*.

Character 10. Plantar pads of manus: (0) five pads; (1) four pads; (2) three pads. The description, partially simplified here, and coding of this character follows Voss (1988, ch. 7). Five pads, with hypothenar pad not fused with the third interdigital, are found in *Chibchanomys*, *Ichthyomys*, and species of *Neusticomys*. Four pads, with hypothenar and third interdigital pads fused, is exclusive of *Anotomys*. Three pads, with the hypothenar and thenar pads fused with adjacent third and first interdigital pads, respectively, is found in *Rheomys*.

Character 11. Metatarsal configuration: (0) III≥IV>II>>V>I; (1) IV>III>V>II>I; (2) IV>III>II≥V>I. Based on Voss (1988, ch. 14), but modified to three states following Starrett & Fisler (1970). The state (0) is found in species of *Neusticomys* and in *Chibchanomys orcesi*; the state (1) is exclusive of *Anotomys leander* and *Rheomys thomasi*; the state (2) is exhibited by *Chibchanomys trichotis* and species of the genera *Rheomys* and *Ichthyomys*.

Character 12. Fringes with stiff hairs on the margins of pes: (0) weakly developed; (1) well developed. The description and coding of these natatory fringes follows Voss (1988, ch. 8). The weakly developed stiff hairs along of the border of pes and digits exhibits less than 2 mm in length and are found in *Chibchanomys orcesi*, *Neusticomys*, and small-bodied *Rheomys*. Well-developed stiff hairs are longer than 2.5 mm, being the condition exhibited by *Anotomys leander*, species of *Chibchanomys* and *Ichthyomys*, and the two large-bodied *Rheomys*.

**Figure 9:**
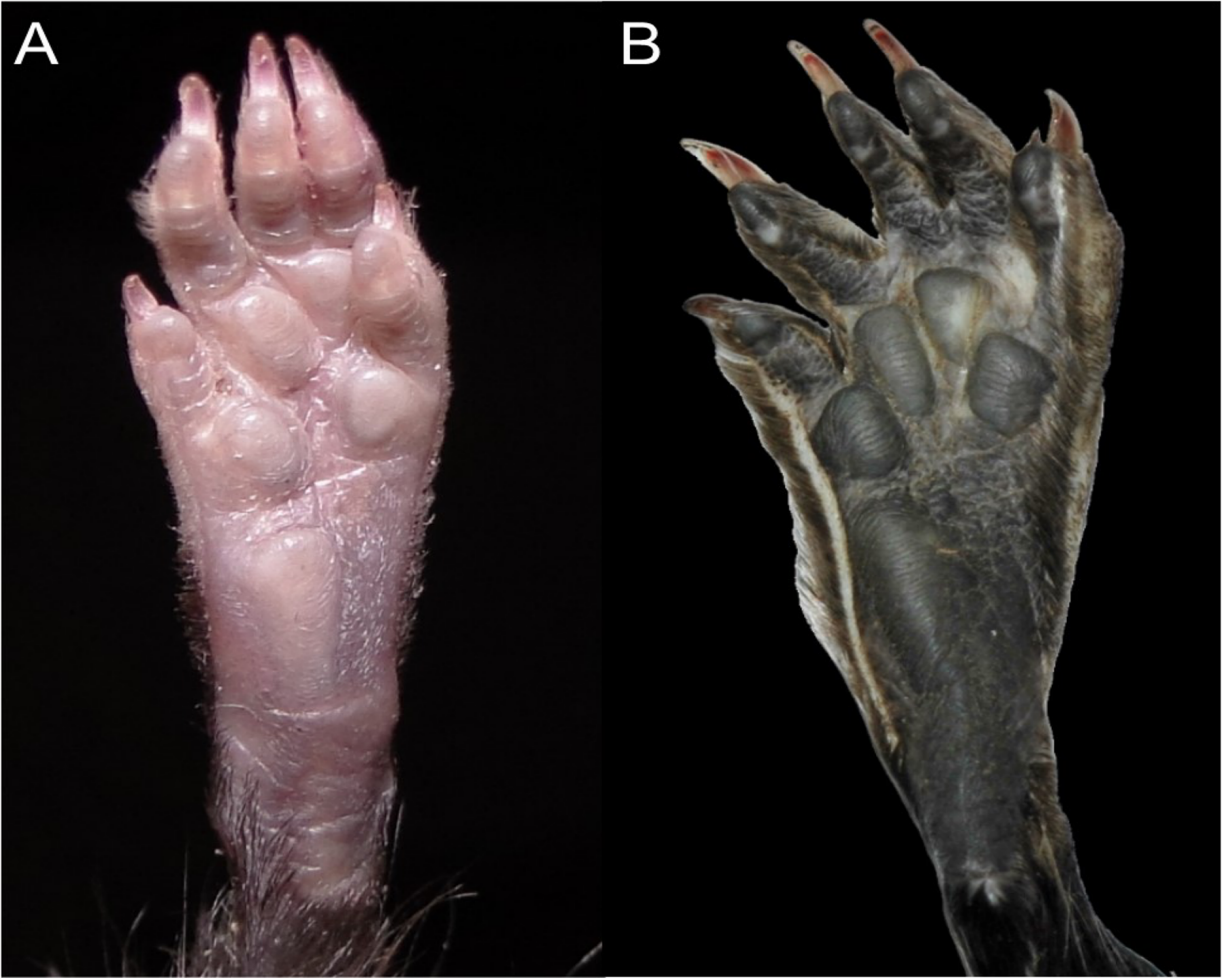
Plantar views of the left hind-feet in ichthyomyine rodents. The figure illustrates variations in the width of the hindfeet (character 13) in ichthyomyine rodents. (A) Narrow (*Neusticomys vossi*; MECN 4332); (B) broad (*Ichthyomys orientalis*; MECN 4914).

Character 13. Pes width: (0) narrow; (1) broad (Fig. 9). This character highlights, in a simplistic approach, one of the main external features of the ichthyomyines, the pes morphology (see Anthony, 1929; Voss, 1988). Narrow pes (longer than broader and with middle three toes longer than the two outside lateral ones), are less specialized for natation and characterize *Chibchanomys orcesi*, *Neusticomys*, and small-bodied *Rheomys*. In sharp contrast, broad and large hindfeet, well adapted for swimming, are found in *Anotomys leander*, *Chibchanomys* spp., *Ichthyomys*, and the two larger species of *Rheomys*.

**Figure 10:**
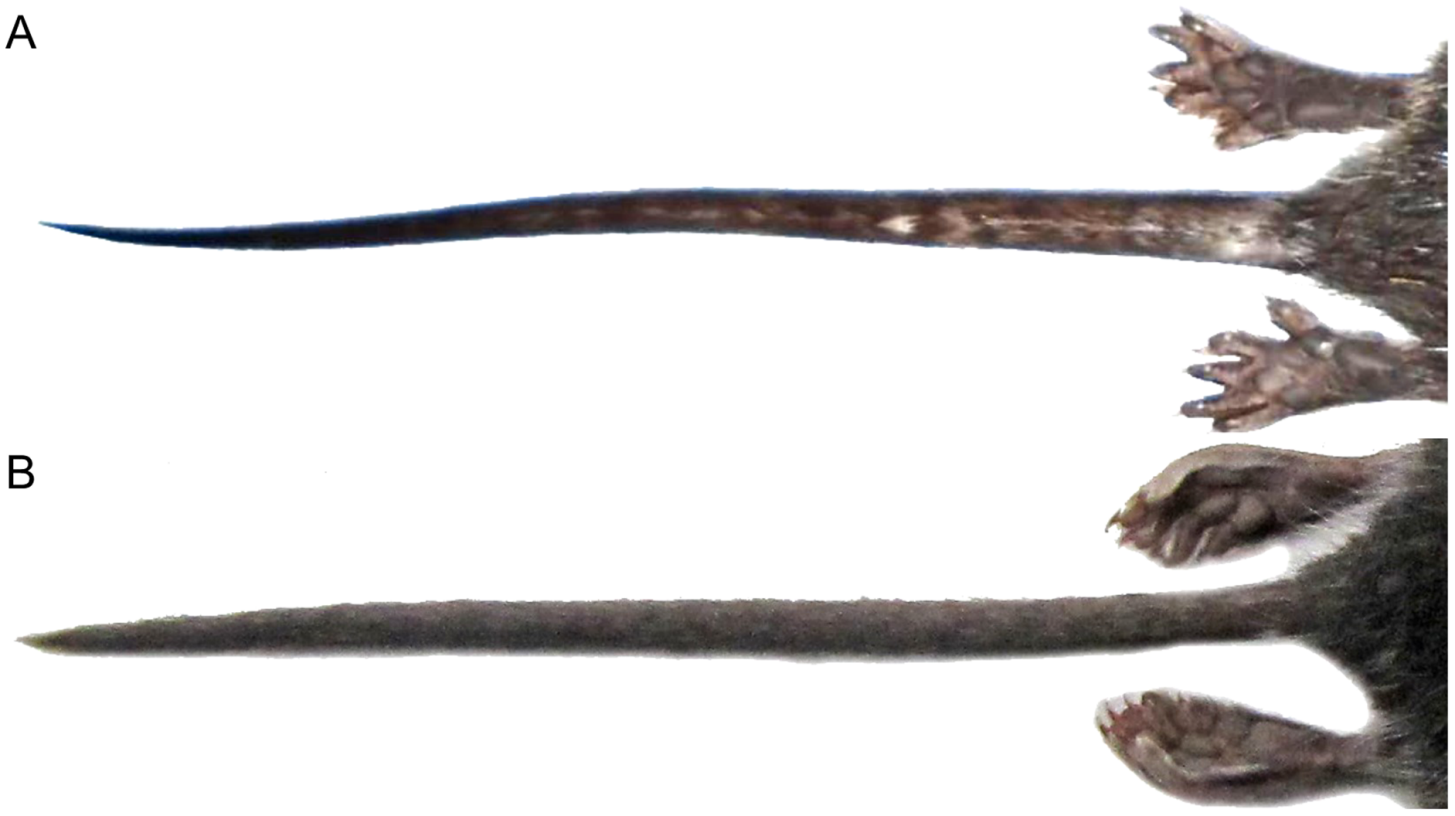
Dorsal views of the tail. The figure illustrates the general morphology of the tail (character 14) in ichthyomyine rodents. (A) Cylindrical or conical (*Ichthyomys orientalis*; MECN 4914); (B) compressed (*Chibchanomys* n. sp.; MUSA 13864).

Character 14. Tail morphology: (0) cylindrical or conical; (1) compressed laterally. This character highlights the different forms of the tail in mid cross-section (Fig. 10). Several ichthyomyines show approximately cylindrical tails in most part of its length (*e.g*., *Chibchanomys orcesi*, *Ichthyomys pittieri*) or the tail is thickened at the base and thinner towards the distal portion, describing an approximately conical shape (*e.g*., *Rheomys mexicanus*, *Rheomys underwoodi*, *Ichthyomys hydrobates*). Compressed tails, supposedly more adapted to swimming, are found in *Anotomys leander* and *Chibchanomys* spp.

Character 15. Tail ventral fringe: (0) absent; (1) present. In several ichthyomyines, tail hairs are longer on the ventral than on the dorsal surface, forming a hair fringe as a ventral extension of the tail (Voss, 1988). This condition is not present in *Ichthyomys tweedii*, *Anotomys leander*, species of *Neusticomys* and smaller species of *Rheomys*. *Chibchanomys orcesi* shows a slight ventral development of longer hairs, and we scored the species as (0). A developed ventral fringe characterizes several species of *Ichthyomys* (*e.g*., *Ichthyomys hydrobates*, *Ichthyomys stolzmanni*), other *Chibchanomys*, and acquires a noteworthy expression—resembling a continuous “keel” of stiff hairs-in larger *Rheomys*.

### Cranial morphology

**Figure 11:**
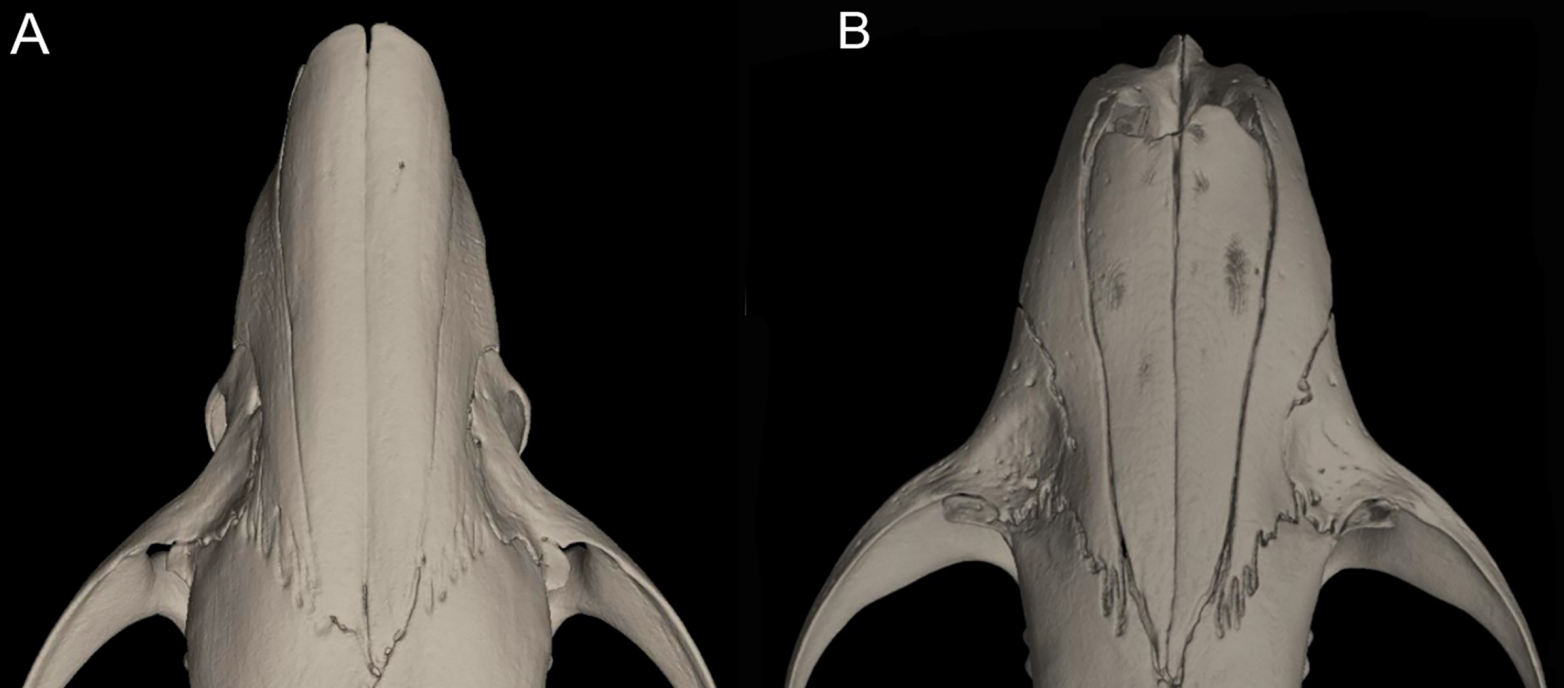
Detail of the morphology of the rostrum in ichthyomyine rodents. This figure illustrates variation in the general morphology of the rostrum (character 16), in ichthyomyine rodents. (A) Slender (*Neusticomys vossi*; MECN 4332); (B) broad (*Ichthyomys orientalis*; MECN 4914).

Character 16. Rostrum: (0) slender; (1) broad. This trait highlights the differences in the general morphology of the rostrum (Anthony, 1929). The rostrum, when evaluated from dorsal view, is slender—more pointed and clearly tapering forward—in *Anotomys leander*, *Chibchanomys*, high-Andean species of *Neusticomys*, and *Rheomys*. On the contrary, a heavy, broad and less tapering rostra characterizes species of *Ichthyomys* and the lowland species of *Neusticomys* (Fig. 11).

**Figure 12:**
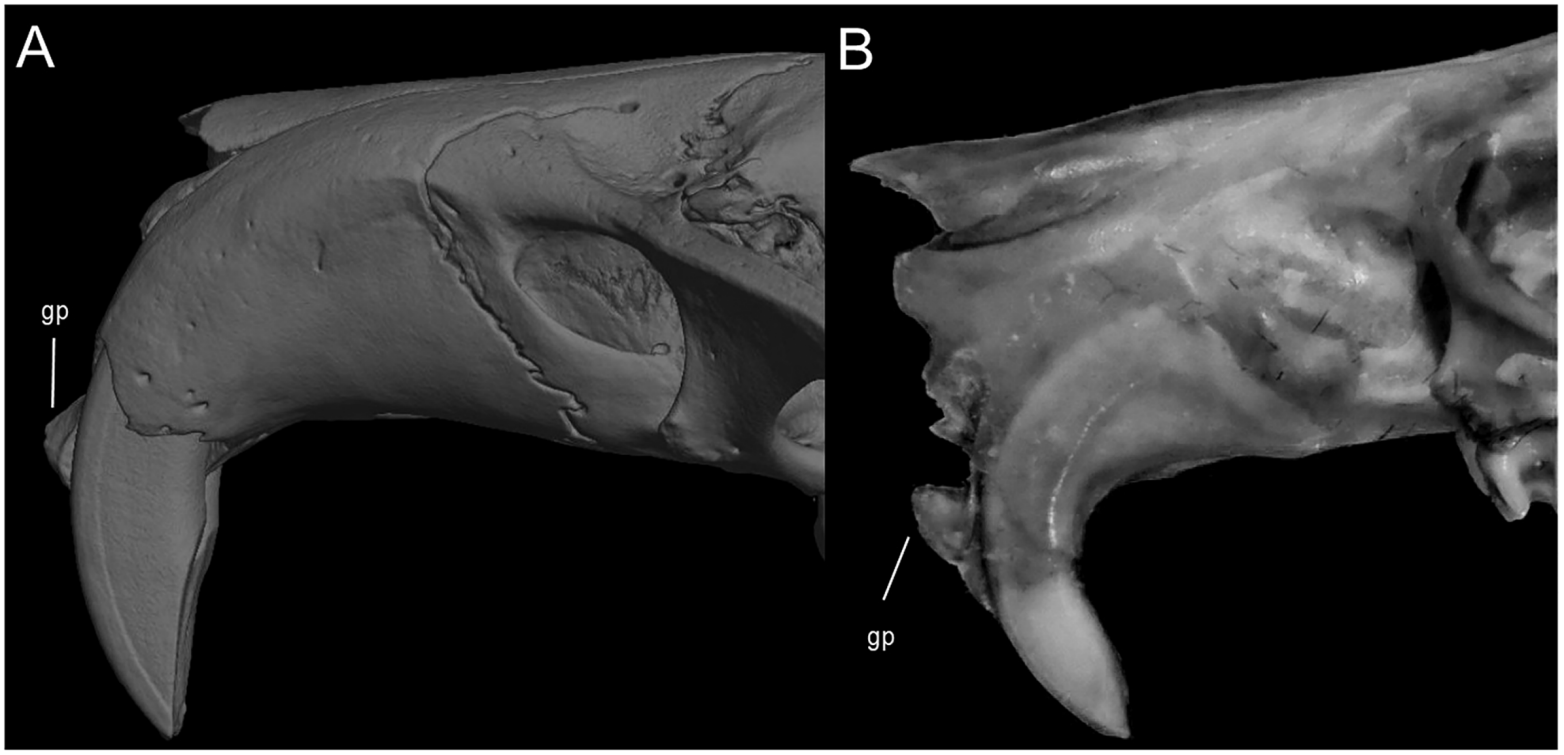
Differences in the development of the gnathic process in ichthyomyine rodents. Gnathic process, appearance in lateral view, in representative taxa of ichthyomyine rodents (character 17). (A) Small (*Ichthyomys orientalis*; MECN 4914); and (B) large (*Anotomys leander*; AMNH 244605).

Character 17. Gnathic process: (0) small; (1) large. The thin bony plate formed by the premaxillaries produced anteriorly, originating at the middle point of upper incisors, is named gnathic process (Voss, 1988: fig. 10) and is usually well-developed in all ichthyomyines (Fig. 12). However, perceptible differences are noticeable between genera, both in absolute and relative terms. Gnathic process is comparatively smaller—in comparison with the depth of the incisor—in *Chibchanomys orcesi*, species of *Ichthyomys*, and *Neusticomys*. Well-developed gnathic processes, in some cases including a dorsal continuity to the premaxillae above incisors (*e.g*., *R. mexicanus*) is exhibited by *Anotomys*, *Chibchanomys* spp., and *Rheomys*. Since this bony structure has been associated to the greater development of the mystacial vibrissae (through providing a surface for the origin of *M. nasolabialis profundis pars media inferior*; see Voss, 1988:288), a positive correlation between both traits is certainly expected. However, this correlation is not necessarily direct, as species of *Ichthyomys*, characterized by well-developed mystacial vibrissae, show inconspicuous gnathic processes. The rarity of developed gnathic process in non-ichthyomyine sigmodontines invites to a further exploration of this character. One of the few recorded exceptions is the akodontine genus *Bibimys*, that is easily recognizable by its larger gnathic process (Massoia, 1980); apparently, the structure has a presumptive connection with the bulbous, turgent, and pinkish large upper lips that this sigmodontine shows (Pardiñas, Galliari & Voglino, 2017).

Character 18. Nasal bones: (0) long; (1) short. The description and coding of this character follows Voss (1988, ch. 10). The state (1), when the anterior margins of nasals are short and the incisors and nasal openings are visible from above, is limited to *Ichthyomys*.

**Figure 13:**
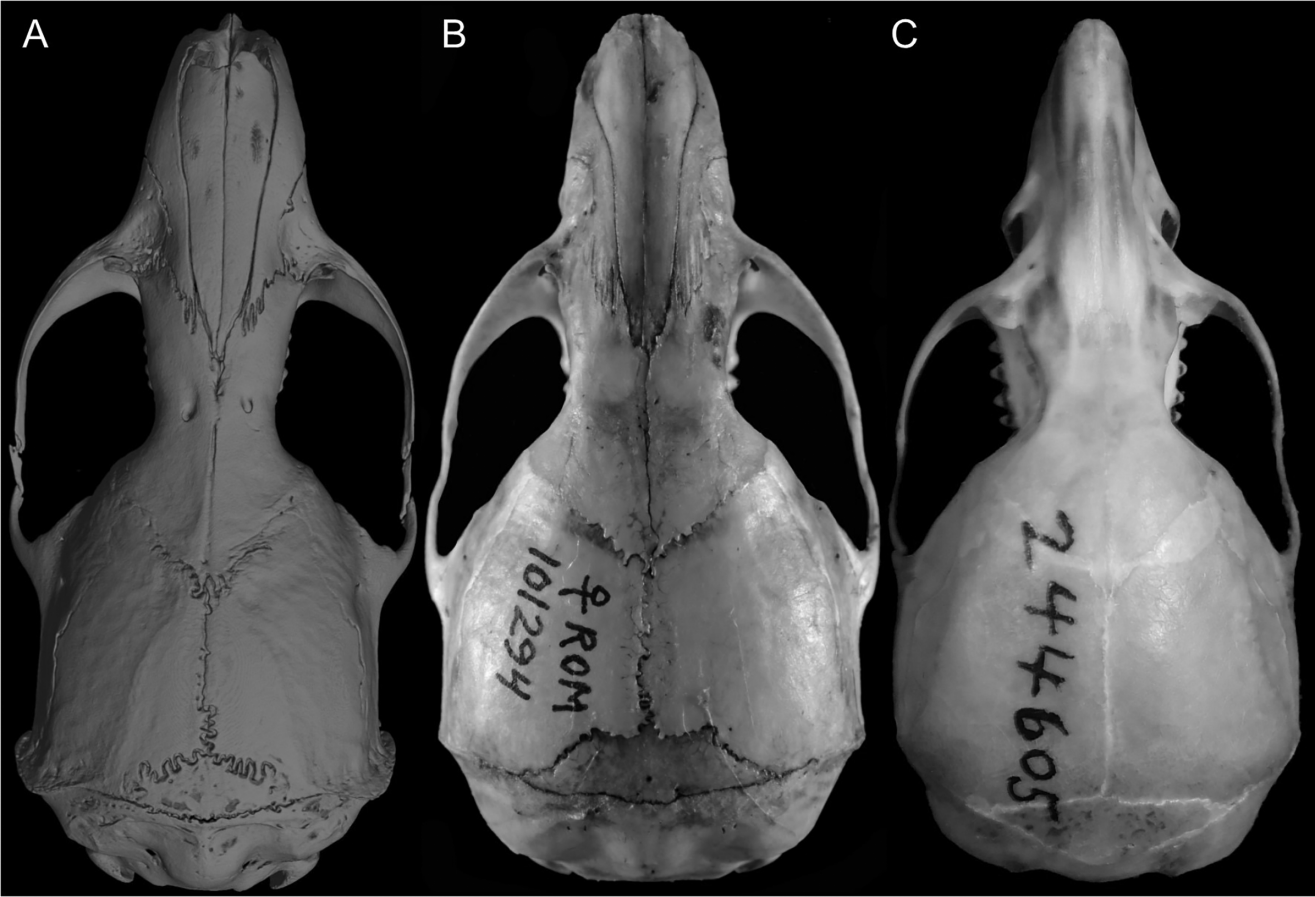
Dorsal views of adult crania. The figure highlights differences in the overall morphology of the braincase in ichthyomyine rodents (character 19). (A) Non-inflated (*Ichthyomys orientalis*; MECN 4914); (B) rounded (*Rheomys thomasi*; ROM 101294); (C) globular (*Anotomys leander*; AMNH 244605).

Character 19. Braincase form: (0) non-inflated; (1) rounded; (2) globular. This character contrasts braincase general morphology in dorsal view (Anthony, 1929). Non-inflated, “squared” braincases are found in *Ichthyomys*, and lowland species of *Neusticomys*. Rounded or slightly inflated braincases characterize *Chibchanomys orcesi*, high-Andean *Neusticomys* and *Rheomys*. Evidently globular braincases are restricted to *Anotomys* and the remainder *Chibchanomys*. See Fig. 13.

**Figure 14:**
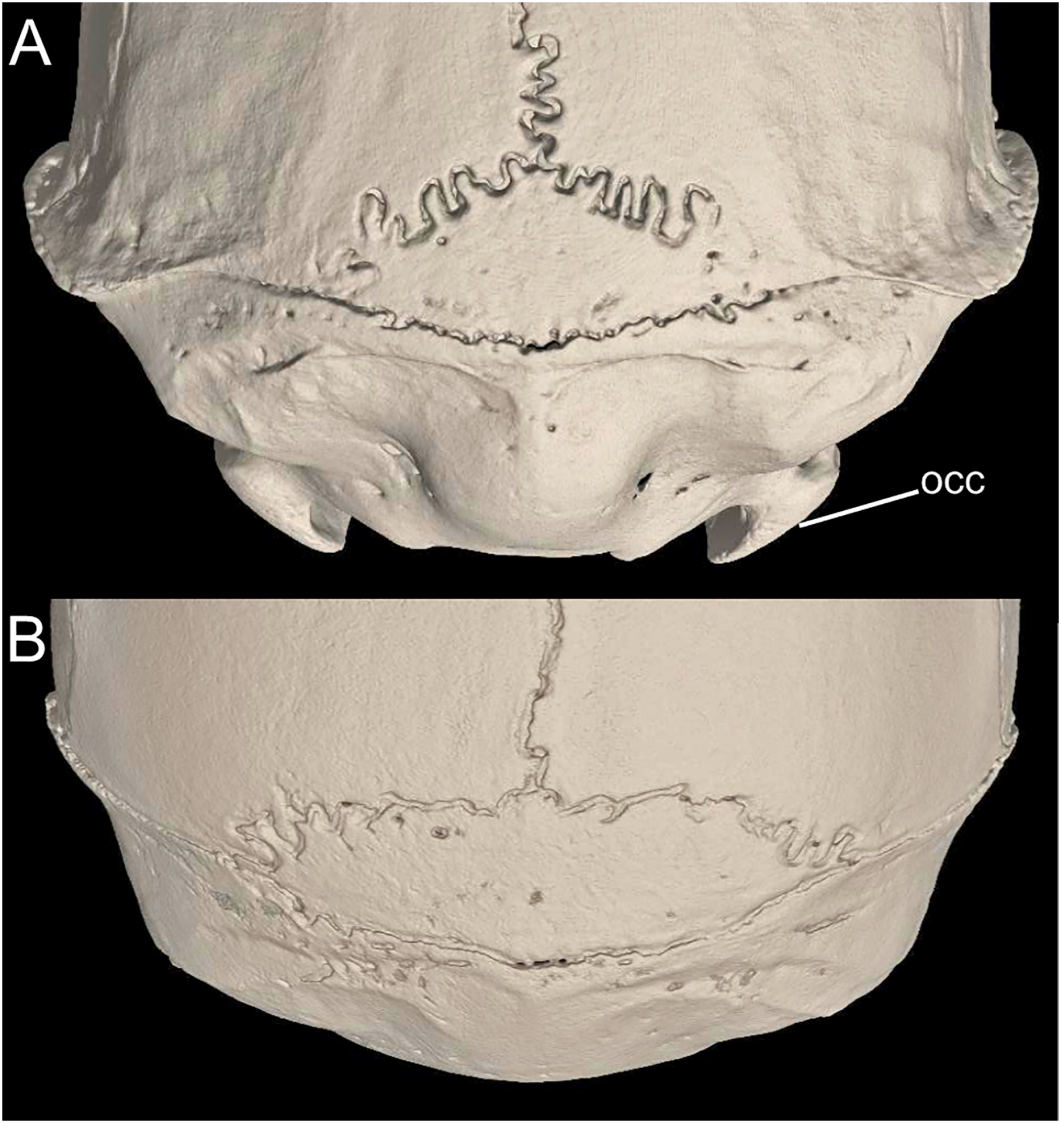
Dorsal views of the posterior braincase region. The figure illustrates variations in the position and development of the occipital condyles [occ] on the dorsal view of the braincase (character 20) in ichthyomyines. (A) Occ visible from above (*Ichthyomys orientalis*; MECN 4914); (B) occ not visible from above (*Neusticomys vossi*; MECN 4332).

Character 20. Development of occipital condyles: (0) visible from dorsal view; (1) not visible from dorsal view (Fig. 14). This character highlights the differential degree of development and backward exposure of occipital condyles when inspected from dorsal perspective. Well-developed condyles are found in *Ichthyomys* and lowland *Neusticomys*. Tiny, poorly developed or non-visible condyles are the condition recorded in the remaining ichthyomyines.

Character 21. Supraorbital foramina: (0) within the orbital fossae; (1) between the orbital fossae. The description and coding of this character follows Voss (1988, ch. 11); the state (1), is an autapomorphic trait of the genus *Ichthyomys*.

**Figure 15:**
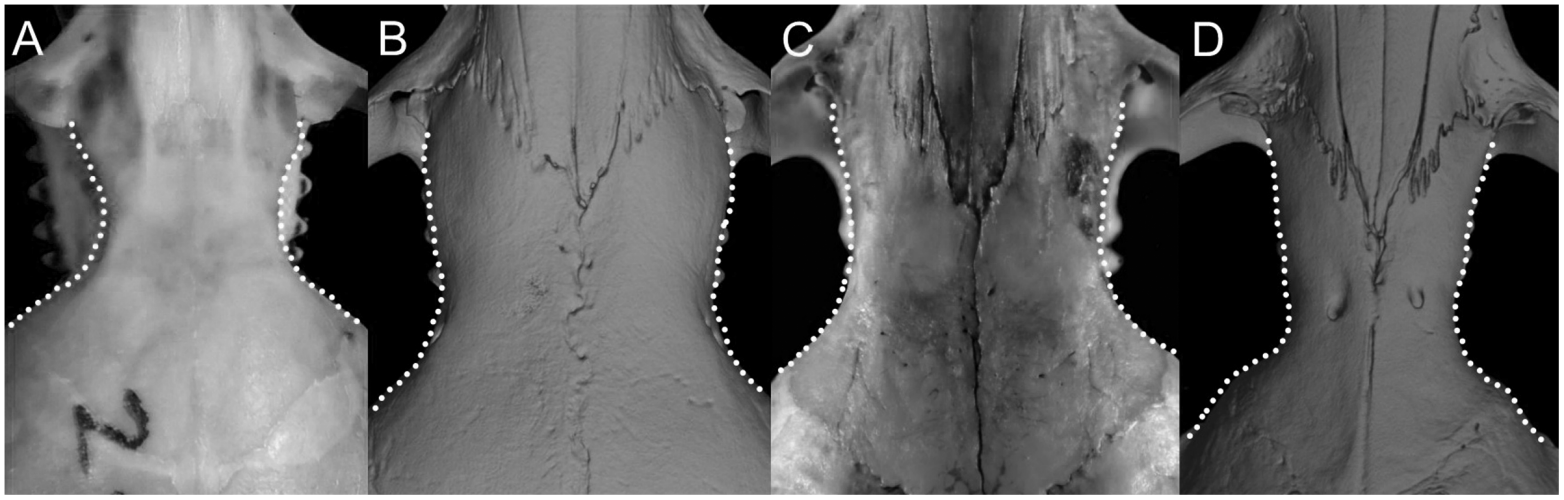
Details of the interorbital region. Shape and size of the interorbital region (appearance in dorsal view; lateral margins highlighted) in the Ichthyomyini (character 22). (A) Hourglass (*Anotomys leander*; AMNH 244605); (B) large (*Neusticomys vossi*; MECN 4332); (C) short (*Rheomys thomasi*; ROM 101294); and (D) very constrained (*Ichthyomys orientalis*; MECN 4914).

Character 22. Interorbital region: (0) hourglass; (1) large interorbital; (2) short interorbital; (3) very constrained (Fig. 15). Our characterization of the interorbit is probably a poor and overestimated attempt to assess the variation in this complex and age-changing region of the cranium (see Weksler, 2006). An interorbital region symmetrically constricted, the classical “hourglass” shape with rounded external borders, is recorded in *Anotomys*. An interorbital comparatively larger, with external margins more sharply defined, parallel or slightly constrained, is found in *Chibchanomys* and *Neusticomys*. The states (2) and (3) have correspondence with *Rheomys* and *Ichthyomys*, respectively, the former genus with interorbital region comparatively shortened, and the latter characterized by very constrained interorbital regions.

Character 23. Carotid arterial supply: (0) pattern 1; (1) pattern 2; (2) pattern 3. The description and coding of this character follows Voss (1988, ch. 12).

Character 24. Orbicular apophysis of malleus: (0) present; (1) absent. The description and coding of this character follows Voss (1988, ch. 13).

**Figure 16:**
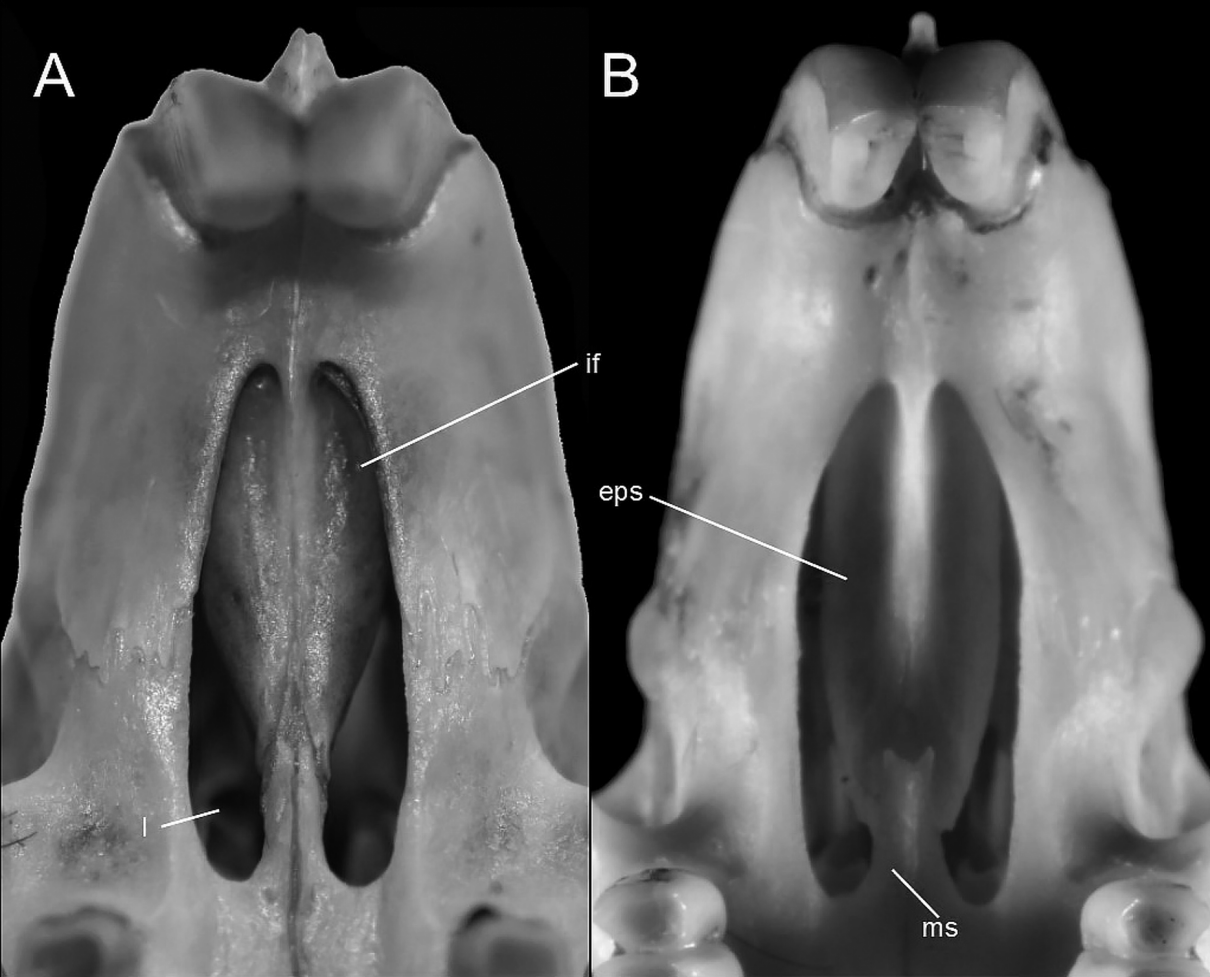
Ventral view of the diastemal palate. Notice the differential development of the premaxillary process [eps] of the incisive foramina [if] (character 25). (A) eps short (*Daptomys* sp.; JBM 2500); (B) large (*Rheomys thomasi*; ROM 101294). Other abbreviations: I, lumen, maxillary septum.

Character 25. Premaxillary process (“bony capsule”) of the incisive foramina: (0) short (covering <1/2 of the lumen of foramina); (1) large (>1/2 of the lumen). This character describes the differential expression of the premaxillary process of the incisive foramina (Fig. 16). Most previous authors recording traits associated with the interior portion of the incisive foramina concentrated on the maxillary septum (see the summary in Weksler, 2006:111; see also Teta et al., 2017, ch. 44, but incorrectly marked in fig. 4); however, see Patton & Hafner (1983*: 541*) or Patton (1987: figs. 13–20). All ichthyomyines have long incisive foramina, with posterior margins usually reaching the anterior margin of the M1 (see Voss, 1988*: 291*). We detected several forms showing the bony ampulla of the premaxillary process occupying less than a half of the lumen of the incisive foramina (*e.g*., *Anotomys*, *Chibchanomys orcesi*, *Neusticomys monticolus*, *Neusticomys vossi*); the other state is recorded in the remaining species of *Chibchanomys*, most of the *Ichthyomys*, several *Neusticomys* and *Rheomys*. *Ichthyomys pittieri* and *I. stolzmanni* are polymorphic for this character.

**Figure 17:**
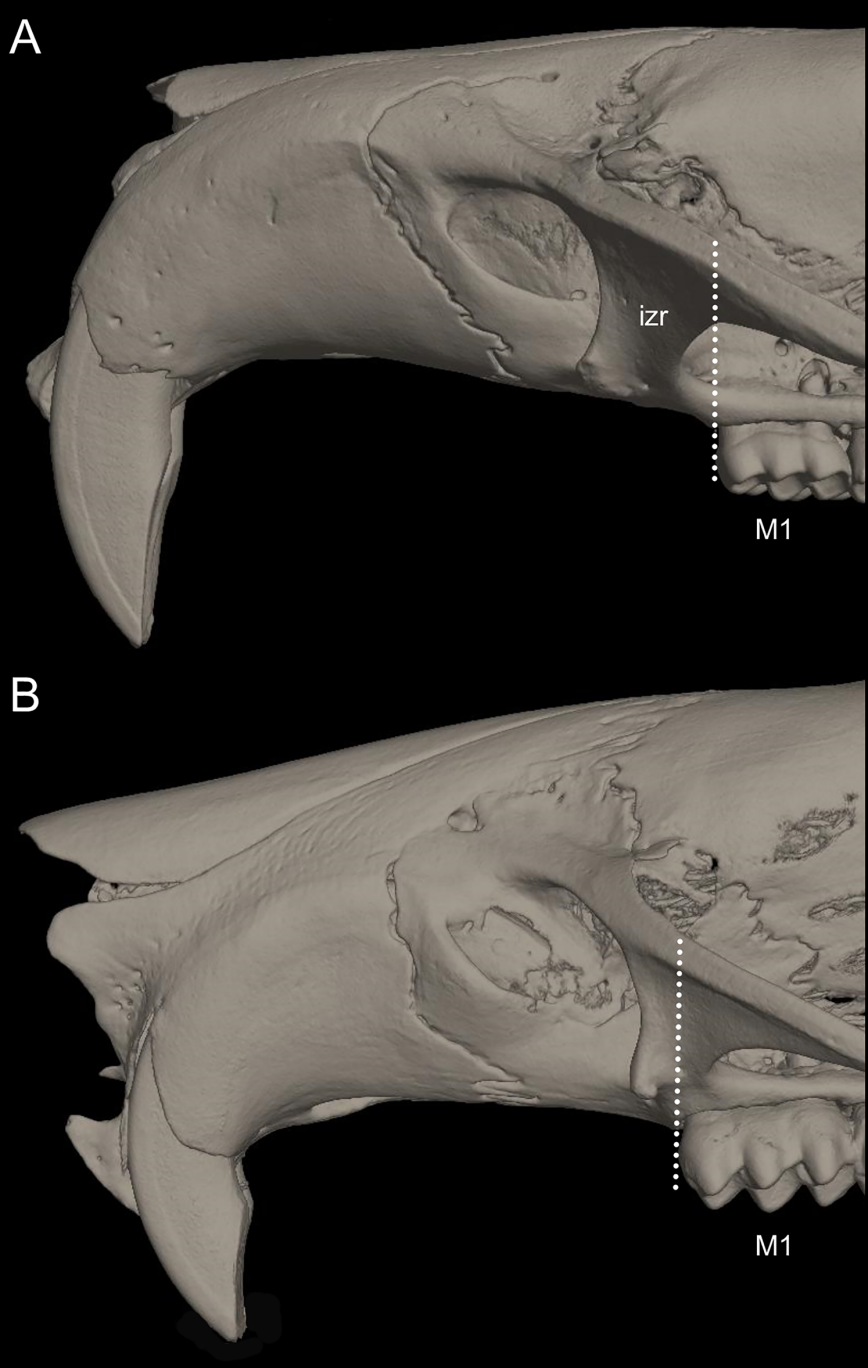
Lateral views of the rostral and zygomatic regions. Lateral views of the rostral and zygomatic regions Illustrating the variations in the position of the posterior margin of the zygomatic plate [izr] relative to the anterior face (marked with a dotted line) of the first upper molar [M1] (character 26). (A) Anterior (*Ichthyomys orientalis*; MECN 4914); (B) even with or posterior (*Neusticomys vossi*; MECN 4332).

Character 26. Position of the posterior margin of the zygomatic plate relative to the alveolus of the M1: (0) anterior; (1) even with or posterior. The description and coding of this character follows Weksler (2006, ch. 29). Some perceptible degree of age-related variation was detected during the scoring (Fig. 17).

**Figure 18:**
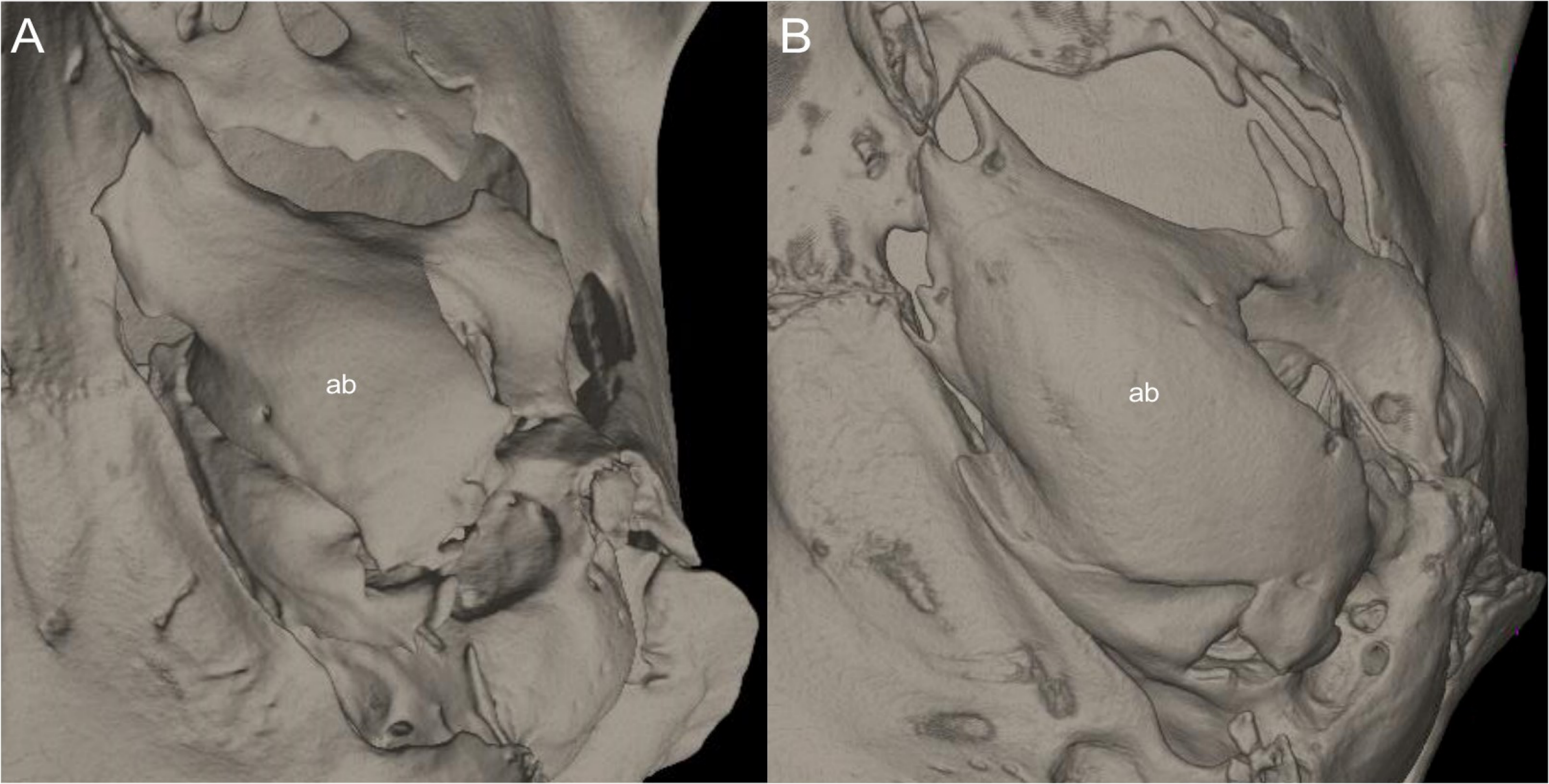
Details of the auditory bullae. Left otic capsule of ichthyomyine rodents illustrating variations in the morphology of the auditory bullae [ab] morphology (character 27). (A) Flask-shaped, not inflated (*Ichthyomys orientalis*; MECN 4914); (B) globular (*Neusticomys vossi*; MECN 4332).

Character 27. Auditory bullae general morphology: (0) flask-shaped, not inflated; (1) globular. The perception and characterization of the shape of the otic capsules, mostly driven by the development of the ectotympanic, is hard and subjective (see for example, Steppan, 1995; Pacheco, 2003). We interpreted as non-inflated, flask-shaped auditory bullae those exhibited by some *Ichthyomys* (*e.g*., *I. hydrobates*, *I. tweedii*) and the largest *Rheomys* (*R. mexicanus*). Other ichthyomyines with available material were coded as (1). See Fig. 18.

**Figure 19:**
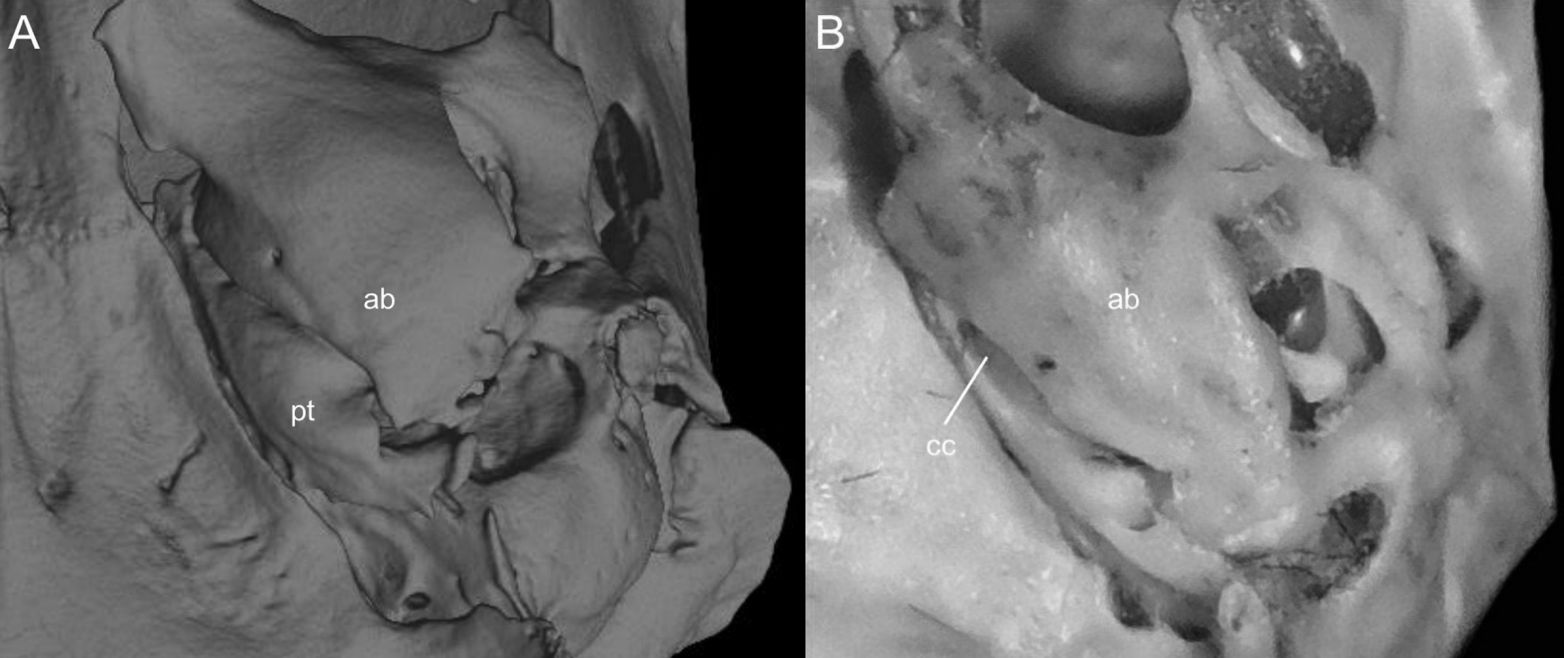
Detail of the left otic capsule. Ventral views of the left auditory bulla illustrating variations in the morphology of the petrotympanic (character 28). (A) Largely exposed (*Ichthyomys orientalis*; MECN 4914); (B) covered by the ectotympanic (*Anotomys leander*; AMNH 244605). Other abbreviations: ab, auditory bulla; cc, carotid canal.

Character 28. Petrotympanic: (0) largely exposed; (1) covered by the ectotympanic (especially near the carotid canal) (Fig. 19). Although the petrosal portion of the otic capsule is always exposed in the ichthyomyines (see Voss, 1988: 298), some discontinuous variation is detected in relation to the degree of exposition and its perception when the region is examined from above. Most of the surveyed forms exhibit the state (1); exceptions are species of *Chibchanomys* (excluding *C. orcesi*), *Ichthyomys orientalis*, and the high Andean forms of *Neusticomys* (*N. monticolus*, *N. mussoi*, and *N. vossi*). This trait is obviously linked with the development of the ectotympanic portion of the auditory bulla, although the correlation is not strictly direct. For an alternative view in the evaluation of characters 28 and 29, see Weksler (2006, ch. 41).

**Figure 20:**
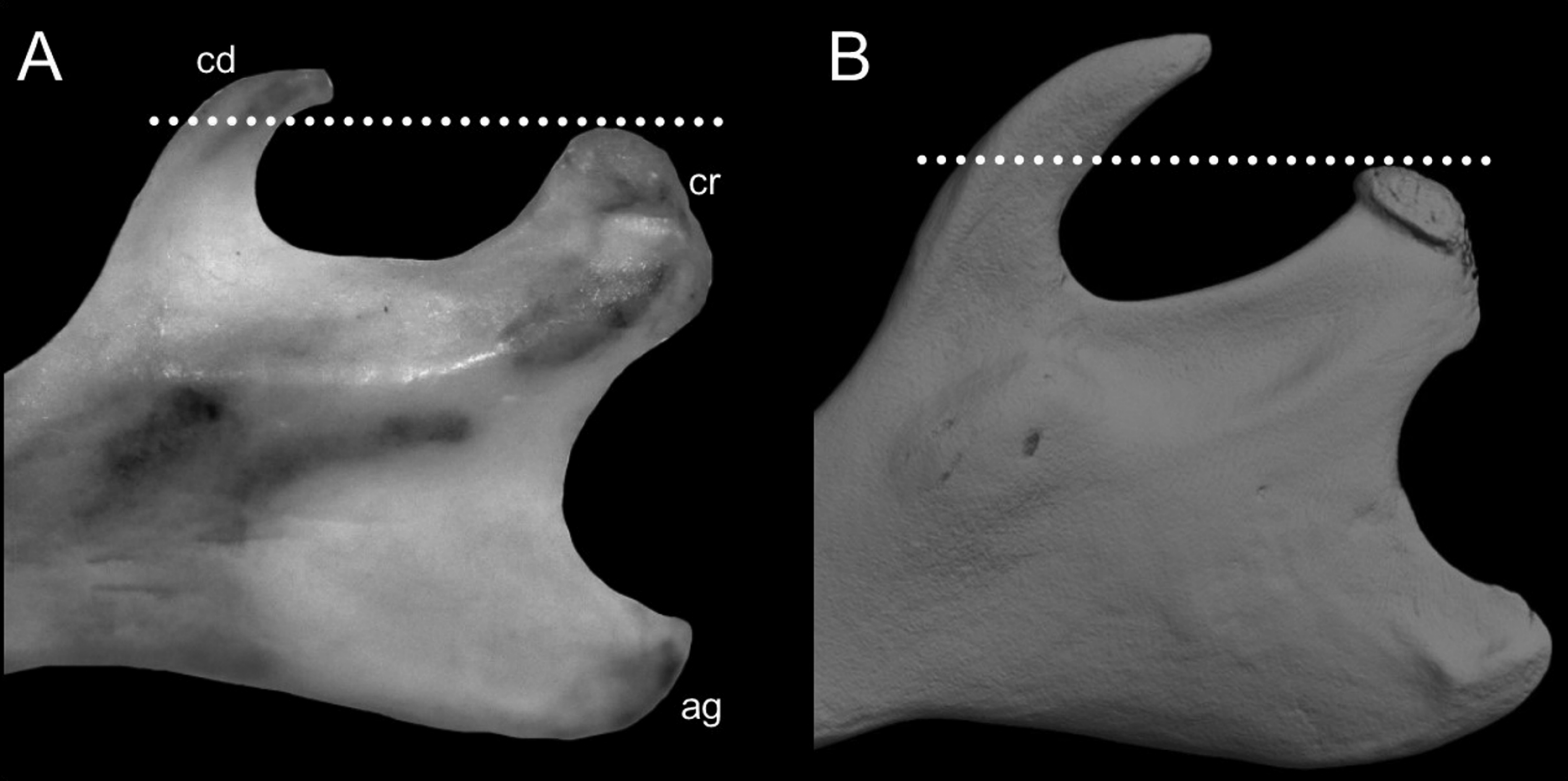
Labial views of the left mandible (vertical ramus). The figure shows the variation in the height of the coronoid process [cd] relative to the height of the condyle [cr] in the Ichthyomyini (character 29). (A) More or less at the same level (*Chibchanomys* n. sp.; MUSA 13864); and (B) cd clearly surpassing the cr (*Ichthyomys orientalis*; MECN 4914). Other abbreviation: ag, angular process.

Character 29. Coronoid process relative height to condyle (dentary): (0) more or less at the same level; (1) coronoid clearly surpassing the condyle. According to Voss (1988:299), “in all ichthyomyines the coronoid process is well developed and falciform,” highlighting the role of this structure in the anchorage of important masticatory muscles (Voss, 1988:393). To our understanding, a discrete variation of the coronoid process can be characterized (Fig. 20), contrasting the long, sword-shaped coronoids which clearly surpass the height of the condyle in several *Ichthyomys*, *Neusticomys*, and *Rheomys vs*. the more hook-shaped and delicate structures present in *Anotomys*, *Chibchanomys*, and *R. thomasi*.

### Dental morphology

Character 30. Upper incisors: (0) delicate; (1) heavy. Important differences among ichthyomyines have been highlighted regarding incisor general development and robustness (Voss, 1988:403–404). Comparatively narrow and delicate incisors are detected in *Anotomys*, *Chibchanomys*, some *Neusticomys* (*N. monticolus*, *N. vossi*, and *N. mussoi*), and *Rheomys*. Heavy incisors, broader and deeper, are recorded in *Ichthyomys* and the remaining species of *Neusticomys*.

**Figure 21:**
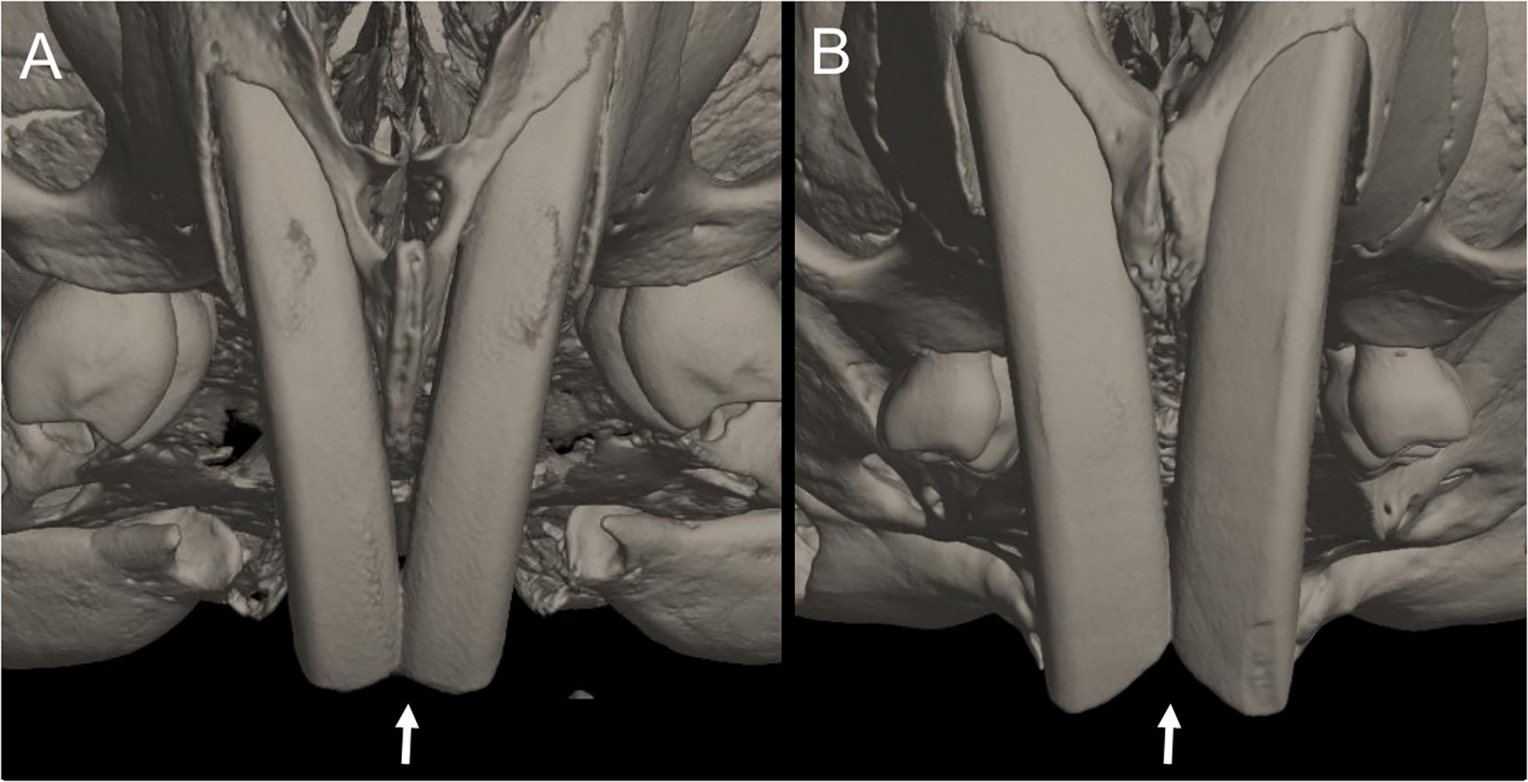
Details of the cutting edge of the upper incisors. Close up of the frontal view of upper incisors in ichthyomyine rodents highlighting the variation in the shape of the cutting edge (character 31). (A) Shallow inverted “v” (*Neusticomys vossi*; MECN 4332). (B) Deep inverted “v” (*Ichthyomys orientalis*; MECN 4914).

Character 31. Shape of cutting edge of upper incisors as inverted “v:” (0) shallow; (1) deep. Main differences of the incisive morphology when judged in frontal view were detailed described by Voss (1988:377). All ichthyomyines show the cutting edge of the upper incisors in inverted v-shaped, as a possible adaptation to grab slippery preys (Fig. 21). The survey reveals that the state (1) is autapomorphic of *Ichthyomys*.

**Figure 22:**
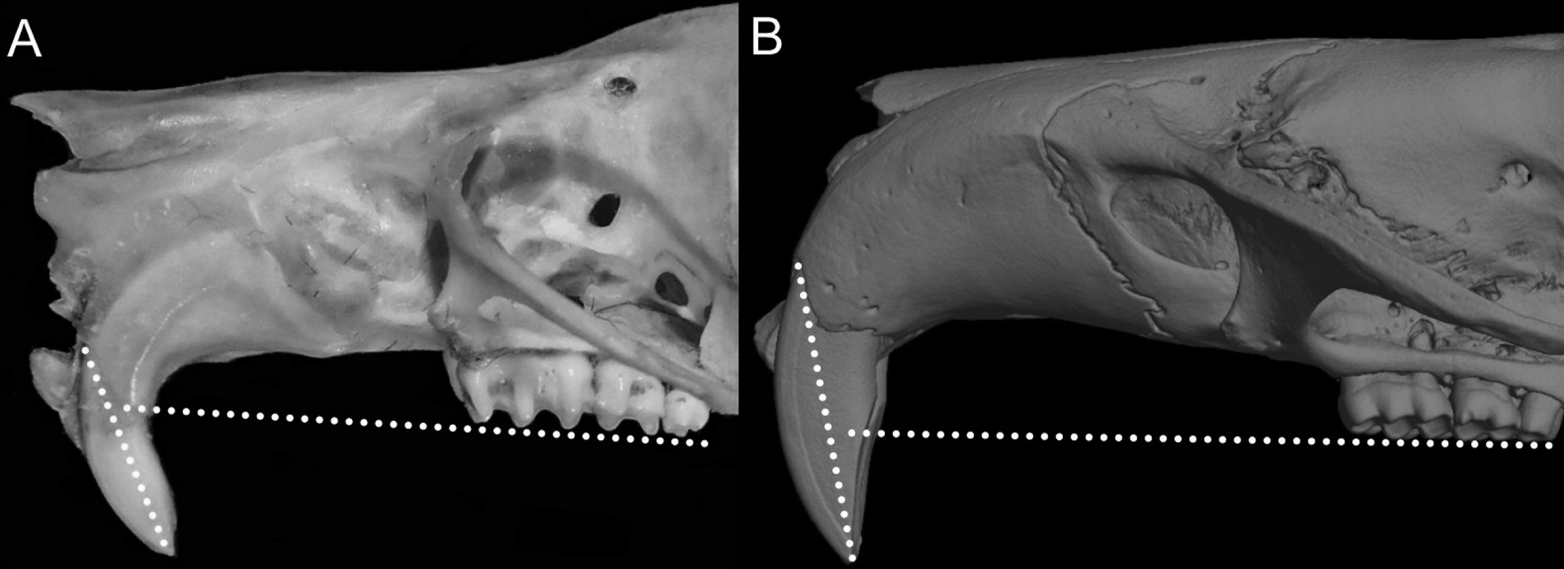
Lateral view of the rostral region. Left lateral view of the rostrum in ichthyomyine rodents highlighting the variation in incisor procumbency (character 32). (A) Opisthodon (*Anotomys leander*; AMNH 244605); and (B) tending to orthodont (*Ichthyomys orientalis*; MECN 4914). Dotted lines conform to the incisive index of Thomas (1919).

Character 32. Incisors procumbence: (0) opisthodont; (1) tending to orthodont. In describing the tribe, Voss (1988:319) stated that the incisors are “opisthodont but approaching the orthodont condition.” The present character is an attempt to coarsely differentiate both conditions (Fig. 22), after measuring the angle in several individuals, according to Thomas (1919). More clearly opisthodont forms are *Anotomys*, *Chibchanomys* (excluding *C. orcesi*), *I. pittieri*, *N. monticolus*, *N. oyapocki*, and *Rheomys*. The remainder surveyed ichthyomyines tends to orthodonty.

**Figure 23:**
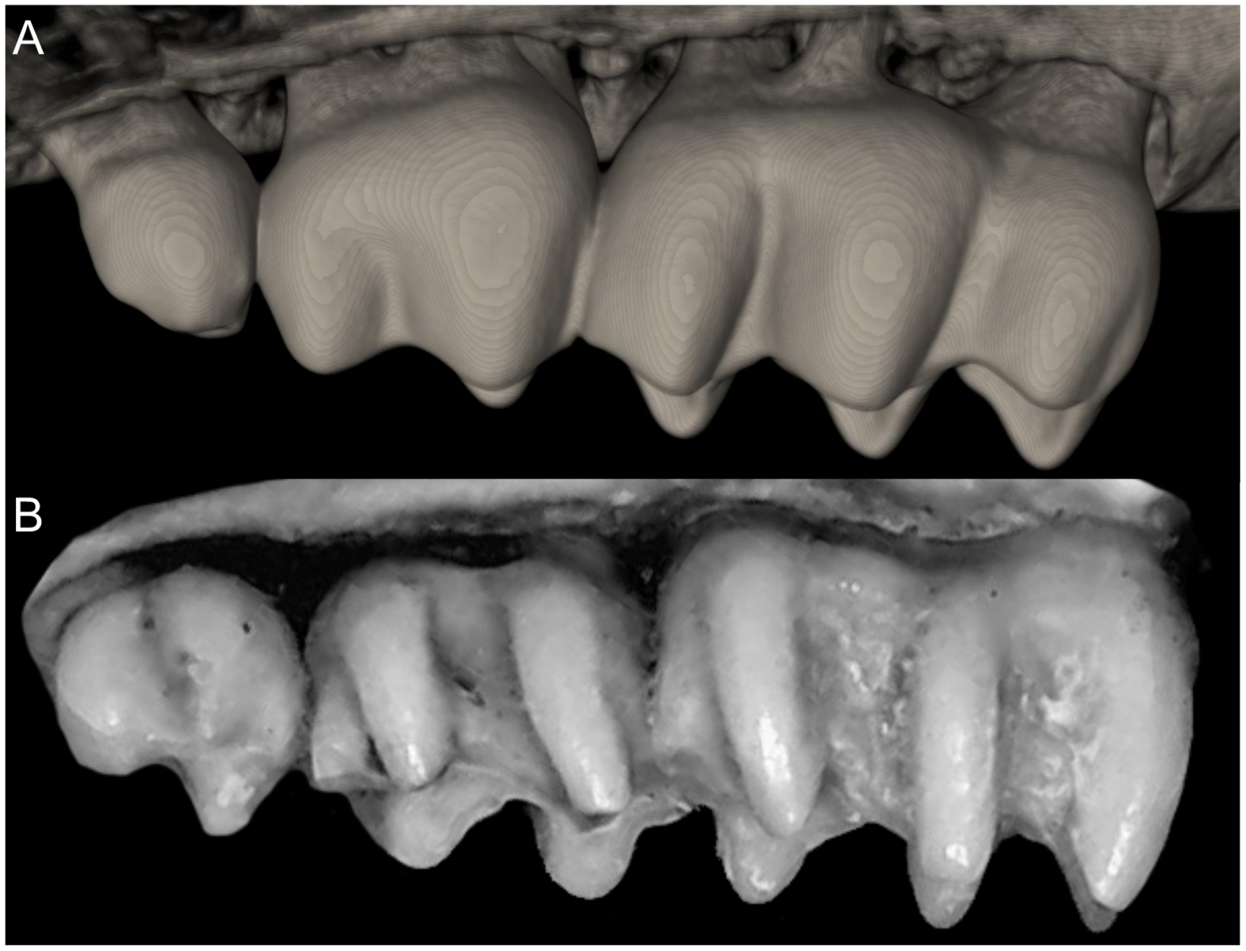
Detail of the upper molar row (lateral view). Figure highlights the variation in tubercular hypsodonty in the Ichthyomyini (character 33). (A) Moderate (*Neusticomys vossi*; MECN 4332); (B) marked (*Anotomys leander*; AMNH 244605).

Character 33. Tubercular hypsodonty: (0) moderate; (1) marked. Ichthyomyines were sorted in two main groups (but also recognizing the existence of intermediate forms) regarding the height of the dental cusps in unworn teeth (Voss, 1988: 283), ranging from those with tall and sharp (*Anotomys*, *C. trichotis*, *R. mexicanus*, and *R. underwoodi*) to low and rounded cusps (*Ichthyomys* and *Neusticomys*). The observed variation was correlated with functional variables (Voss, 1988: 378). Here, we adopted a reductive approach and restricted the state (1), implying really high-crowned molars, to describe the condition observed in *Anotomys* and *R. mexicanus*; the remainder ichthyomyines are scored as state (0), while *Chibchanomys trichotis* is regarded as indeterminate for this character (Fig. 23).

**Figure 24:**
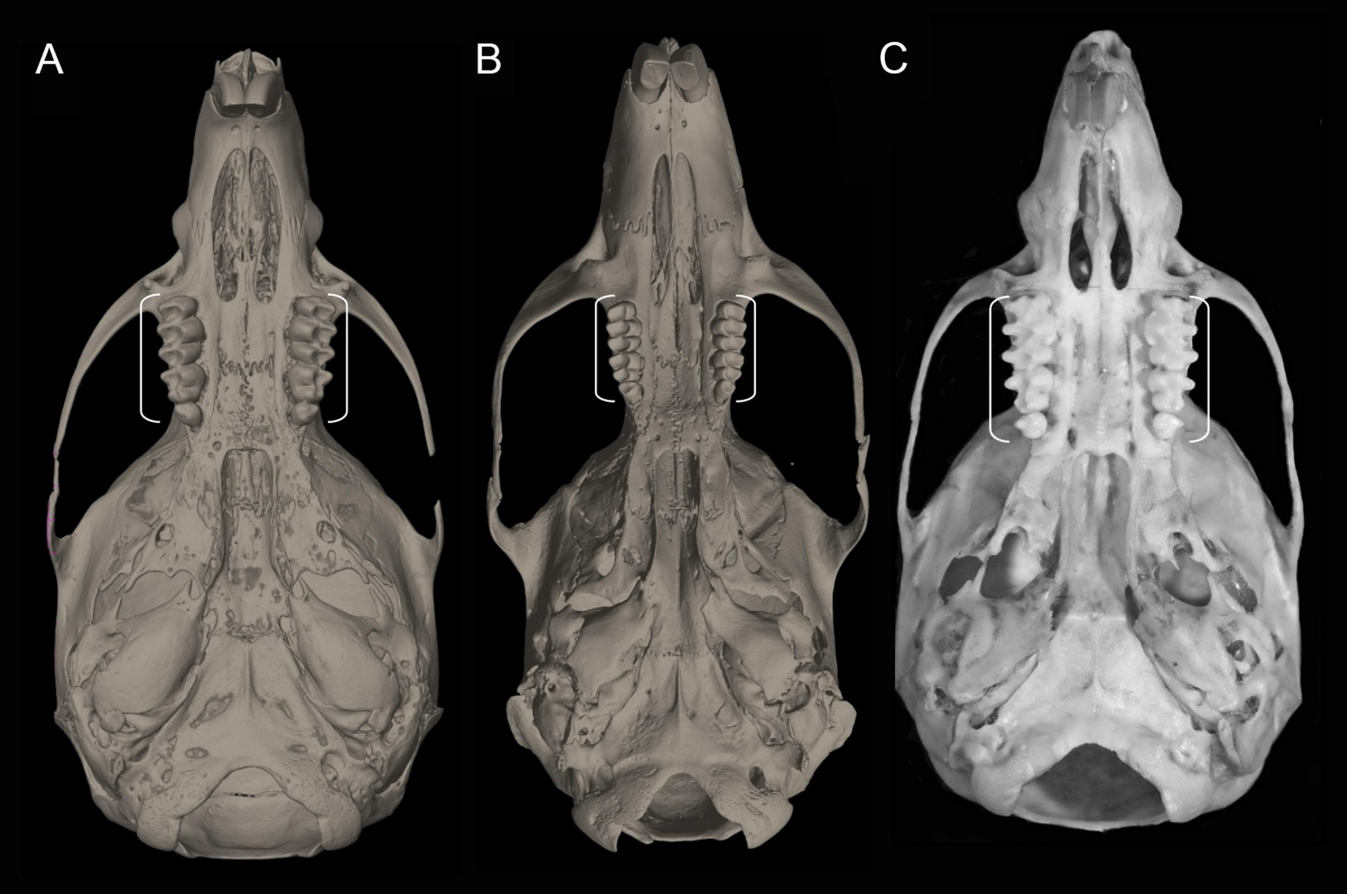
Ventral view of adult crania. Figure illustrates variations in the ratio of upper toothrow length to the length of the adult skull in ichthyomyine rodents (character 34). (A) Normal (*Neusticomys vossi*; MECN 4332); (B) “reduced” (*Ichthyomys orientalis*; MECN 4914); (C) “enlarged” (*Anotomys leander*; AMNH 244605). Please see Table 3 for details.

Character 34. Molars comparative development relative to skull: (0) normal; (1) reduced; (2) enlarged (see, Fig. 24). This character constitutes a clearly subjective appreciation but includes concepts (micro- and macrodonty) developed quantitatively by Schmidt-Kittler (2006). We employed a ratio of the length of molars/condyle-basal length as an index of this trait (Table S3). The “disparate” size of the molars relative to the skull (state 2) in *Anotomys* was remarked as unique within the tribe (Voss, 1988). We coded in this same state most of the species of *Rheomys* (except for *R. thomasi*). “Reduced” molars (state 1) were recorded for *Ichthyomys* (Voss, 1988: 382); here we also coded in this category the members of *Chibchanomys* and the lowland species of *Neusticomys*. The remaining ichthyomyines are treated here as state (0).

**Figure 25:**
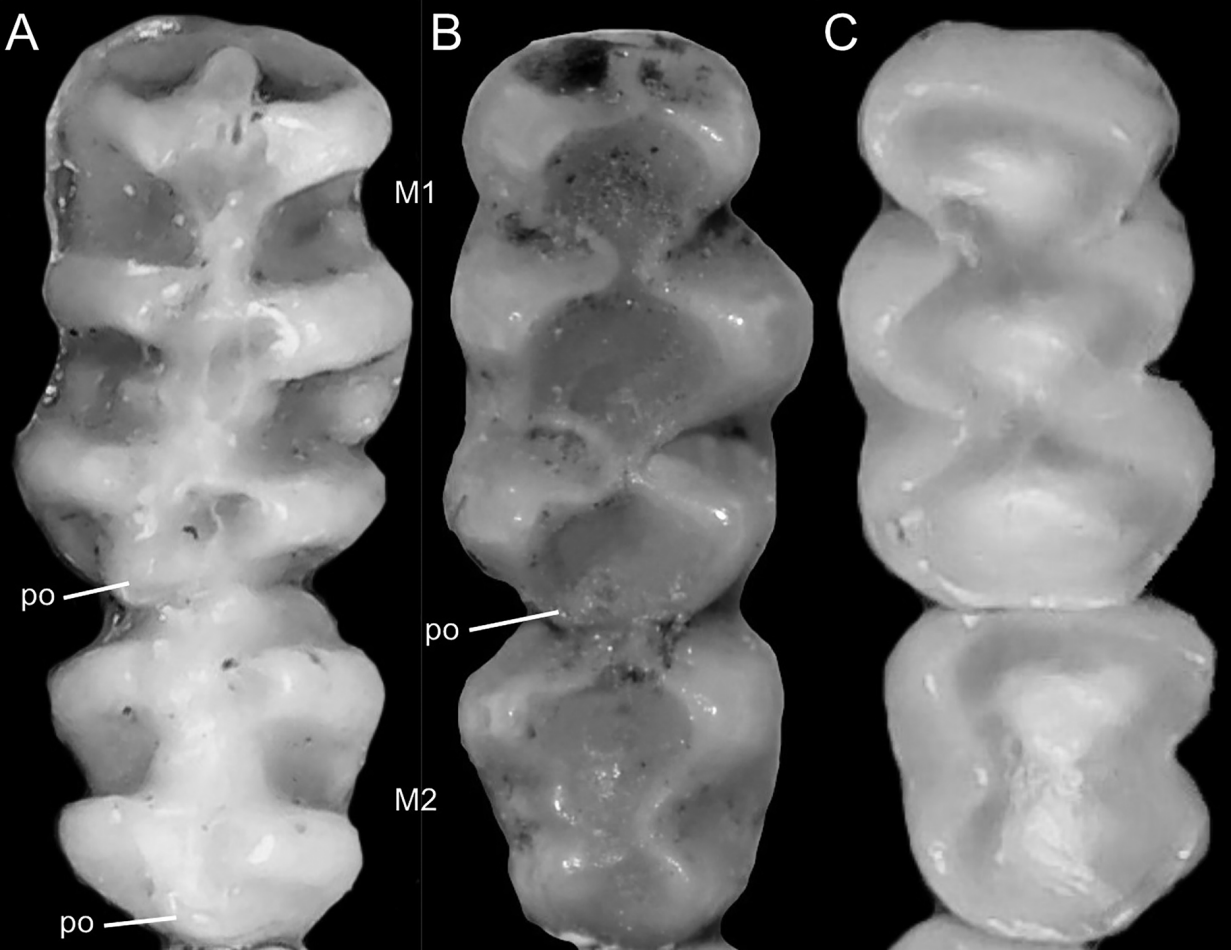
Details of the first two upper molars. Occlusal view of right M1-M2 illustrating variations in the presence of the posteroloph [po] in the upper molars of ichthyomine rodents (character 35). (A) po present in M1-M2 (*Anotomys leander*; AMNH 244605); (B) po present in M1 (*Neusticomys vossi*; MECN 4332); (C) po absent in M1-M2 (*Rheomys thomasi*; ROM 101294).

Character 35. Posteroloph in upper molars: (0) in M1 and M2; (1) in M1 only; (2) absent in M1 and M2. In the diagnosis of the tribe, Voss (1988:319) stated “mesoloph(id)s, anteroloph(id)s, posteroloph(id)s, and other accessory enameled structures of the molar crowns small and inconspicuous or absent.” We characterized the record of posterolophs in both first and second upper molars, trying to evaluate the condition in adult individuals (Fig. 25). Although our survey is clearly diminished by the availability of specimens, *Anotomys*, *Chibchanomys trichotis*, *Neusticomys monticolus*, and *Rheomys mexicanus* were scored as state (0), while other species of *Neusticomys* were treated as state (1). In the remainder ichthyomyines we considered the posteroloph as absent (2).

**Figure 26:**
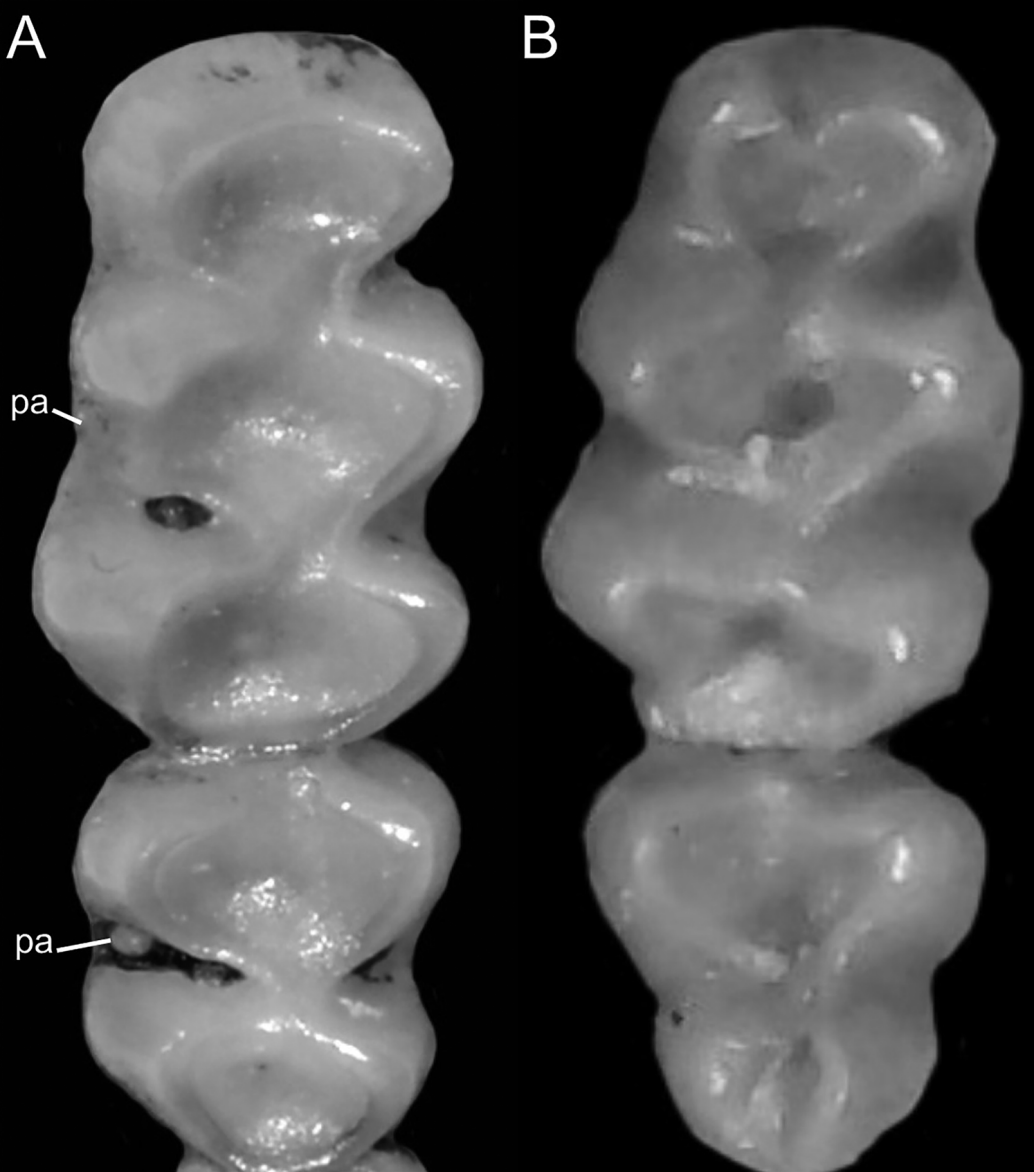
Detail of the occlusal view of the right M1-M2. Occlusal view of right M1-M2 illustrating variation in the presence of the paralophule [pa] in ichthyomine rodents (character 36). (A) Present (*Neusticomys vossi* ; MECN 5027); (B) absent (*Daptomys oyapocki*; AMNH 267597).

Character 36. Paralophule in M1-M2: (0) present; (1) absent. Paralophule is an accessory structure typically overlooked (*e.g*., not mentioned in Voss, 1988; but described as present in *Neusticomys ferreirai*, Percequillo, Carmignotto & Silva, 2005) and usually fused with the mesoloph in adults of several species of sigmodontines (Fig. 26). However, its independent nature and labial location, at the base of the paracone, can be confidently determined (Barbière et al., 2019). We detected minute paralophules in the M1-M2 of *Neusticomys monticolus*, *Neusticomys vossi*, *Neusticomys ferreirai*, *Rheomys mexicanus*, and *Rheomys raptor*.

**Figure 27:**
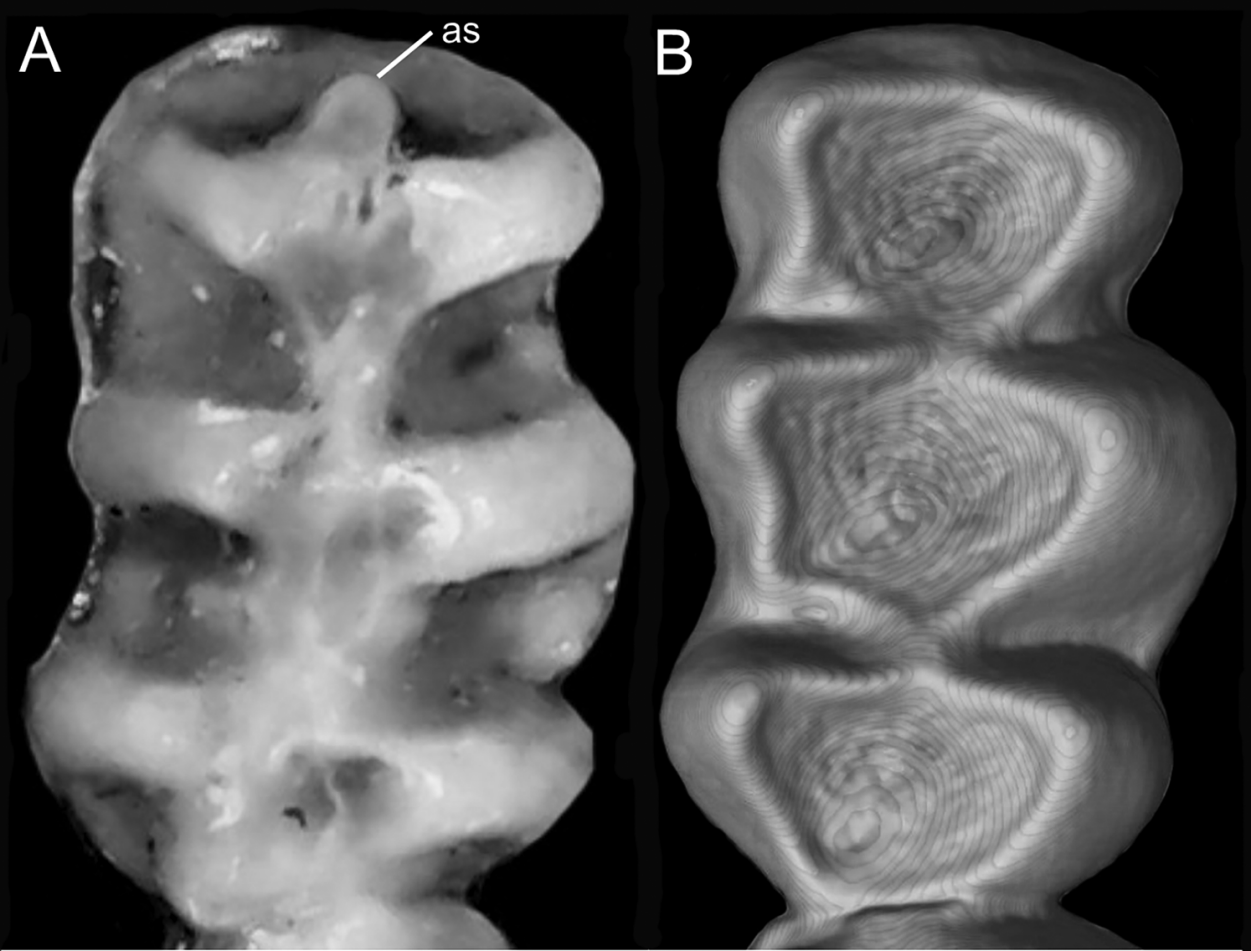
Details of the right first upper molar. The figure (right M1 in occlusal view) highlights the variation in the presence of the anteromedian style [as] in M1 (character 37) in ichthyomyine rodents. (A) Present (*Anotomys leander*; AMNH 244605); (B) absent (*Neusticomys vossi*; MECN 4332).

Character 37. Anteromedian style in M1: (0) present; (1) absent (Fig. 27). We detected this rare accessory cusp (see Barbière et al., 2019 for a discussion about its presumed origin) in two taxa (*Anotomys*, *Rheomys mexicanus*). Although usually small in the few sigmodontines that exhibits the anteromedian style, it acquires a noticeable expression in *Anotomys*.

**Figure 28:**
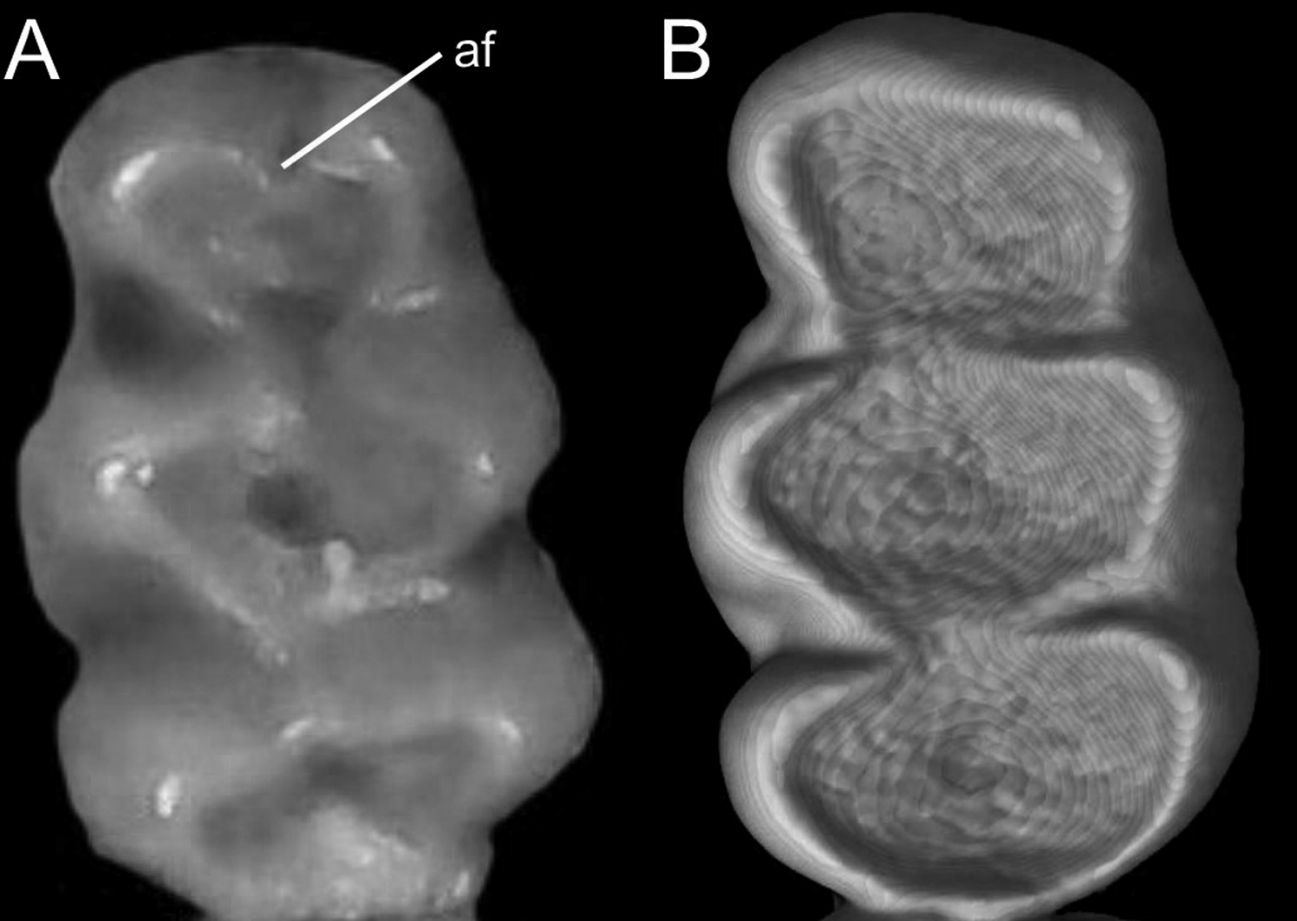
Details of the M1. The figure (left M1 in occlusal view) illustrates the variation in the presence of the anteromedian flexus [af] in M1 (character 38) in the Ichthyomyini: (A) present (*Daptomys oyapocki*; AMNH 267597) and (B) absent (*Ichthyomys orientalis*; MECN 4914).

Character 38. Anteromedian flexus on M1: (0) present; (1) absent (Fig. 28). The anteromedian flexus is barely present in ichthyomyines and, when present, always inconspicuous (Voss, 1988: 319). We recorded its presence in *Anotomys* and several Amazonian *Neusticomys* (*e.g*., *Neusticomys venezuelae*, *Neusticomys oyapocki*, *Neusticomys ferreirai*).

**Figure 29:**
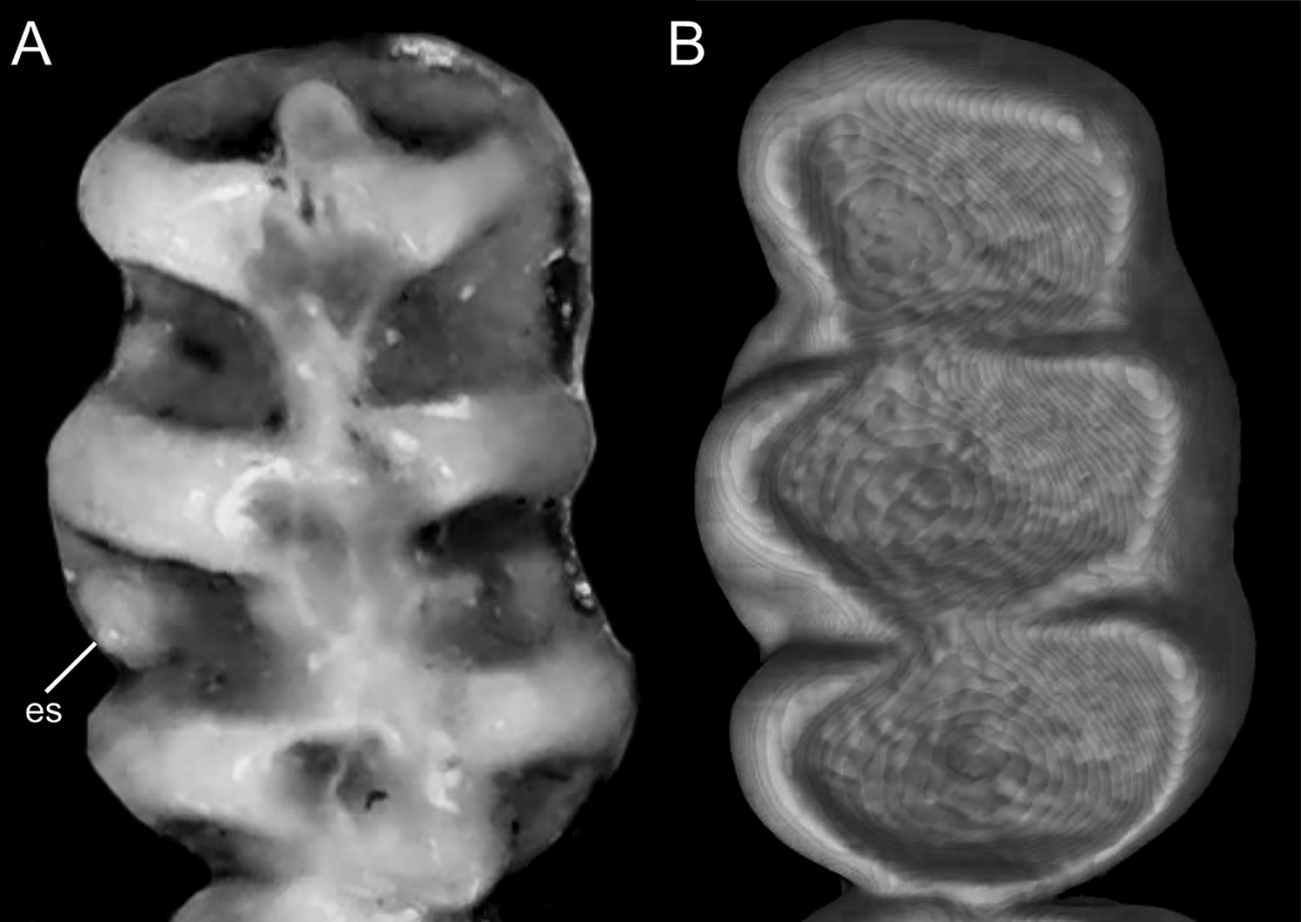
Variability in the M1. The figure (left M1 in occlusal view), illustrates the observed variation in the presence/absence of the enterostyle [es] in M1 of ichthyomyine rodents (character 39). (A) Present (*Anotomys leander*; AMNH 244605) and (B) absent (*Ichthyomys orientalis*; MECN 4914).

Character 39. Enterostyle in M1: (0) present; (1) absent (Fig. 29). A minute enameled cusp developed on the lingual border of M1-M2, between proto and hypocone, being autapomorphic of *Anotomys*.

**Figure 30:**
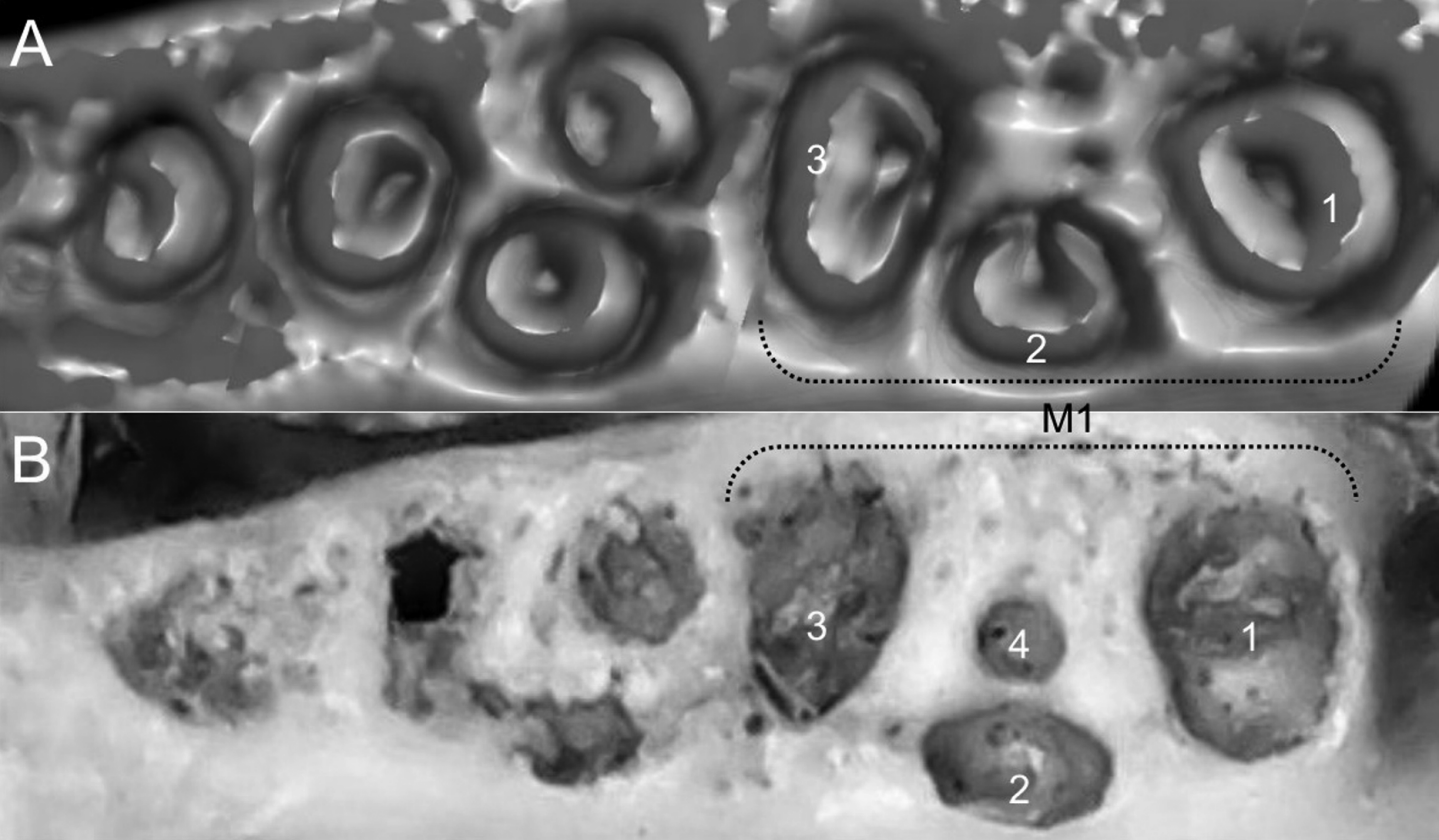
Ventral view of the alveoli of right upper molars of ichthyomyines. Illustrates the variation in the number of roots in M1 (character 40). (A) 3 (*Ichthyomys orientalis*; MECN 4914); (B) 4 (*Neusticomys monticolus*; FMNH 71221).

Character 40. Number of roots in M1: (0) 3; (1) 4. Although it was indicated that M1-M2 are three-rooted in Ichthyomyini (Voss, 1988: 319), C. Ronez, (2022, personal communication) detected at least one *Neusticomys* and two *Rheomys* with four roots in the M1 (Fig. 30). The inspection of roots is limited due to the complete condition of several specimens, an obvious explanation of the high number of taxa with missing data for this character.

**Figure 31:**
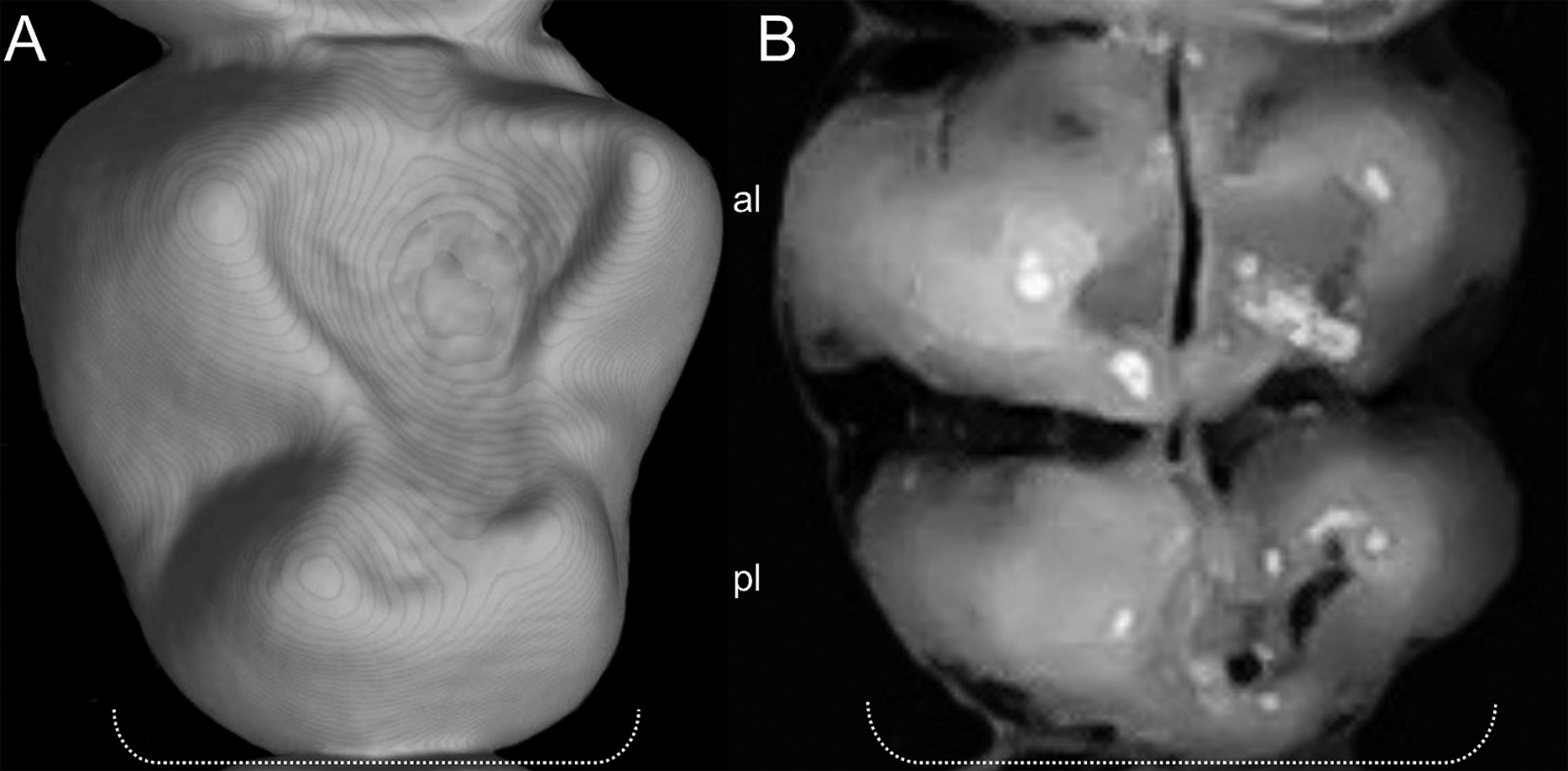
Variability in the M2 in ichthyomyine rodents. This figure (left M2 in occlusal view) illustrates the observed variation in the relative development (in size) between the posterior lobe [pl] and the anterior lobe [al] in the M2 (character 41) in ichthyomyine rodents. (A) pl smaller (*Neusticomys vossi*; MECN 4332); (B) pl subequal (*Rheomys raptor*; USNM 565826).

Character 41. M2 posterior lobe size (respect to anterior lobe): (0) smaller; (1) subequal. With advanced wear, ichthyomyine M2s tend to acquire a general bilobated morphology due to the simplification of occlusal structures (Fig. 31). A few taxa (*e.g*., *Chibchanomys* n. sp., *Neusticomys oyapocki*, *Rheomys mexicanus*) exhibit the posterior lobe, composed by the main cusps pair hypocone-metacone, smaller (when its measured transversally) than the anterior lobe (the pair protocone-paracone). The occurrence of this trait in taxa with absence of M3 (character 43) invites to further explorations (see discussion in Voss, Lunde & Simmons, 2001:101).

**Figure 32:**
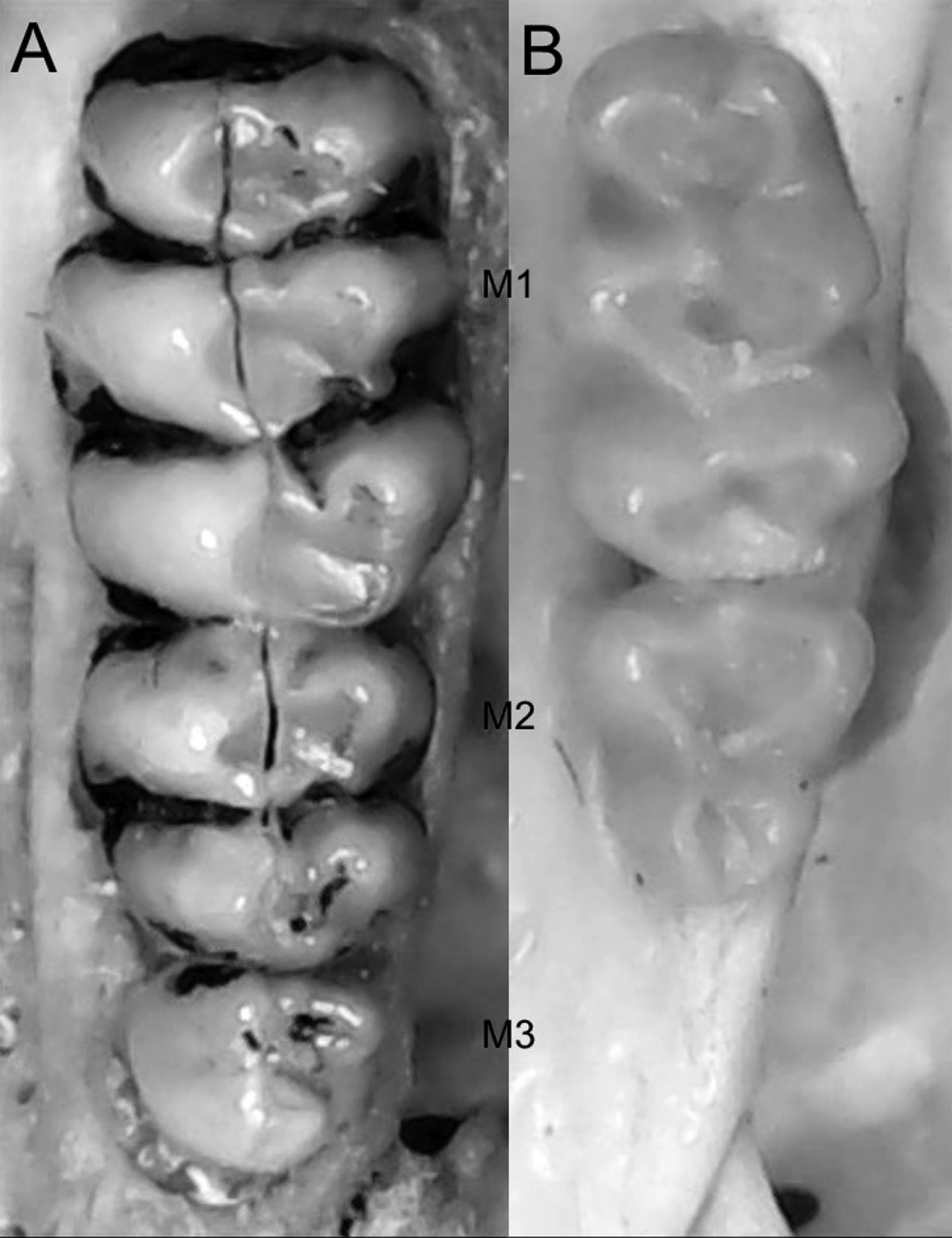
Occlusal view of the left upper molar toothrow in ichthyomyines. The figure illustrates variation in the presence of the M3 (character 42). (A) Present (*Rheomys raptor*; USNM 565826); (B) barely absent (one side) or absent (both sides) (*Daptomys oyapocki*; AMNH 267597).

Character 42. M3: (0) present; (1) barely absent (one side) or absent (both sides); Fig. 32. The condition “M3/m3 small or absent” was recorded as diagnostic for the tribe (Voss, 1988:319). Voss (1988) reported that *Neusticomys oyapocki* was the single sigmodontine with just two upper and lower molars, traits also considered diagnostic at specific level (Dubost & Petter, 1979). Later contributions demonstrated that *N. ferreirai* shared with *N. oyapocki* the occasional absence of M3 (Percequillo, Carmignotto & Silva, 2005), but also that this absence is not diagnostic for the latter, because important variability was detected (Catzeflis et al., 2017). In addition, at least two published specimens of *Ichthyomys pittieri* lack M3 in one or both quadrants (see García et al., 2012: fig. 3; García & Aular, 2016: fig. 3).

**Figure 33:**
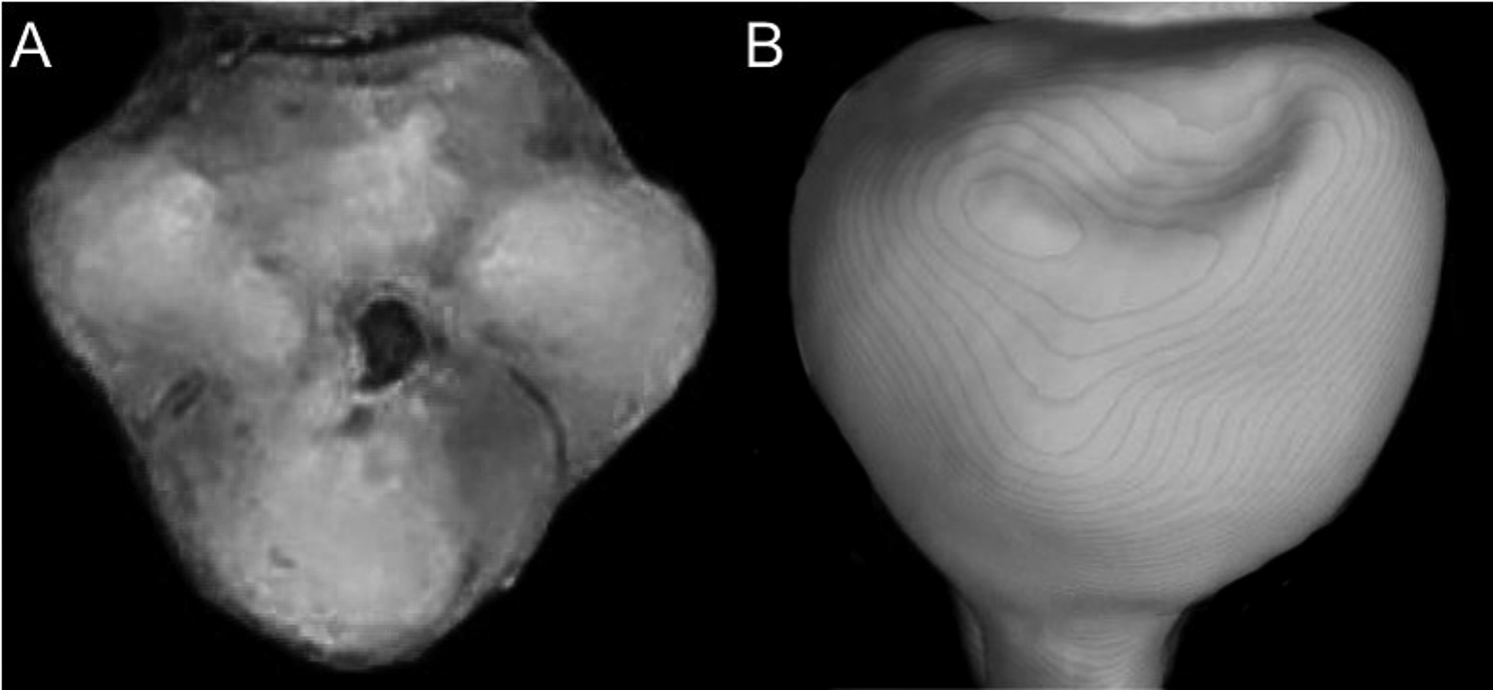
Detail of the morphology of M3. This figure (left M3 in occlusal view) illustrates the variation in the general morphology of the M3 (character 43). (A) “complex” (*Anotomys leander*; AMNH 66202); (B) peg-like (*Neusticomys vossi*; MECN 4332).

Character 43. General M3 morphology: (0) “complex” (bi or tricuspidated); (1) peg-like. Although always small and largely smaller than the M2, the M3 shows some complexity in several taxa (Fig. 33), including the presence of three (*Anotomys leander*, *Rheomys underwoodi*) or two main cusps (*Ichthyomys* spp. *Rheomys mexicanus*, *Rheomys raptor*, *Rheomys thomasi, Chibchanomys trichotis*, *Chibchanomys* n. sp.). Markedly small M3 with undifferentiated occlusal morphology is recorded in *Chibchanomys orcesi* and the species of *Neusticomys*.

**Figure 34:**
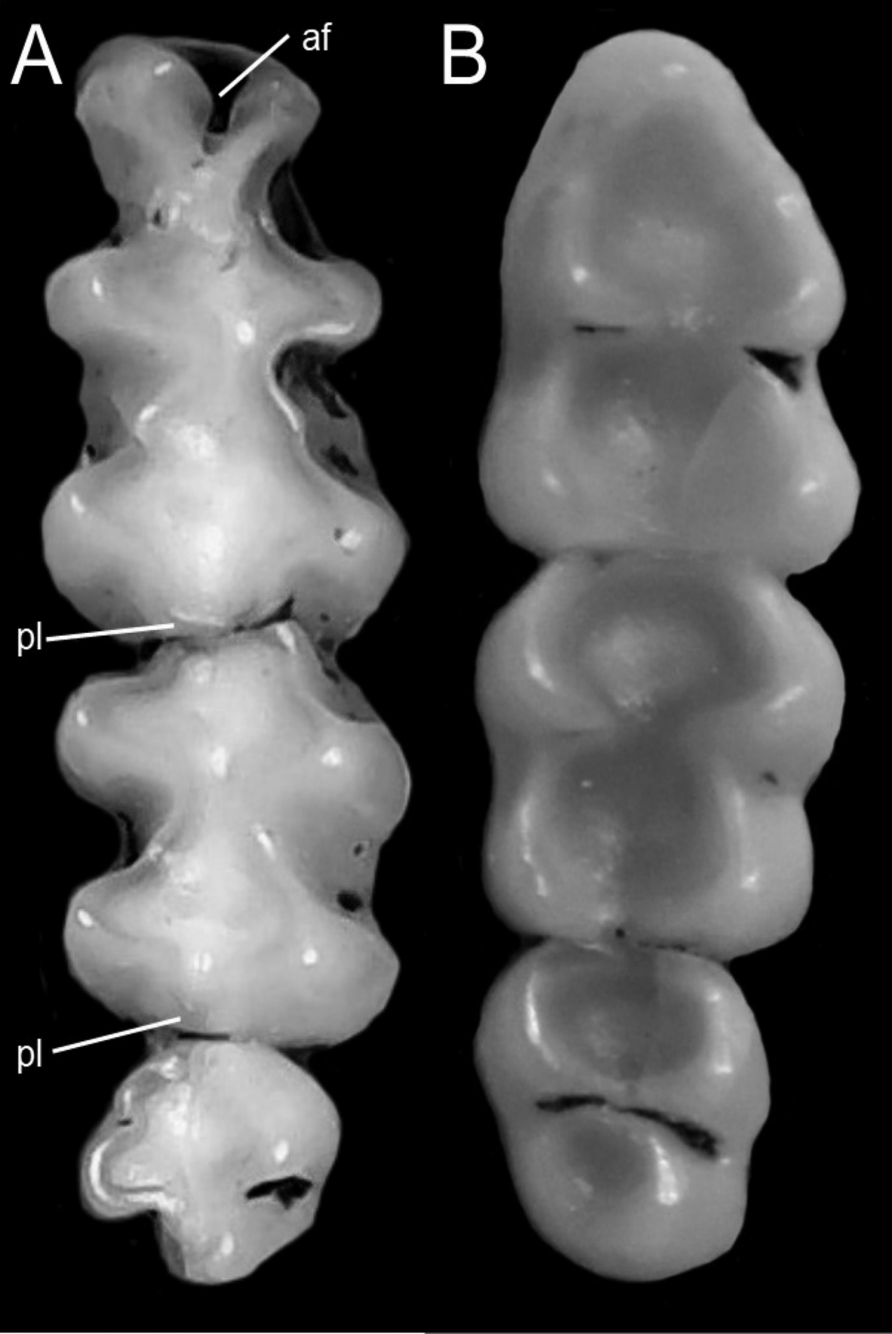
Occlusal view of right lower molar rows of species of ichthyomyines. Illustrates the observed variation in the presence of the posterolophid [pl] in the lower molars (character 44). (A) Present (*Anotomys leander*; AMNH 244605); (B) absent (*Ichthyomys teweedii*; MECN 5772). Other abbreviation: af, anteromedian flexid.

Character 44. Posterolophid in lower molars: (0) present; (1) absent. This character follows the general trend described for ch. 35. Posterolophids are present in the m1 and m2 of *Anotomys leander*, *Chibchanomys trichotis*, *Chibchanomys* n. sp., high-Andean *Neusticomys* and *Neusticomys ferreirai* (although in this species the posterolophid is relatively reduced). The remainder ichthyomyines lack this accessory dental structure (Fig. 34).

**Figure 35:**
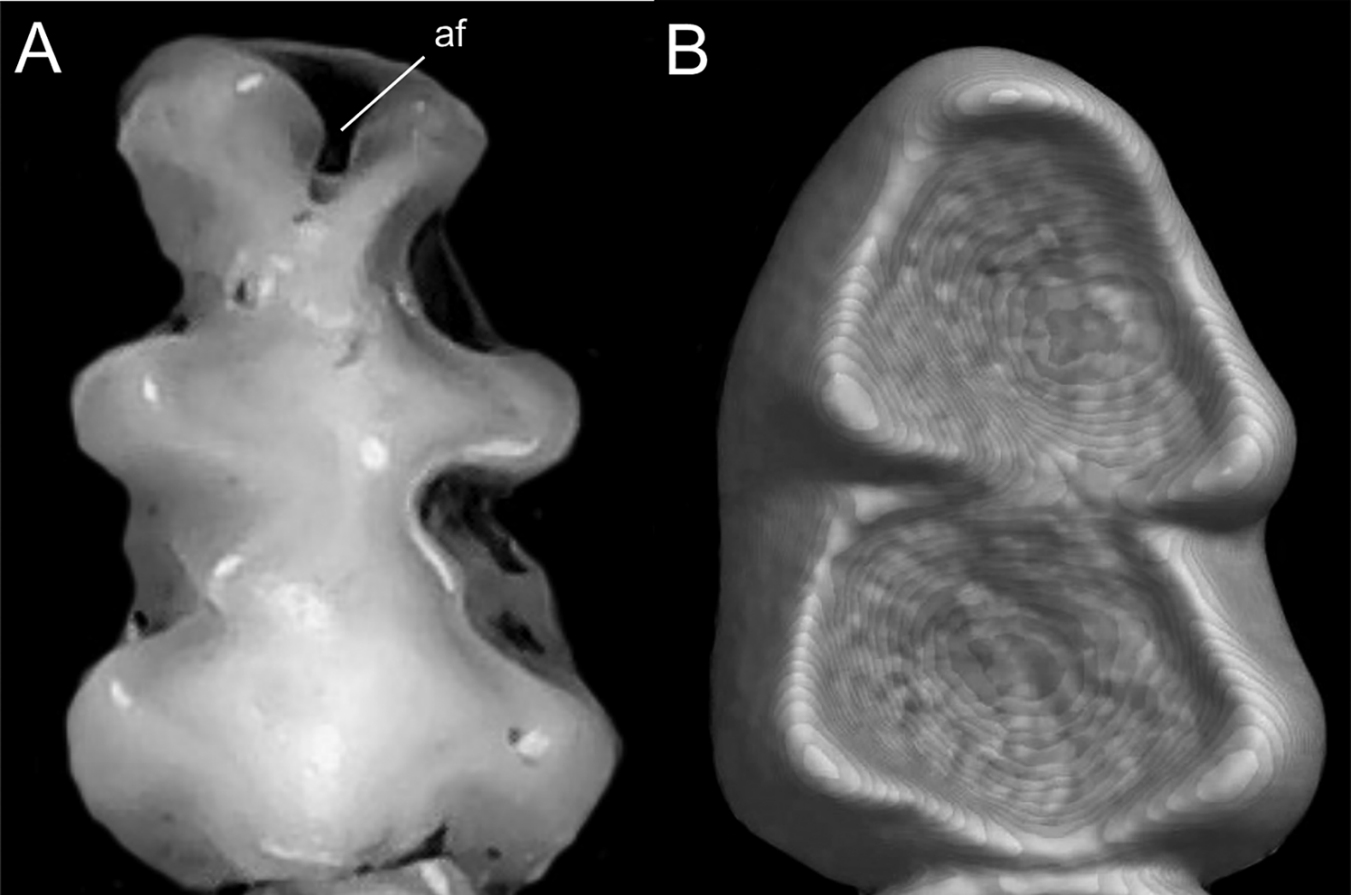
Detail of the first lower molar in ichthyomyines. This figure (right m1 in occlusal view) illustrates the variation in the presence of the anteromedian flexid [af] in m1 (character 45). (A) Present (*Anotomys leander*; AMNH 244605); (B) absent (*Ichthyomys orientalis*; MECN 4914).

Character 45. Anteromedian flexid in m1: (0) present; (1) absent. This character follows the general trend described for ch. 39. Poorly developed anteromedian flexids are recorded in *Anotomys*, *Chibchanomys*, the high Andean species of *Neusticomys*, and *Rheomys* (Fig. 35).

**Figure 36:**
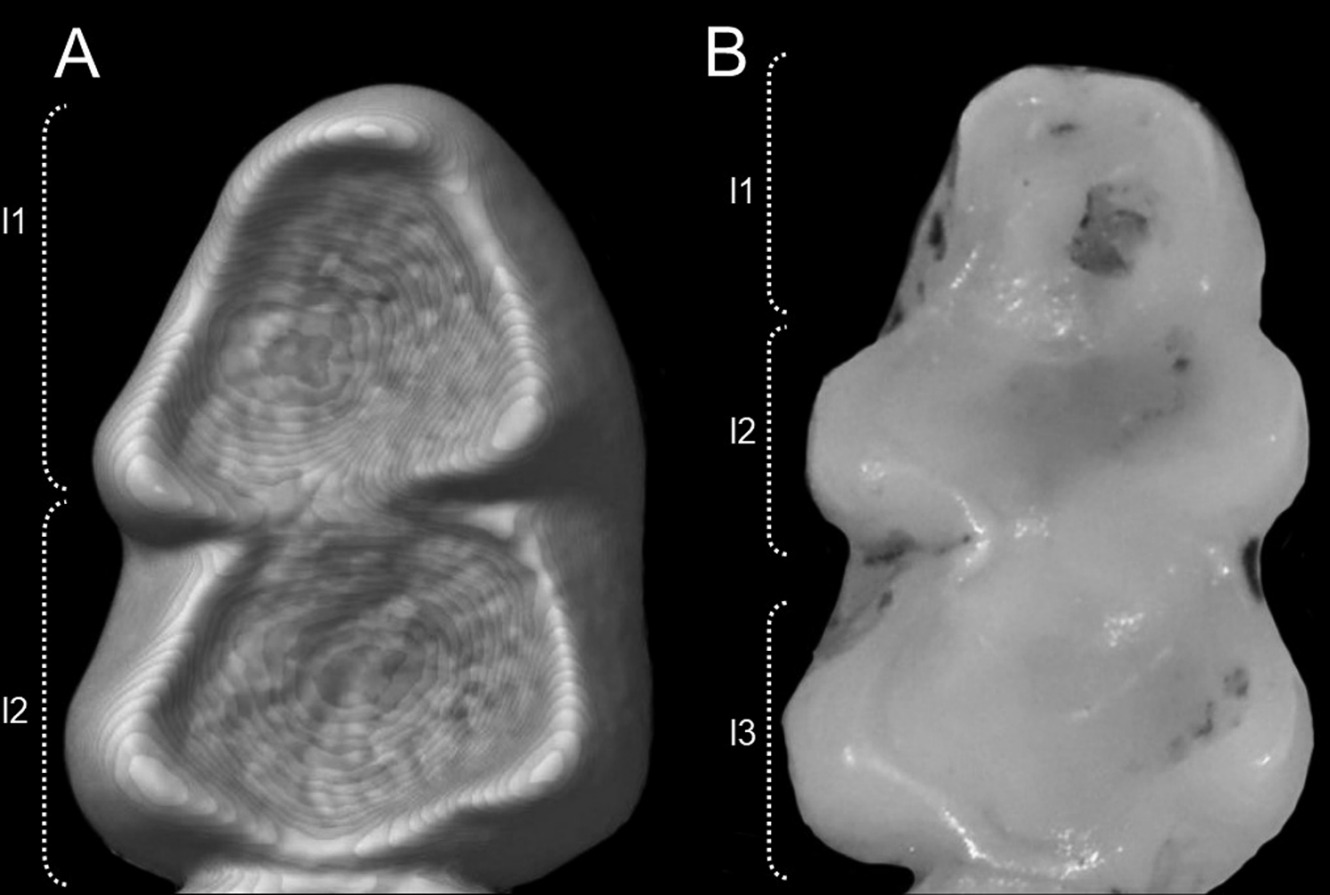
Occlusal views of the first lower molar in ichthyomyines. The figure (left m1 in occlusal view) illustrates variations in the general morphology of m1 in ichthyomyines as they attain the bilobate condition with age (character 46). (A) Present (*Ichthyomys orientalis*; MECN 4914); (B) absent (*Neusticomys orcesi*; MEPN 12230). Acronyms: l1–l3, lobes.

Character 46. Bilobated m1: (0) present; (1) absent. The m1 of some ichthyomyines, particularly *Ichthyomys*, *N. vossi*, and *R. thomasi*, acquire, with advancing wear, a bilobated occlusal expression (Fig. 36). The remainder forms retain the classical condition of three lobes (anteroconid, pair protoconid-metaconid, and pair hypoconid-entoconid).

**Figure 37:**
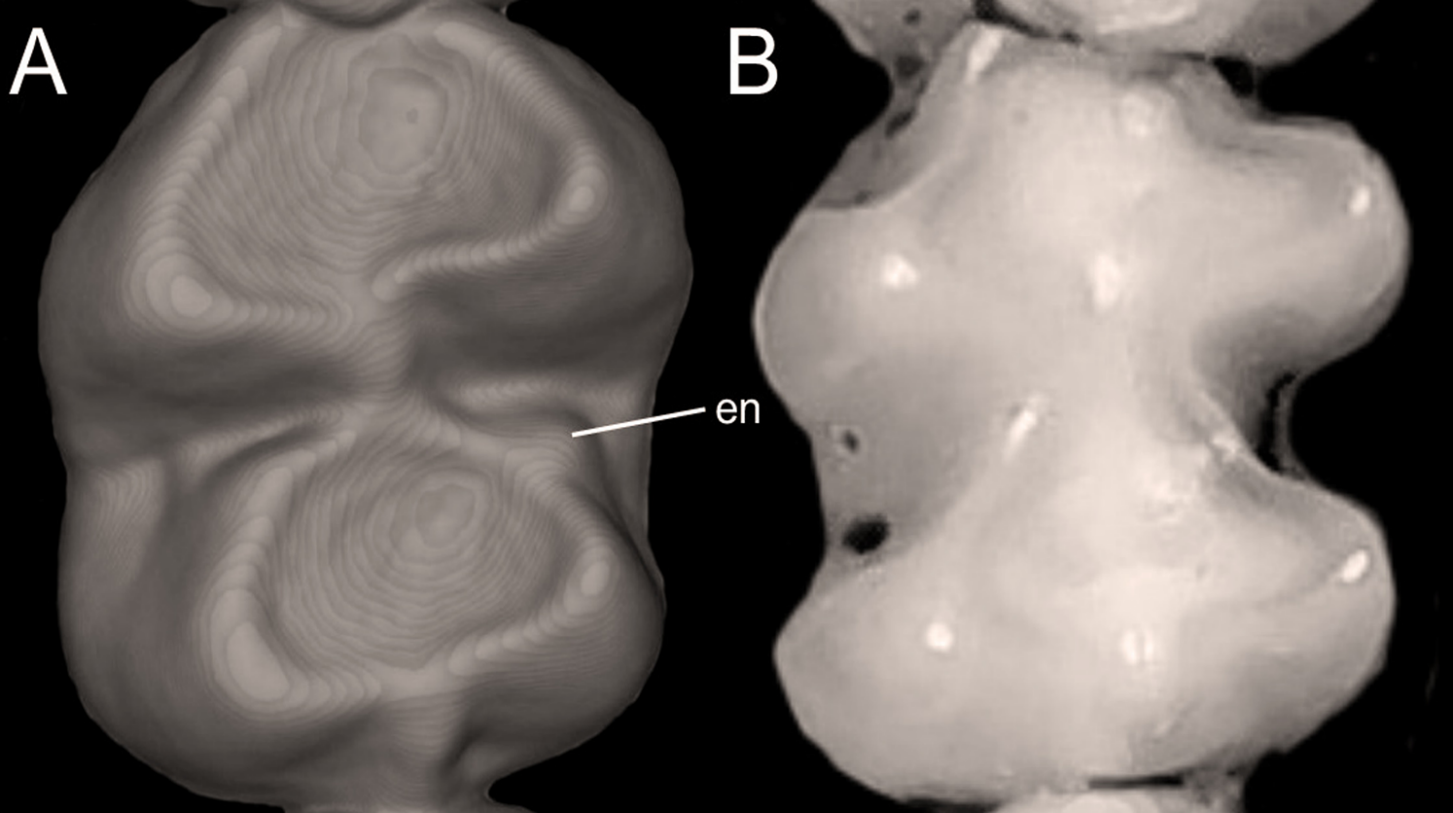
Detail of the m2. Occlusal views of the left m2 in ichthyomyine rodents showing variations in the presence of a entolophulid-like structure [en] in m2 (character 47). (A) Barely present (*Neusticomys vossi*; MECN 4332); (B) absent (*Anotomys leander*; AMNH 244605).

Character 47. Entolophulid-like in m2: (0) barely present; (1) absent. A tiny enameled structure, apparently an entolophulid in a classical topographical approach (*i.e*., Reig, 1977), is recorded in *Chibchanomys trichotis*, *Neusticomys monticolus*, *Neusticomys vossi*, *Neusticomys ferreirai* and *Rheomys underwoodi* (Fig. 37).

**Figure 38:**
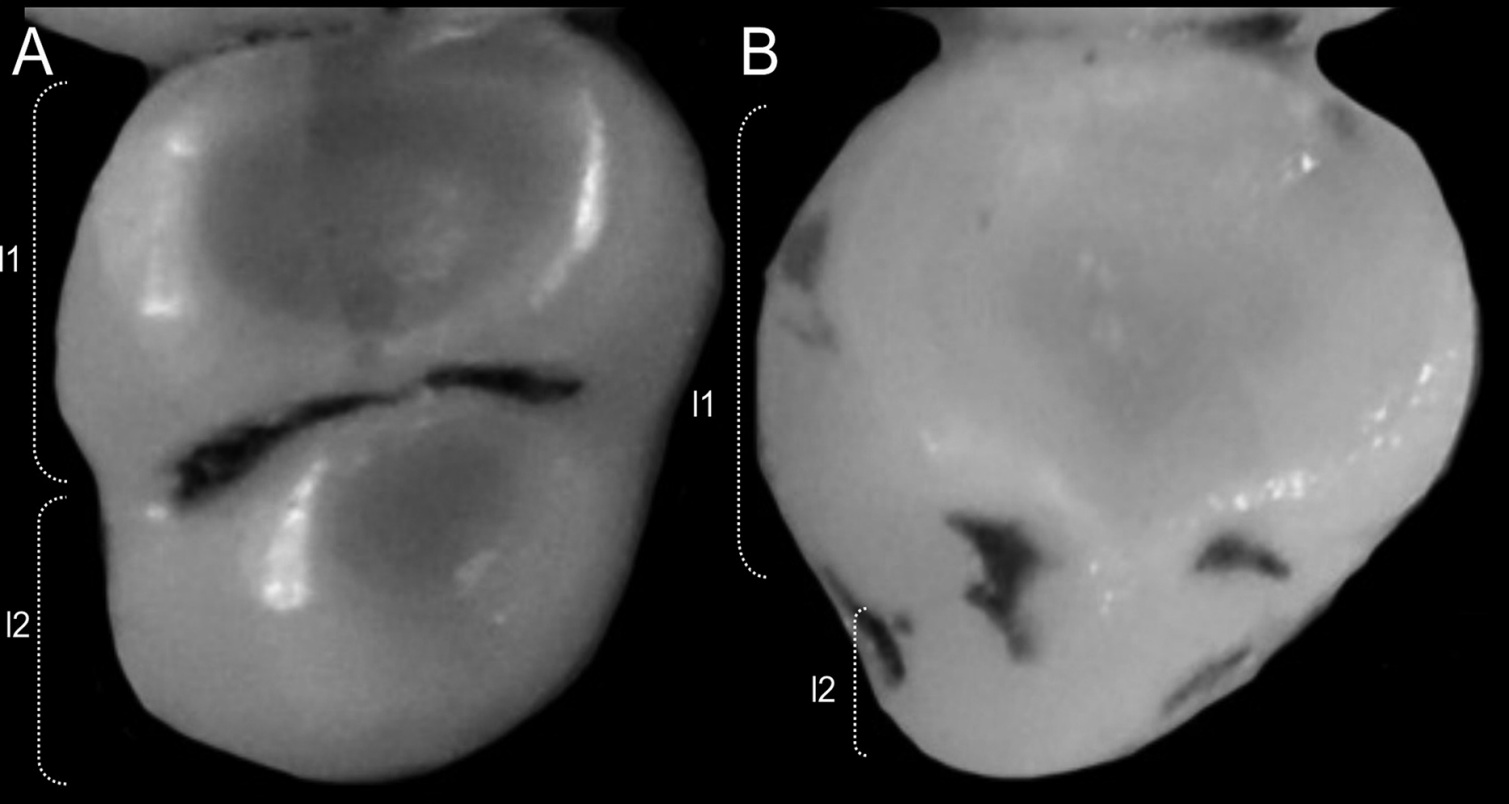
Detail of the m3 in ichthyomyines. The figure (left m3 in occlusal view) shows the variation in the general morphology of the m3 (character 48) in ichthyomyines. (A) With two distinct lobes (*Ichthyomys tweedii*; MECN 5772); (B) with the posterior lobe small or absent (*Neusticomys orcesi*; MEPN 12230). Acronyms: l1–l2, lobes.

Character 48. General m3 morphology: (0) with 2 distinct lobes; (1) with the posterior lobe small or directly absent. The description and coding of this character follows Voss (1988, ch. 9). A more or less developed posterior lobe, composed by the pair hypoconid-entoconid, is recorded in *Anotomys leander*, *Chibchanomys* (except *C. orcesi*), *Ichthyomys* and *Rheomys*. Simplified m3s, those with the posterior lobe very small or even absent, are the conditions observed in *Chibchanomys orcesi* and *Neusticomys* (Fig. 38).

### Soft anatomy

Character 49. Omohyoid muscle: (0) present; (1) absent. The description and coding of this character follows Voss (1988, ch. 15). The omohyoid muscle is absent in *Anotomys* and *Rheomys mexicanus*.

**Figure 39:**
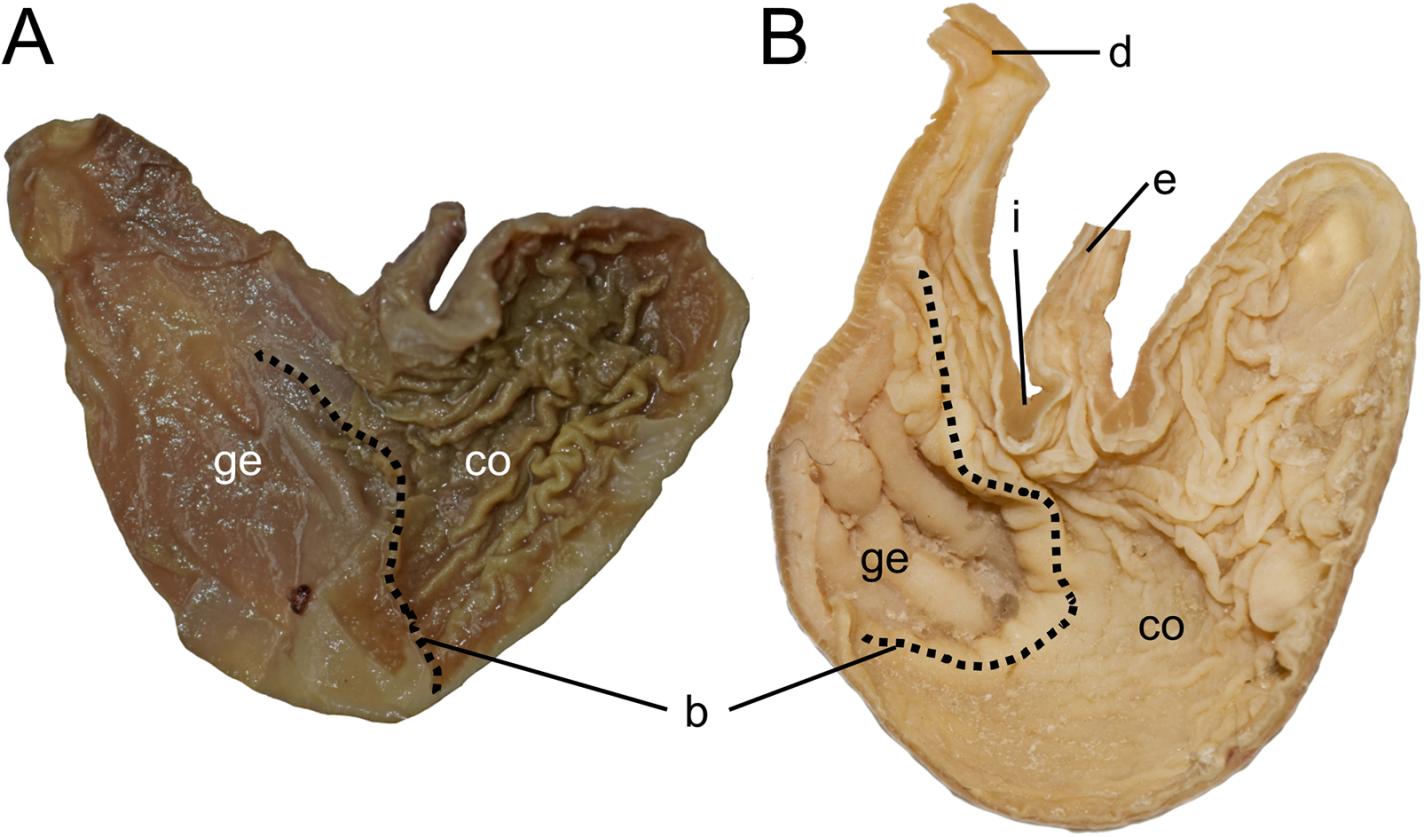
Details of the stomachs. These ventral views (in midfrotal section) illustrate variations in the extent of gastric glandular epithelium (character 50) in ichthyomyine rodents. (A) Glandular epithelium present between the incisura angularis and the pyloric sphincter (A *Daptomys* sp.; JBM 2500 (same individual as MECN 6629)); (B) glandular epithelium restricted to the greater curvature (*Ichthyomys orientalis*; MEPN 12672). Abbreviations: b, bordering fold; d, duodenum; co, cornified epithelium; ge, glandular epithelium; i, incisura angularis.

Character 50. Gastric glandular epithelium: (0) glandular epithelium present between the *incisura angularis* and the pyloric sphincter; (1) glandular epithelium restricted to the greater curvature of the stomach. The description and coding of this character follows Voss (1988, ch. 16). Examples of stomach gross morphology for members of the tribe were studied by Vorontsov (1967), Carleton (1973), Voss (1988), and Férnandez de Córdova et al. (2020). All ichthyomyines possess unilocular-hemiglandular stomachs (*sensu* Carleton, 1973), varying regarding the extension of the glandular epithelium. The latter lines the antrum including the pyloric region in *Chibchanomys* and *Neusticomys*, while in *Anotomys*, *Ichthyomys*, and *Rheomys*, the glandular portion is mostly restricted to the greater curvature (Fig. 39).

Character 51. Gallbladder: (0) present; (1) absent. The description and coding of this character follows Voss (1988, ch. 17). The gallbladder absence is, in the context of the specimens surveyed, autapomorphic of *Ichthyomys*.

Character 52. Bacular cartilage: (0) tridigitate; (1) unidigitate bacular cartilage with a single digit (derived from state 0); (2) bacular cartilage tridigitate, the medial digit grossly swollen with a well-defined calcified core (derived from state 0). The description and coding of this character follows Voss (1988, ch. 18; see also Hooper & Musser, 1964). States (1) and (2) are autapomorphies of *Anotomys* and *Rheomys*, respectively.

**Figure 40:**
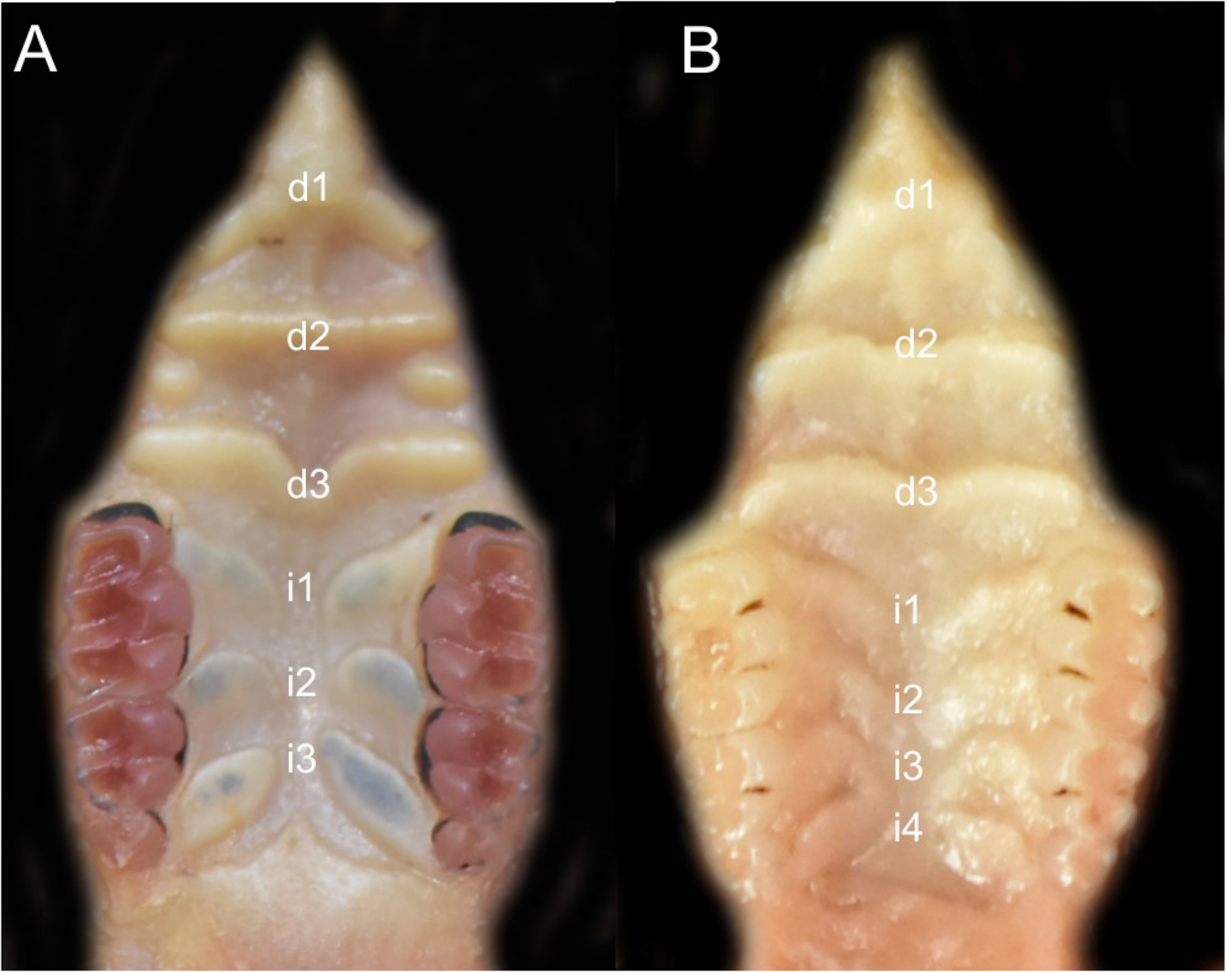
Details of the soft palate. The figure (soft palate in ventral view) shows variations in the number of interdental rugae (character 53) in ichthyomyine rodents. (A) Three (*Daptomys* sp.; JBM 2500 (same individual as MECN 6629)); (B) four (*Ichthyomys tweedii*; MECN 5772). Abbreviations: d1–d3, diastemal rugae; i1–i4, interdental rugae.

Character 53. Number of interdental rugae: (0) 3; (1) 4. Voss (1988:312) summarized the observed variation of the rugae of ichthyomyine soft palate as composed of “three diastemal rugae and three to four intermolar rugae.” The widespread condition seems to be the state (1); *Anotomys leander*, *Chibchanomys trichotis*, *Neusticomys venezuelae* and *Rheomys thomasi* have three interdental rugae (Fig. 40).

### Postcranial skeleton

Character 54. Number of ribs: (0) 13; (1) 14. This character was based on counts provided by Voss (1988: table 10).

Character 55. Number of thoracolumbar vertebrae: (0) 19; (1) 20. This character was based on counts provided by Voss (1988: table 10), except for *Neusticomys ferreirai* which exhibits 18 or 19 elements.

Character 56. Number of sacral vertebrae: (0) 4; (1) > 4. This character was based on counts provided by Voss (1988: table 10).

## Appendix 2

New classificatory arrangement for the tribe Ichthyomyini based on total evidence phylogenetic analysis, with the definition of the taxa recognized, including diagnosis, contents, and remarks.

### Classification

Tribe Ichthyomyini Cockerell & Printz in Cockerell, Miller & Printz (1914)

Subtribe Ichthyomyina Cockerell & Printz in Cockerell, Miller & Printz (1914)

Genus *Chibchanomys* Voss, 1988

Genus *Ichthyomys* Thomas, 1893

Genus *Rheomys* Thomas, 1906b

Genus *Neusticomys* Anthony, 1921

Genus *Daptomys* Anthony, 1929

Subtribe Anotomyina nov. subtribe

Genus *Anotomys* Thomas, 1906a

**Remarks:** Tribe authorship was traditionally attributed to Vorontsov (1959). Recently, Cazzaniga, Cañón & Pardiñas (2019) demonstrated that Ichthyomyini was properly named and diagnosed by Cockerell & Printz in Cockerell, Miller & Printz (1914).

**Definition**: The clade composed of the last common ancestor of *Anotomys*, *Chibchanomys*, *Ichthyomys*, *Rheomys*, *Neusticomys*, *Daptomys*, and all its descendants.

**Tribe ICHTHYOMYINI Cockerell & Printz in Cockerell, Miller & Printz (1914)**

**Type genus:** *Ichthyomys* Thomas, 1893

**Diagnosis:** Ichthyomyines are small to medium-sized (15–140 g) semiaquatic carnivorous rodents that are morphologically distinctive in many external, skeletal, and visceral characters. Pinnae small, well haired or absent; mystacial vibrissae stiff and abundant, the hairs of the tumid upper lip ventrally recurved; genal vibrissae absent; mammae six, in postaxial, abdominal, and inguinal pairs; pes with a continuous comb of stiff hairs along the plantar margins (weakly developed in some species); hypothenar pad of pes absent or indistinct (except in some specimens of *Anotomys*); tail well haired, the underlying epidermal scales inconspicuous; ventral caudal hairs much longer and denser than hairs of the caudal dorsum. Incisors opisthodont but approaching the orthodont condition; molars 3/3, 3/2 or 2/2, rooted, biserial, the left and right maxillary series parallel; labial and lingual cusps arranged in strictly opposite pairs; Ml anterocone always with two functional cusps but the anteromedian flexus shallow, indistinct, or absent; mesoloph(id)s, anteroloph(id)s, posteroloph(id)s, and other accessory enameled structures of the molar crowns small and inconspicuous or absent; M3/m3 small, variably present inter and interspecifically; M1-2 with three roots, m1-2 with two roots each. Zygomatic notches completely absent; interorbital margins rounded, without beads or sharp edges; braincase smooth in lateral aspect, without conspicuous temporal ridges; gnathic process of premaxillae well developed, projecting conspicuously between upper incisors; inferior zygomatic roots slender, without platelike anterior projections or dorsal spines; masseteric tubercles near base of inferior zygomatic roots well developed; hard palate smooth between the molar rows and produced posteriorly beyond M3; parapterygoid fossae narrow, not deeply excavated; incisive foramina long penetrating or not between molar rows (not deeply penetrating, reaching the anterocone of M1); posterolateral palatal pits few and simple; infraorbital foramen large and ovoid, as wide ventrally as dorsally; lateral wall of rostrum complete behind inferior zygomatic root, not fenestrated; optic foramen very small; buccinator-masticatory foramen separated from foramen ovale accessorius by stout vertical strut of the alisphenoid; sphenopalatine vacuities absent or present only as narrow slits, the bony roof and walls of the mesopterygoid fossa substantially complete; subsquamosal fenestra absent; stapedial foramen always present but sometimes small; auditory bullae small, flaskshaped or globular; tympanic membrane pars flaccida absent. Neural spine on third thoracic vertebra enlarged for attachment of nuchal ligament; head of first rib articulates with transverse processes of seventh cervical and first thoracic vertebrae; entepicondylar foramen absent; cheek pouches absent; tongue with one circumvallate papilla; stomach unilocular with glandular epithelium variously reduced from hemiglandular condition; small intestine very long; large intestine very short, simple, without colonic loops; caecum short, simple and vermiform; complete muroid complement of accessory glands present; heads of spermatozoa oval with a single apical hook; glans penis spinous externally with deep terminal crater containing dorsal papilla(e), bacular mound(s), two spinous lateral papillae, and a bifurcate urethral process (after Voss, 1988:319–320).

**Subtribe Ichthyomyina Cockerell & Printz in Cockerell, Miller & Printz, 1914**

**Type genus:** *Ichthyomys* Thomas, 1893

**Diagnosis:** Ichthyomyines with dull, gray black, or glossy, grizzled-brownish, adult dorsal pelage; pinnae typically visible above fur, or buried in the fur of the head (*Chibchanomys*); philtrum present (*Ichthyomys*, *Neusticomys*, *Rheomys*) or absent (*Chibchanomys*, *Rheomys*); tail longer, equal to or less than head-and-body length; manus typically with five or four separate plantar pads (*Rheomys*), hindfoot broad with well-developed fringing hairs (*Anotomys*, *Ichthyomys*, *Chibchanomys*), or narrow with weakly developed fringing hairs (*Daptomys*, *Neusticomys*); nasal bones typically long, rarely short and truncated (*Ichthyomys*); supraorbital foramina typically open laterally, rarely dorsally (*Ichthyomys*) within the orbital fossae; carotid circulation pattern 1 (*Chibchanomys*, *Daptomys*, *Neusticomys*) or 3 (*Ichthyomys*, *Rheomys*); orbicular apophysis of malleus absent (*Chibchanomys*, *Neusticomys*, *Daptomys*) or present (*Ichthyomys*, *Rheomys*); stomach unilocular-hemiglandular with gastric glandular epithelium restricted to greater curvature (*Ichthyomys*, *Rheomys*) or occupying most of the antrum (*Chibchanomys*, *Daptomys*, *Neusticomys*); gall bladder typically present (absent in *Ichthyomys*); bacular cartilage tridigitate (after Voss, 1988; this paper).

**Included genera:** *Chibchanomys* Voss, 1988; *Daptomys* Anthony, 1929; *Ichthyomys* Thomas, 1893; *Neusticomys* Anthony, 1921; *Rheomys* Thomas, 1906b.

**Genus *Chibchanomys* Voss, 1988**

**Type species:** *Chibchanomys trichotis* (Thomas, 1897)

**Diagnosis:** Medium ichthyomyines with dull, grayblack dorsal pelage and large and not abundant guard hairs; reduced pinnae buried in the fur of the head; philtrum absent; tail longer than combined length of head and body; manus with five separate plantar pads; metatarsal configuration: IV > III > II ≥ V > I; hindfoot long and broad with well-developed fringing hairs; nasal bones long; supraorbital foramina open laterally within orbits; carotid circulation pattern 1; orbicular apophysis of malleus absent; stomach with glandular epithelium between esophagus and pyloric sphincter; gall bladder present; bacular cartilage tridigitate (after Voss, 1988:322).

**Included species:** *Chibchanomys trichotis* (Thomas, 1897); and *Chibchanomys* n. sp.

**Remarks:** Originally described as monotypic, *Chibchanomys* diversity was doubled with the description of *C. orcesi* Jenkins & Barnett, 1997, a species here allocated under *Neusticomys* (see below). However, at least one undescribed species is apparent in *Chibchanomys*, based on recently collected animals at Machupicchu (Peru; H. Zeballos, 2021, unpublished data) and Huánuco (Peru, specimen LSUMZ 14406) formerly referred to *Anotomys leander* (see Gardner, 1971) and currently identified as *Chibchanomys orcesi* (Voss, 2015:283).

**Genus *Ichthyomys* Thomas, 1893**

**Type species:** *Ichthyomys stolzmanni* Thomas, 1893

**Diagnosis:** Large ichthyomyines with glossy, grizzled-brownish adult dorsal pelage with abundant guard hairs, countershaded ventral and dorsal pelage; small pinnae visible above fur of the head; philtrum present and broad; equal to or less than head-and-body length; manus with five separate plantar pads; metatarsal configuration: IV > III > II ≥ V > I; hindfoot long relative to head-and-body but proportionately broad with well-developed fringing hairs; nasal bones short, truncated; supraorbital foramina open dorsally between the orbits in large adults and lateral in young animals; carotid circulation pattern 3; orbicular apophysis of malleus present; stomach with glandular epithelium restricted to greater curvature; gall bladder absent; bacular cartilage tridigitate (after Voss, 1988:328).

**Included species:** *Ichthyomys hydrobates* (Winge, 1891), including as junior synonyms: *I. soderstromi* de Winton, 1896 and *I. h. nicefori* Thomas, 1924a; *Ichthyomys orientalis* Anthony, 1923; *Ichthyomys pinei* Férnandez de Córdova et al., 2020; *Ichthyomys pittieri* Handley & Mondolfi, 1963; *Ichthyomys stolzmanni* Thomas, 1893; and *Ichthyomys tweedii* Anthony, 1921, includes *I. caurinus* Thomas, 1924b, as a junior synonym.

**Remarks:** Beyond the recent addition of *I*. *pinei* from southern Ecuador (see Férnandez de Córdova et al., 2020), we reinstated *I*. *orientalis* (type locality as near río Napo, Napo, Ecuador; Anthony, 1923) as full species, previously retained as subspecies by Voss (1988) but directly treated as junior synonym of *I. s. stolzmanni* by Pacheco & Ugarte-Núñez (2011). According to our evidence, the specimen (MECN 4914) previously assigned to *I. stolzmanni* from río Jurumbuno (see Brito, Tenecota & Pozo-Zamora, 2016, Table S1), forms a clade with one individual from Tungurahua, Ecuador (QCAZM 17512). This clade, is in turn recovered as the sister group to *I. hydrobates* and only distantly related to *I. stolzmanni*.

**Genus *Rheomys*** Thomas, 1906b

**Type species:** *Rheomys underwoodi* Thomas, 1906b

**Diagnosis:** Large to small ichthyomyines with glossy, grizzled-brownish adult dorsal pelage; pinnae visible or not above fur of head; philtrum present or absent; tail shorter, equal to or longer than head-and-body length; manus with four or fewer separate plantar pads; hindfoot short or long relative to head-and body but proportionately broad with well-developed fringing hairs; nasal bones long; supraorbital foramina open laterally within the orbital fossae; carotid circulation pattern 3; orbicular apophysis of malleus present; stomach with gastric glandular epithelium restricted to greater curvature; gall bladder present; bacular cartilage tridigitate (Voss, 1988:347).

**Included species:** *Rheomys raptor* Goldman, 1912, includes *R. hartmanni* Enders, 1939; *Rheomys thomasi* Dickey, 1928, includes: *R. t. stirtoni* Dickey, 1928 and *R. t. chiapensis* Hooper, 1947; *Rheomys mexicanus* Goodwin, 1959; and *Rheomys underwoodi* Thomas, 1906b.

**Remarks:** Alpha-taxonomy in *Rheomys* needs a deep revision. With the scarce evidence at hand, two main groups can be recognized, one grouping large-bodied *Rheomys* (*R. mexicanus* and *R. underwoodi*), the other including small-bodied forms (*R. raptor* and *R. thomasi*). Because the subgenus *Neorheomys* Goodwin, 1959, is based on *R. mexicanus*, and *Rheomys* Thomas, 1906b is based on *Rheomys underwoodi*, current subgeneric names refer to the same taxon and should be considered synonymous. If the current division of species groups based on size is maintained, a new taxon of subgeneric (or even generic status) may need to be erected to include them. Our representatives of *R. raptor* correspond to *R. raptor hartmanni*, not the nominal form; our results also evidence an undescribed species of *Rheomys* from the Cordillera de Tilarán, Costa Rica (KU 159017). The Tilarán mountain range is an area renowned for its patterns of endemic diversity and because it is a region of biotic transitions (Kohlmann, 2011; Nadkarni & Wheelwright, 2000; Woodman & Timm, 2017).

**Genus *Neusticomys* Anthony, 1921**

**Type species:** *Neusticomys monticolus* Anthony, 1921

**Emended diagnosis:** Small to medium ichthyomyines (body mass range 24–40 g) with dull grayblack brownish adult dorsal pelage, while the ventral pelage slightly paler than the dorsum; pinnae conspicuous above fur of head; philtrum present; tail slightly shorter than head-and-body length; manus with five separate plantar pads, metatarsal configuration: III ≥ IV > II >> V > I; hindfoot narrow and comparatively short, with weakly developed fringing hairs and very short hallux; rostrum slender; inferior root zygomatic plate even with or posterior to the first upper molar; nasal bones long; supraorbital foramina open laterally into orbits; occipital condyles not projecting posteriorly beyond rest of occiput; carotid circulation pattern 1; orbicular apophysis of malleus present; third lower molar peg-like; stomach with glandular epithelium between esophagus and pyloric sphincter; gall bladder present; bacular cartilage tridigitate (after Anthony, 1921; Voss, 1988; Hanson et al., 2015).

**Included species:** *Neusticomys monticolus* Anthony, 1921; *Neusticomys orcesi* (Jenkins & Barnett, 1997); *Neusticomys vossi* Hanson et al., 2015; and *Neusticomys* n. sp.

**Remarks:** *Neusticomys* is here restricted as was originally envisioned by Anthony (1921). We included *Cytb* data and 51 discrete morphological characters for one of the six specimens mentioned by Barnett (1997) and animals from the type series of *orcesi*. Further, first-hand morphological examination of LSUMZ 14406, augmented with analyses of detailed photographs, allowed us to score 46 of 56 discrete morphological characters for this specimen. According to our results, *orcesi* (including LSUMZ 14406), formerly allocated under *Chibchanomys*, belongs to the new concept of *Neusticomys* defined here. A clade grouping specimens from Colombia and retrieved as sister of *N. vossi*, deserves specific rank.

**Genus *Daptomys* Anthony, 1929**

**Type species:** *Daptomys venezuelae* Anthony, 1929

**Emended diagnosis:** Small to medium ichthyomyines (body mass range 20 to 66 g) with glossy, brownish adult dorsal pelage, while the ventral pelage slightly paler than the dorsum; pinnae conspicuous above fur of head; philtrum present; tail shorter than head-and-body length; manus with five separate plantar pads, metatarsal configuration: III ≥ IV > II >> V > I; hindfoot narrow, comparatively short, with weakly developed fringing hairs and very short hallux; rostrum broad and blunt; inferior root zygomatic plate anterior to the first upper molar; incisors heavy but proportionately small molars; nasal bones long; supraorbital foramina open laterally into orbits; occipital condyles projecting posteriorly beyond rest of occiput; carotid circulation pattern 1; orbicular apophysis of malleus absent; third upper molar peg-like if present, M3 and m3 typically absent in *D. oyapocki*; stomach with glandular epithelium between esophagus and pyloric sphincter; gall bladder present; bacular cartilage tridigitate (after Anthony, 1929; Musser & Gardner, 1974; Voss, 1988; Percequillo, Carmignotto & Silva, 2005; Pacheco et al., 2020).

**Included species and subspecies:** *Daptomys ferreirai* (Percequillo, Carmignotto & Silva, 2005); *Daptomys mussoi* (Ochoa & Soriano, 1991); *Daptomys oyapocki* Dubost & Petter, 1979; *Daptomys peruviensis* Musser & Gardner, 1974; *Daptomys peruviensis musseri* Pacheco et al., 2020; *Daptomys venezuelae* Anthony, 1929; *Daptomys* n. sp.

**Remarks:** Our results strongly support the reinstating of *Daptomys* as a valid genus, including an appreciable diversity associated mostly to tropical and subtropical eastern lowlands. Allowing the use of different names for taxa with evident diversity in morphology and ecology produces a classification with heuristic value and is useful to research on adaptation, speciation, and biogeography (Wheeler, 2009). The recently proposed *D. peruviensis musseri* is maintained here at the subspecies level: although the case for its specific status could be made based on criteria such as monophyly and morphology, more data from intermediate localities are needed. *D. ferreirai* is not retrieved as sister to *D. oyapocki* (as was suggested by Voss, 2015:288) but to a clade composed of *D. mussoi*, and *D. peruviensis*. Notably, the distribution of *D. peruviensis* is broad, ranging over several thousand kilometers from Ecuador to Peru in Western Amazon, but not reaching Brazil, as the specimen attributed to this species by Percequillo et al. (2017) is in fact *N. ferreirai*. *D*. *mussoi* and *D*. *peruviensis* are very closely related taxa; the possibility that may even be conspecific, as suggested by Voss (2015), must await additional studies. An undescribed species is here represented by a young specimen from Bolivia (Anderson, 1997), currently the southernmost record for the genus and the tribe.

**Subtribe Anotomyina nov.**

urn:lsid:zoobank.org:act:EB6195F3-3E5E-447D-BF60-D67658C6E874

**Type genus: *Anotomys*** **Thomas, 1906a**

**Diagnosis:** Ichthyomyines with dull, grayblack dorsal pelage, countershaded; pinnae absent; tufts of pure white fur over the external auditory canals present; without philtrum; supraorbital vibrissae present; tail longer than combined length of head and body; manus with only four separate plantar pads, metatarsal configuration: IV > III > V > II > I; hindfoot very large and broad with well-developed fringing hairs; nasal bones long; supraorbital foramina open laterally into orbits; carotid circulation pattern 2; orbicular apophysis of malleus present; stomach with glandular epithelium restricted to greater curvature; gall bladder present; two dorsal crater papillae in the glans penis present (after Voss, 1988:325).

**Remarks:** Anotomyina represents a very long branch that is consistently recovered as the sister lineage of remainder ichthyomyines in phylogenetic analyses of multilocus sequence datasets. The long branch leading to *Anotomys* suggests an ancient history of independent evolution accompanied by extinction of transitional forms. The same conclusion is suggested by its many unique characters. In effect, excluding *Anotomys* from Ichthyomyina simplifies the diagnosis of the latter clade and provides a new higher taxon to accommodate Recent or fossil relatives of the former, should any be discovered.

Subtribal classification in Sigmodontinae is not a widespread practice, although it has been implemented in formal and informal treatments in the past. For example, Steppan (1995: 72) highlighted the possibility of classifying the Phyllotini in three subtribes, the monotypic Calomyina (containing *Calomys*) and the polytypic Phyllotina (*Graomys* and *Phyllotis*) and Reithrodonina (*Andinomys*, *Auliscomys*, and the *Reithrodon* group). His arrangement parallels our hypothesis about ichthyomyines as it reflects an intrinsic asymmetry in subtribal diversity, at least as depicted by living taxa. More recently, Teta et al. (2017) formalized in subtribal hierarchy (two subtribes) the main division largely observed within Abrotrichini. Other authors opted for the recognition of informal groups of genera, denoting intratribal structure by letters—the treatment of Oryzomyini by Weksler (2006)—or employing the rank “Division”—as in D’Elía (2003) regarding Akodontini, for example. Arguably, the recognition and nomination of subtribes runs the risk of introducing gratuitous taxonomic categories and even producing taxonomic inflation (Gippoliti & Groves, 2012); however, the potential of resolving “second-order relationships” (Pardiñas et al., 2020: 851) by far, outweighs these perceived risks.

**Genus *Anotomys*** Thomas, 1906a

**Type species:** *Anotomys leander* Thomas, 1906a

**Diagnosis:** as for the single genus contained, by monotypy.

**Included species:** *Anotomys leander* Thomas, 1906a.

**Remarks:** Morphological differences between Colombian and Ecuadorian specimens in the number of plantar pads and the presence/absence of the bacular cartilage deserve attention, although larger samples are needed to control individual and population variability. Transient pieces of cartilaginous tissues in young specimens of muroid rodents have been reported before, for example in *Peromyscus eremicus* (Spotorno, 1992) and plantar-pad polymorphisms have been previously reported for sigmodontines (*e.g*., Musser et al., 1998: 58). *Cytb* divergence between specimens from Colombia and Ecuador at 2.8% is well-within reported intraspecific divergence for sigmodontine rodents and other mammals (Allen et al., 2020; Schrago & Mello, 2020).

## Supplemental Information

10.7717/peerj.14319/supp-1 Supplemental Information 1 List of specimens analyzed in the study. List of specimens analyzed with geographic information and GenBank accession numbers for molecular data used in this study; codes in bold and red lettering were generated for this study, others from GenBank. Voucher acronyms refer to the following scientific collections: American Museum of Natural History, New York, United States (AMNH); Natural History Museum, London, UK (BMNH), Colección Nacional de Mamíferos, Ciudad de México, México (CNMA), Coleccion Teriológica de la Universidad de Antioquia, Colombia (CTUA), Field Museum of Natural History, Chicago, United States (FMNH); Instituto de Ciencias Naturales, Bogota, Colombia (ICN), Instituto de Pesquisas da Amazonia, Manaus, Brazil (INPA), Institut des Sciences de l´Evolution de Montpellier, France (ISEM), University of Kansas Natural History Museum, Lawrence, United States (KU); Louisiana State University Museum of Zoology, Baton Rouge, United States (LSUMZ), Museo Ecuatoriano de Ciencias, Quito (MECN), Museo de la Escuela Politécnica Nacional, Quito, Ecuador (MEPN), Museo de Historia Natural de la Universidad de Caldas, Colombia (MHN-UCa), Muséum d’histoire naturelle de la Ville de Genève, Switzerland (MHNG), Museo de Historia Natural La Salle, Venezuela (MHNLS), Museu Nacional, Universidade Federal do Rio de Janeiro, Brazil (MN), Museo de Zoologia, Universidad de Costa Rica, San José (MZUCR), Museu Paraense Emílio Goeldi, Belem, Para, Brazil (MPEG), Museum of Southwestern Biology, University of New Mexico, Albuquerque, United States (MSB); Museo de Historia Natural de El Salvador (MUHNES), Museo de Historia Natural de la Universidad Nacional de San Agustín de Arequipa, Peru (MUSA). Museo de Historia Natural, Universidad Nacional Mayor de San Marcos, Lima, Peru (MUSM); Museo de Zoología Alfonso L. Herrera, Ciudad de México, México (MZFC), Museo de Zoologia de la Universidad del Azuay, Ecuador (MZUA-MA), Museu de Zoologia da Universidade de Sao Paulo, Brazil (MZUSP), Swedish Royal Museum of Natural History, Stockholm (NMR), Museo de la Pontificia Universidad Catolica del Ecuador, Quito (QCAZ), Royal Ontario Museum, Canada (ROM), Universidade Federal Mato Grosso, Brazil (UFMT), Smithsonian National Museum of Natural History, United States (USNM), Coleccion de mamiferos de la Universidad del Valle, Colombia (UV), University of Michigan Museum of Zoology, United States (UMMZ). APG, JCR, JEC, and JMC correspond to field ID numbers for Alejandra Pinedo Guerrero (APG xx—specimen to be deposited at Museo de Zoologia, Universidad de Costa Rica), Juan Carlos Ramírez (JCR-09, specimen to be deposited at Museo de Ciencias Naturales “José Celestino Mutis”, Universidad de Pamplona, Norte Santander, Colombia); Javier E. Colmenares (JEC 213, specimen to be deposited at Colección de Mamíferos del Museo de Historia Natural de la Universidad Industrial de Santander), and Juan Martinez Ceron (JMC 309, specimen to be deposited at Museo de Ciencias Naturales de La Salle, Instituto Tecnológico Metropolitano, Medellin, Colombia).

10.7717/peerj.14319/supp-2 Supplemental Information 2 Ichthyomyine weights. Weights recorded for several ichthyomyine specimens, compiled from a variety of sources including specimen tags. * = see Table S1 in reference to Museum collection acronyms. ** A very young individual, reported as *Chibchanomys* sp. by Anderson (1997).

10.7717/peerj.14319/supp-3 Supplemental Information 3 Ichthyomyine molar to condylobasal length index. Sample size, mean and range values of the ratio of toothrow length to condylobasal length in ichthyomyine rodents. N = sample size. Original measurements taken by HZP or derived from several sources.

10.7717/peerj.14319/supp-4 Supplemental Information 4 Matrix of character state distributions for 57 qualitative characters among 23 putative ichthyomyine species. Character and character state descriptions in the main text. The table also also includes the character state distribution for specimen LSUMZ Mammals 14406.

10.7717/peerj.14319/supp-5 Supplemental Information 5 Time-calibrated phylogeny of Ichthyomyini. Bayesian chronogram of a majority-rule consensus obtained from combined autocorrelated clock analysis with FBD model. Node bars depict the uncertainty (95% HPDs) of age estimates.

## Additional Information and Declarations

### Field Study Permissions

The following information was supplied relating to field study approvals (*i.e*., approving body and any reference numbers): Collecting permits that allowed the study include, in alphabetic order: Bolivia (Colección Boliviana de Fauna, Resolución 14 Agosto 1992), Brazil (Instituto Brasileiro do Meio Ambiente e dos Recursos Naturais Renováveis (IBAMA), Pacajá:519/2014, Proc. 02001.001182/2014-65, Instituto Chico Mendes de Conservação da Biodiversidade (ICMBio), PN Pacaás Novos: 36753-3), Colombia (Autoridad Nacional de Licencias Ambientales del Ministerio de Ambiente y Desarrollo Sostenible de la República de Colombia: Resolución 1070 (28 August 2015), 01711 (30 Dic 2016), 0200 (13 Apr 2015), Costa Rica (R-SINAC-PNI-SE-002-2018); Ecuador (Ministerio del Ambiente, Resolución 002-IC-FAUFLO-DRFN-PMA, 010-IC-FAU/FLO-DRFN-P/MA, 012-IC-FAU-DNBAPVS/MA, MAE-DPAC-2014-0280, 2-12 IC-FAU-FLO-DPACMA), El Salvador (Resolución MARN-DEV-GVS-AIMA-087-2017), Perú (Dirección General de Flora y Fauna Silvestre [DGFFS], Servicio Nacional de Áreas Naturales Protegidas, Zona Reservada Sierra del Divisor and Santuario Histórico de Machupicchu: RD 503-2011-AG-DGFFS, RJ 003-2013-SERNANP-ZRSD, RJSHM-No. 054-2012-SERNANP-JEF.

### DNA Deposition

The following information was supplied regarding the deposition of DNA sequences: All sequences are available in the Supplemental File and at GenBank: MW148637–MW148737.

### Data Availability

The following information was supplied regarding data availability: The raw data are available in the Supplemental Files. The 3D Data is available at Morph D Base: Neusticomys vossi (MECN 4332), Koch, C. (2020): C_Koch_20201211-S-19.1. http://www.morphdbase.de/?C_Koch_20201211-S-19.1 Ichthyomys orientalis (MECN 4914), Koch, C. (2020): C_Koch_20201211-S-19.1. https://www.morphdbase.de/?C_Koch_20201215-S-20.1

### New Species Registration

The following information was supplied regarding the registration of a newly described species: Publication LSID: urn:lsid:zoobank.org:pub:92FE6565-5569-40EF-89B9-211DA7A16508 *Subtribe Anotomyina* nov. urn:lsid:zoobank.org:act:EB6195F3-3E5E-447D-BF60-D67658C6E874
