# Supplementary material for: Systematics and diversification of the Ichthyomyini (Cricetidae, Sigmodontinae) revisited: evidence from molecular, morphological, and combined approaches
Source: PeerJ. 2023 Jan 13;11:e14319. doi: 10.7717/peerj.14319 (PMC9841913; doi:10.7717/peerj.14319)
Supplement: Supplemental Information 1 — List of specimens analyzed with geographic information and GenBank accession numbers for molecular data used in this study; codes in bold and red lettering were generated for this study, others from GenBank. Voucher acronyms refer to the following scientific collections: American Museum of Natural History, New York, United States (AMNH); Natural History Museum, London, UK (BMNH), Colección Nacional de Mamíferos, Ciudad de México, México (CNMA), Coleccion Teriológica de la Universidad de Antioquia, Colombia (CTUA), Field Museum of Natural History, Chicago, United States (FMNH); Instituto de Ciencias Naturales, Bogota, Colombia (ICN), Instituto de Pesquisas da Amazonia, Manaus, Brazil (INPA), Institut des Sciences de l´Evolution de Montpellier, France (ISEM), University of Kansas Natural History Museum, Lawrence, United States (KU); Louisiana State University Museum of Zoology, Baton Rouge, United States (LSUMZ), Museo Ecuatoriano de Ciencias, Quito (MECN), Museo de la Escuela Politécnica Nacional, Quito, Ecuador (MEPN), Museo de Historia Natural de la Universidad de Caldas, Colombia (MHN-UCa), Muséum d’histoire naturelle de la Ville de Genève, Switzerland (MHNG), Museo de Historia Natural La Salle, Venezuela (MHNLS), Museu Nacional, Universidade Federal do Rio de Janeiro, Brazil (MN), Museo de Zoologia, Universidad de Costa Rica, San José (MZUCR), Museu Paraense Emílio Goeldi, Belem, Para, Brazil (MPEG), Museum of Southwestern Biology, University of New Mexico, Albuquerque, United States (MSB); Museo de Historia Natural de El Salvador (MUHNES), Museo de Historia Natural de la Universidad Nacional de San Agustín de Arequipa, Peru (MUSA). Museo de Historia Natural, Universidad Nacional Mayor de San Marcos, Lima, Peru (MUSM); Museo de Zoología Alfonso L. Herrera, Ciudad de México, México (MZFC), Museo de Zoologia de la Universidad del Azuay, Ecuador (MZUA-MA), Museu de Zoologia da Universidade de Sao Paulo, Brazil (MZUSP), Swedish Royal Museum of Natural History, Stockh [file peerj-11-14319-s001.pdf]

Taxa	Museum Specimen (Field Code)	Country	Locality	Lat	Lon	#loc	cytb	RBP3	GHR	RAG1
Rheomys mexicanus	MZFC 13624	Mexico	3.73 km (by road) NE of junction to Santiago Comaltepec, road 175 from Oaxaca to Tuxtepec	17.5507	-96.5036	1	**MW148678**	**MW148702**	**MW148735**	**MW148719**
Rheomys thomasi	AMNH 204261	Mexico	Ixhuatan: 1.8 miles west of Ixhuatan	17.2833	-93.0333	2				
Rheomys mexicanus	CNMA 42992	Mexico	Distrito de Ixtlan, Distrito de Santa Maria Yavesia	17.2250	-96.4278	3				
Rheomys mexicanus	AMNH 205313	Mexico	Juchitan, Unión Hidalgo	16.4750	-94.8297	4				
Rheomys mexicanus	AMNH 205316	Mexico	Juchitan, Unión Hidalgo	16.4750	-94.8297	4				
Rheomys mexicanus	AMNH 205318	Mexico	Juchitan, Unión Hidalgo	16.4750	-94.8297	4				
Rheomys mexicanus	AMNH 208257	Mexico	Juchitan, Unión Hidalgo	16.4750	-94.8297	4				
Rheomys mexicanus	AMNH 185015	Mexico	Juchitan, Unión Hidalgo, Chicapa River	16.4600	-94.8300	5				
Rheomys mexicanus	AMNH 185018	Mexico	Juchitan, Unión Hidalgo, Chicapa River	16.4600	-94.8300	5				
Rheomys mexicanus	AMNH 189291	Mexico	Juchitan, Unión Hidalgo, Chicapa River	16.4600	-94.8300	5				
Rheomys mexicanus	AMNH 205314	Mexico	Juchitan, Unión Hidalgo, Chicapa River	16.4600	-94.8300	5				
Rheomys mexicanus	AMNH 182139	Mexico	Miahuatlan, San José Lachiguiri	16.3778	-96.3353	6				
Rheomys mexicanus	AMNH 205321	Mexico	Miahuatlan, San José Lachiguiri	16.3778	-96.3353	6				
Rheomys thomasi	ROM 101294	El Salvador	Parque Nacional "El Imposible", sector San Francisco Menendez	13.8500	-90.0000	7	KR089036.2	KC953451	KC960491	KR089049
Rheomys thomasi	MUHNES 50-2168	El Salvador	Parque Nacional "El Imposible", sector San Benito, Rio Ahuachapio	13.8170	-89.9480	8	**MW148682**	**MW148705**	**MW148737**	**MW148721**
Rheomys thomasi	MZV 98799	El Salvador	Caraguatique	13.8000	-88.2300	9				
Rheomys thomasi	MZV 98805	El Salvador	Caraguatique	13.8000	-88.2300	9				
Rheomys raptor	MZUCR 4970	Costa Rica	Sector Pailas del Parque Nacional Rincón de la Vieja	10.7586	-85.3466	10	**MW148680**	**MW148703**	**MW148736**	**MW148720**
Ichthyomys pittieri	USNM 562980	Venezuela	Rancho Grande	10.6100	-67.6800	11				
Ichthyomys pittieri	MHNLS 8114	Venezuela	Farm "El Jaguar" 21 km NW of Aroa by road	10.5891	-68.9727	12				
Ichthyomys pittieri	MZUC 1000	Venezuela	Quebrada Palo Verde	10.4300	-68.8000	13				
Rheomys raptor	USNM 565826	Costa Rica	Guacimal River, Lawton Crossing below lechería	10.3000	-84.7000	14				
Rheomys sp.	KU 159017	Costa Rica	Reserva Biológica Bosque Nuboso de Monteverde; Quebrada Cuecha	10.3000	-84.7782	15	KF359512	AY163635		MF097935
Neusticomys venezuelae	AMNH 69908	Venezuela	Neverí	10.2500	-63.9160	16				
Ichthyomys pittieri	USNM 324987	Venezuela	Rancho Grande	10.1400	-67.3600	17				
Ichthyomys pittieri	USNM 562981	Venezuela	Palmichal, 23 Km N. Bejuma	10.0500	-68.6170	18				
Rheomys raptor	MZUCR 3205	Costa Rica	Quebrada Corralillo, Rancho Redondo hacia Llano Grande, Guadalupe.	9.9603	-83.9349	19	**MW148679**			
Rheomys raptor	MZUCR 5000 / TURC-425	Costa Rica	Río Blanco (Quebrada "Ciccaba"),Veragua Rainforest Park, Las Brisas, Liverpool	9.9301	-83.1909	20	**MW148681**	**MW148704**		
Rheomys underwoodi	APG s/n	Costa Rica	Alturas de Cotón	8.9379	-82.8228	21				
Rheomys raptor	USNM 520769	Panama	First Water or Upper Camp-1975	8.9333	-82.7000	22				
Rheomys underwoodi	USNM 516939	Panama	El Volcán, 17 Km NNW, Head of Rio Candela	8.9139	-82.7170	23				
Rheomys underwoodi	USNM 516940	Panama	El Volcán, 17 Km NNW, Head of Rio Candela	8.9139	-82.7170	23				
Rheomys raptor	USNM 396585	Panama	Cotito Hot Springs	8.8667	-82.7333	24				
Rheomys raptor	USNM 396586	Panama	Cotito Hot Springs	8.8667	-82.7333	24				
Ichthyomys tweedii	MNCR M2106	Costa Rica	Quebrada Wilson, Estación Biológica Las Cruces	8.7782	-82.9642	25				
Ichthyomys tweedii	USNM 461078	Panamá	Aguacate, Near Lidice	8.7500	-79.9666	26				
Ichthyomys tweedii	USNM 461094	Panamá	Aguacate, Near Lidice	8.7500	-79.9666	26				

Ichthyomys hydrobates hydrobates	AMNH 24355	Venezuela	Mérida	8.6100	-71.1333	27				
Ichthyomys hydrobates hydrobates	AMNH 24356	Venezuela	Mérida	8.6100	-71.1333	27				
Ichthyomys hydrobates hydrobates	USNM 123323	Venezuela	Mérida	8.6100	-71.1333	27				
Ichthyomys hydrobates hydrobates	USNM 151288	Venezuela	Mérida	8.6100	-71.1333	27				
Ichthyomys hydrobates hydrobates	USNM 172941	Venezuela	Mérida	8.6100	-71.1333	27				
Ichthyomys hydrobates hydrobates	AMNH 24351	Venezuela	Libertador	8.6000	-71.1333	28				
Ichthyomys hydrobates hydrobates	AMNH 24354	Venezuela	Libertador	8.6000	-71.1333	28				
Ichthyomys hydrobates hydrobates	AMNH 24357	Venezuela	Libertador	8.6000	-71.1333	28				
Rheomys raptor	USNM 179029	Panama	Mount Pirri, Rio Limon, Near Head Of	7.8630	-77.8500	29				
Chibchanomys trichotis	USNM 442606	Venezuela	Buena Vista, 41 km SW of San Cristóbal	7.4500	-72.4300	30				
Neusticomys mussoi	JCR 09	Colombia	Municipio de Chitagá, Vereda San Carlos	7.1559	-72.4786	31	**MW148657**			
Neusticomys mussoi	ICN 21295	Colombia	Vereda Rosa Blanca	7.1017	-73.0675	32				
Ichthyomys hydrobates hydrobates	CTUA 644	Colombia	Municipio de Toledo	7.0550	-75.6744	33				
Chibchanomys trichotis	JEC 213	Colombia	Farm "El Rasgon"	7.0426	-72.9806	34	**MW148642**	**MW148687**		
Ichthyomys hydrobates hydrobates	CTUA 163	Colombia	Municipio de Belmira	6.6133	-75.6703	35				
Neusticomys monticolus	FMNH 71221	Colombia	Urrao, Santa Barbara	6.4167	-76.2500	36				
Ichthyomys hydrobates hydrobates	JMC 309	Colombia	Jardín Botánico del Pacífico, Playa Mecana, Bahia Solano, Quebrada Resaquita	6.2736	-77.3733	37	**MW148643**			
Neusticomys sp.	CTUA 4274 (DAG 148)	Colombia	Municipio de Caldas, Corregimiento La Clara, Refugio de vida Silvestre Alto de San Miguel	6.0185	-75.5962	38	**MW148658**			
Neusticomys sp.	ICN 16526	Colombia	Andes, Vereda La Soledad, Finca La Reina	5.6003	-75.9339	39				
Ichthyomys hydrobates soderstromi	MHN-UCa 1439 (DCM 399)	Colombia	Quebrada Bolloliso, Municipio de Manzanares	5.5908	-74.9457	40	**MW148645**	**MW148688**		
Ichthyomys hydrobates hydrobates	BMNH 23.11.13.9	Colombia	Paime	5.3667	-74.1667	41				
Ichthyomys hydrobates hydrobates	CTUA 2576 (DCM 166)	Colombia	Municipio de Manzanares	5.3114	-75.1161	42	**MW148644**			
Neusticomys oyapocki	AMNH 267597	Guyane	Sinnamary, Paracou	5.2800	-52.9160	43				
Neusticomys venezuelae	AMNH 257344	Venezuela	San Ignacio de Yuruaní	5.0300	-61.1300	44				
Neusticomys sp.	ICN 12118	Colombia	Pereira, Vereda La Suiza, Qda. La Hacienda	4.7500	-75.5500	45				
Chibchanomys trichotis	FMNH 71226	Colombia	San Cristobal	4.5667	-74.0833	46				
Neusticomys sp.	CTUA 2619 (DCM 260)	Colombia	Municipio de Cajamarca, Finca Alejandria	4.5510	-75.5008	47	**MW148659**			
Neusticomys sp.	CTUA 2621 (DCM 330)	Colombia	Municipio de Cajamarca, Quebrada La Colosa, Sitio La Vara	4.4503	-75.4806	48	**MW148660**	**MW148694**		**MW148713**
Chibchanomys trichotis	ICN 10152	Colombia	Pasca, Vereda Chisaca, Lake Rebosadero	4.2800	-74.2200	49				
Neusticomys oyapocki	ISEM V-1647	French Guiana	Nouragues field station	4.0833	-52.6833	50				
Anotomys leander	CTUA 2556 (DCM 258)	Colombia	Municipio de Cajamarca	4.0500	-75.5000	51	**MW148637**	**MW148683**	**MW148722**	**MW148706**
Neusticomys oyapocki	ISEM V-3509 (T-7127)	Guyane	Municipality of Regina: Savane-Roche Annabelle	4.0000	-52.2500	52	**MW148662**		**MW148731**	**MW148715**
Neusticomys venezuelae	USNM 406123	Venezuela	Cerro Duida	3.6200	-65.6800	53				
Neusticomys vossi	UV 13739	Colombia	Municipio de Florida, La Herrera páramo	3.3230	-76.0714	54	**MW148677**	**MW148701**		
Neusticomys monticolus	UV 11249	Colombia	La Herrera	3.3200	-76.0600	55				
Ichthyomys hydrobates hydrobates	USNM 294985	Colombia	Munchiquito mountain	2.5300	-76.9500	56				
Ichthyomys hydrobates hydrobates	ICN 10029	Colombia	El Tambo	2.4600	-76.8100	57				
Neusticomys vossi	UV 13704	Colombia	Parque Nacional Puracé, Termales de San Juan	2.3436	-76.3081	58	**MW148676**	**MW148700**		

Neusticomys oyapocki	MHNG 1978.042 (T-5760)	Guyane	Municipality of Camopi: Trois-Sauts	2.2500	-52.8700	59	**MW148661**	**MW148695**	**MW148730**	**MW148714**
Neusticomys oyapocki	MNHN 1977.775	French Guiana	Trois Sauts	2.1600	-53.1000	60				
Neusticomys oyapocki	MPEG 34251	Brazil	Fazenda Itapoa, Distrito Amapá	2.0667	-50.9333	61				
Neusticomys monticolus	AMNH 66848	Ecuador	Guaranda; Sinchig	1.5330	-78.9830	62				
Ichthyomys stolzmanni orientalis	BMNH 24.4.18.8	Ecuador	Río Napo	0.8333	-77.8160	63				
Neusticomys monticolus	QCAZ 13066 (TEL 2470)	Ecuador	Reserva Integral Otonga	0.7188	-77.9803	64	**MW148656**			
Neusticomys vossi	MECN 5027 / CNP 6379	Ecuador	Reserva Ecológica El Ángel	0.7188	-77.9803	64				
Neusticomys vossi	QCAZ 12531 (QKM 51165)	Ecuador	Reserva Ecológica El Ángel	0.7188	-77.9803	64				
Neusticomys vossi	QCAZ 12532 (QKM 51171)	Ecuador	Reserva Ecológica El Ángel	0.7188	-77.9803	64	**MW148673**			
Neusticomys vossi	QCAZ 9888	Ecuador	Jesús del Gran Poder, Loma Guagua	0.4974	-77.7743	65				
Neusticomys vossi	AMNH 244608	Ecuador	Quijos, 1.6 kilometers E of Papallacta	0.3667	-78.1330	66				
Neusticomys vossi	AMNH 244609	Ecuador	Quijos, 1.6 kilometers E of Papallacta	0.3667	-78.1330	66				
Ichthyomys hydrobates hydrobates	AMNH 244610	Ecuador	Santo Domingo, Old Santo Domingo Trail	0.2830	-78.7160	67				
Neusticomys monticolus	AMNH 64625	Ecuador	San Ignacio	0.2000	-78.5500	68				
Neusticomys monticolus	AMNH 64626	Ecuador	San Ignacio	0.2000	-78.5500	68				
Neusticomys vossi	QCAZ 11672 (QKM 50492)	Ecuador	Zuleta, Hda. San Pedro	0.1944	-78.0595	69	**MW148672**			
Neusticomys vossi	QCAZ 11667 (QKM 50455)	Ecuador	Zuleta, Hda. San Pedro	0.1944	-78.0595	69	**MW148668**			
Neusticomys vossi	QCAZ 11668 (QKM 50456)	Ecuador	Zuleta, Hda. San Pedro	0.1944	-78.0595	69	**MW148669**			
Neusticomys vossi	QCAZ 11669 (QKM 50459)	Ecuador	Zuleta, Hda. San Pedro	0.1933	-78.0520	70	**MW148670**			
Neusticomys vossi	QCAZ 11670 (QKM 50460)	Ecuador	Zuleta, Hda. San Pedro	0.1931	-78.0528	71				
Neusticomys vossi	QCAZ 11671 (QKM 50473)	Ecuador	Zuleta, Hda. San Pedro	0.1931	-78.0528	71	**MW148671**			
Neusticomys vossi	QCAZ 12530 (QKM 51156)	Ecuador	Zuleta, Hda. San Pedro	0.1931	-78.0528	71				
Anotomys leander	MEPN 12580	Ecuador	Parque Nacional Cayambe Coca, Sector San Marcos	0.1255	-77.9680	72				
Neusticomys vossi	QCAZ 15898 (NTL 50)	Ecuador	Puerto Quito	0.1175	-79.2472	73	**MW148675**			
Ichthyomys tweedii	AMNH 71382	Ecuador	San Miguel de los Bancos, Mindo, Pechahal	0.1100	-78.8300	74				
Ichthyomys tweedii	AMNH 71383	Ecuador	San Miguel de los Bancos, Mindo, Pechahal	0.1100	-78.8300	74				
Ichthyomys tweedii	AMNH 71384	Ecuador	San Miguel de los Bancos, Mindo, Pechahal	0.1100	-78.8300	74				
Ichthyomys tweedii	AMNH 71385	Ecuador	San Miguel de los Bancos, Mindo, Pechahal	0.1100	-78.8300	74				
Ichthyomys tweedii	AMNH 71387	Ecuador	San Miguel de los Bancos, Mindo, Pechahal	0.1100	-78.8300	74				
Ichthyomys tweedii	AMNH 71389	Ecuador	San Miguel de los Bancos, Mindo, Pechahal	0.1100	-78.8300	74				
Ichthyomys tweedii	BMNH 34.9.10.172	Ecuador	Mindo, Below rio Blano	0.0563	-78.7776	75				
Anotomys leander	FMNH 53367	Ecuador	Chinchin Cocha	0.0500	-78.1167	76				
Neusticomys monticolus	QCAZ 6446 (TEL 1541)	Ecuador	Río Tandayapa	0.0060	-78.6765	77	KF359515	KR105606		
Neusticomys monticolus	QCAZ 6531 / ACUNCH 900 (TEL 1531)	Ecuador	Río Tandayapa	0.0060	-78.6765	77	KF359516	KR105605	**MW148729**	**MW148712**
Neusticomys oyapocki	INPA 5154	Brazil	Monte Dourado	0.0000	-52.6000	78				
Anotomys leander	AMNH 66201	Ecuador	Quito Mount Pichincha	-0.1876	-78.5854	79				
Anotomys leander	AMNH 66202	Ecuador	Quito Mount Pichincha	-0.1876	-78.5854	79				
Ichthyomys hydrobates soderstromi	AMNH 39593	Ecuador	Quito, Guapulo	-0.2000	-78.4830	80				
Ichthyomys hydrobates soderstromi	AMNH 39594	Ecuador	Quito, Guapulo	-0.2000	-78.4830	80				
Ichthyomys hydrobates soderstromi	AMNH 46729	Ecuador	Quito, Guapulo	-0.2000	-78.4830	80				

Ichthyomys hydrobates soderstromi	AMNH 46730	Ecuador	Quito, Guapulo	-0.2000	-78.4830	80				
Ichthyomys hydrobates soderstromi	AMNH 46731	Ecuador	Quito, Guapulo	-0.2000	-78.4830	80				
Ichthyomys hydrobates soderstromi	AMNH 46732	Ecuador	Quito, Guapulo	-0.2000	-78.4830	80				
Ichthyomys hydrobates soderstromi	AMNH 46733	Ecuador	Quito, Guapulo	-0.2000	-78.4830	80				
Ichthyomys hydrobates soderstromi	AMNH 64624	Ecuador	Quito, Guapulo	-0.2000	-78.4830	80				
Ichthyomys hydrobates soderstromi	NMR 586089	Ecuador	Quito, Guapulo	-0.2000	-78.4833	81				
Ichthyomys hydrobates soderstromi	NMR 586090	Ecuador	Quito, Guapulo	-0.2000	-78.4833	81				
Ichthyomys hydrobates soderstromi	NMR 586091	Ecuador	Quito, Guapulo	-0.2000	-78.4833	81				
Ichthyomys hydrobates soderstromi	NMR 586092	Ecuador	Quito, Guapulo	-0.2000	-78.4833	81				
Ichthyomys hydrobates soderstromi	NMR 586093	Ecuador	Quito, Guapulo	-0.2000	-78.4833	81				
Neusticomys monticolus	AMNH 64627	Ecuador	Río San Rafael	-0.3065	-78.4482	82				
Neusticomys vossi	QCAZ 4145 (CBT 4368)	Ecuador	Papallacta	-0.3342	-78.1433	83				
Anotomys leander	AMNH 244605	Ecuador	Quijos, 6.9 km of Papallacta by road	-0.3660	-78.1330	84				
Anotomys leander	AMNH 244606	Ecuador	Quijos, 6.9 km of Papallacta by road	-0.3660	-78.1330	84				
Anotomys leander	AMNH 244607	Ecuador	Quijos, 6.9 km of Papallacta by road	-0.3660	-78.1330	84				
Anotomys leander	QCAZ 6256 / UMMZ 155602	Ecuador	6.2 km of Papallacta by road	-0.3755	-78.1455	85	**MW148638**	**MW148684**		
Neusticomys vossi	QCAZ 6257 / AMNH 155606	Ecuador	Papallacta	-0.3755	-78.1455	85				
Neusticomys monticolus	AMNH 64628	Ecuador	Pita River canyon	-0.4000	-78.4000	86				
Neusticomys monticolus	AMNH 64629	Ecuador	Pita River canyon	-0.4000	-78.4000	86				
Neusticomys monticolus	AMNH 64630	Ecuador	Pita River canyon	-0.4000	-78.4000	86				
Neusticomys monticolus	QCAZ 15033	Ecuador	Reserva La Otonga, San Francisco de Las Pampas	-0.4189	-79.0039	87	**MW148674**	**MW148699**	**MW148734**	**MW148718**
Neusticomys monticolus	QCAZ 8688	Ecuador	Reserva La Otonga, San Francisco de Las Pampas	-0.4189	-79.0039	87				
Neusticomys monticolus	QCAZ 8691 (MP 52, TK 149025)	Ecuador	Reserva La Otonga, San Francisco de Las Pampas	-0.4189	-79.0039	87	KF359517			
Neusticomys monticolus	QCVZ 8690 (MP 61, TK 149034)	Ecuador	Reserva La Otonga, San Francisco de Las Pampas	-0.4189	-79.0039	87	KF359518			
Ichthyomys hydrobates soderstromi	QCAZ 818	Ecuador	Sigchos, San Francisco de las Pampas	-0.4200	-78.9600	88				
Neusticomys monticolus	AMNH 63376	Ecuador	Santa Rosa, Rio Pita	-0.4295	-78.4104	89				
Neusticomys monticolus	AMNH 64631	Ecuador	Santa Rosa, Rio Pita	-0.4295	-78.4104	89				
Neusticomys monticolus	AMNH 64632	Ecuador	Santa Rosa, Rio Pita	-0.4295	-78.4104	89				
Neusticomys vossi	QCAZ 7830 (TEL 1846)	Ecuador	Bermejo, 11 km SE of Baeza by road	-0.5164	-77.8670	90	KF359513	KR105608		
Neusticomys vossi	QCAZ 8956 (TEL 1937)	Ecuador	Volcán Sumaco	-0.5698	-77.5940	91	KF359514	KR105607		
Neusticomys oyapocki	INPA 5151	Brazil	Monte Dourado	-0.6000	-52.6500	92				
Neusticomys oyapocki	INPA 5141	Brazil	Monte Dourado	-0.6800	-52.8100	93				
Ichthyomys stolzmanni orientalis	NMR 586098	Ecuador	Río Jatunyacu	-1.0904	-77.9827	94				
Ichthyomys stolzmanni orientalis	MEPN 12672 /QCAZ 17512	Ecuador	Tungurahua, Baños Río Zuñac	-1.3700	-78.0000	95	**MW148647**			
Neusticomys peruviensis	QCAZ 10352 (TK 106896)	Ecuador	Comunidad Kurintza, Río Villano	-1.5060	-78.1500	96	**MW148666**			
Neusticomys monticolus	QCAZ 9866	Ecuador	Páramo de Culebrillas	-1.5345	-78.9057	97				
Ichthyomys hydrobates soderstromi	QCAZ 907	Ecuador	San Miguel, Bosque protector Cashca Totoras	-1.7100	-78.9600	98				
Ichthyomys hydrobates soderstomi	MECN 3960	Ecuador	Pallatanga, Los Santiagos	-2.0827	-78.9872	99				
Ichthyomys stolzmanni orientalis	MECN 4914	Ecuador	Río Jurumbuno, Nueva Alianza	-2.1100	-78.1200	100	**MW148646**			
Neusticomys vossi	MECN 4332	Ecuador	Morona, Parque Nacional Sangay, Sector San Balón	-2.1959	-78.4652	101	**MW148667**			

Chibchanomys orcesi	MZUA MA037 (TK 182289)	Ecuador	Estrellas Cocha	-2.7851	-79.2360	102				
Chibchanomys orcesi	BMNH 82.815	Ecuador	Las Cajas, Lake Luspa	-2.8000	-79.2800	103				
Chibchanomys orcesi	BMNH 82.816	Ecuador	Las Cajas, Lake Luspa	-2.8333	-79.5000	104				
Chibchanomys orcesi	BMNH 84.349	Ecuador	Las Cajas, Lake Luspa	-2.8333	-79.5000	104				
Chibchanomys orcesi	MEPN 12230 (TK182287)	Ecuador	Parque Nacional Las Cajas	-2.8588	-79.1736	105	**MW148641**			
Ichthyomys tweedii	MEPN 12229 / QCAZ 17444 (TK 182286)	Ecuador	Camilo Ponce Enríquez, Río Tenguel	-3.0358	-79.6960	106	**MW148652**			
Ichthyomys pinei	MECN 5613	Ecuador	Nabón, La Playa, Río León	-3.3228	-79.0622	107				
Ichthyomys pinei	MZUA MA 234	Ecuador	Nabón, La Playa, Río León	-3.3228	-79.0622	107				
Neusticomys ferreirai	MPEG 41844	Brazil	Senador José Porfírio, Xingu River	-3.5800	-51.9300	108				
Neusticomys ferreirai	MPEG 42457	Brazil	Senador Jose Porfirio	-3.5844	-51.9383	109	KX792192			
Neusticomys sp.	MECN (JBM 2500)	Ecuador	Tundayme, Mirador	-3.5912	-78.4291	110				
Ichthyomys tweedii	MECN 5772	Ecuador	Santa Rosa, Río Santa Rosa	-3.5965	-79.8490	111	**MW148651**	**MW148690**		
Neusticomys peruviensis	MUSM 45735	Peru	Distrito de San Juan Bautista, Llanchama	-3.8671	-73.3969	112	MF592572			
Neusticomys peruviensis	MUSM 44974	Peru	Distrito de San Juan Bautista, Llanchama	-3.8700	-73.3972	113	MF592571			
Neusticomys peruviensis	MUSM 44975	Peru	Distrito de San Juan Bautista, Llanchama	-3.8705	-73.3969	114	MF592570			
Ichthyomys stolzmanni orientalis	USNM 513625	Ecuador	4 km E Sabanilla	-3.9573	-79.0488	115				
Neusticomys ferreirai	MZUSP s/n (X1M27)	Brazil	Pacaja	-4.0350	-51.0790	116	**MW148655**	**MW148693**	**MW148728**	**MW148711**
Neusticomys ferreirai	MPEG 40560	Brazil	Floresta Nacional Tapirapé-Aquiri	-5.7700	-50.5500	117				
Neusticomys ferreirai	UFMT 1265	Brazil	Floresta Nacional Tapirapé-Aquiri	-5.7700	-50.5500	117				
Neusticomys ferreirai	MN 74004	Brazil	Floresta Nacional Tapirapé-Aquiri	-6.2700	-50.5800	118				
Neusticomys venezuelae	AMNH 142818	Guyana	Mazaruni Region: Kartabo Point	-6.3800	-58.6833	119				
Ichthyomys stolzmanni stolzmanni	AMNH 10109 / 11817	Peru	Cajabamba	-7.7541	-77.8559	120				
Neusticomys peruviensis	MUSA 12657	Peru	Coronel Portillo, Iparía, Río Shesha	-8.1971	-73.9461	121	**MW148664**	**MW148697**	**MW148732**	**MW148717**
Chibchanomys sp.	LSUMZ 14406	Peru	E slope Cordillera Carpish, Carretera Central	-9.7050	-76.1500	122				
Neusticomys ferreirai	MZUSP 32092	Brazil	Juruena	-10.2333	-58.4833	123				
Neusticomys ferreirai	MZUSP 32093	Brazil	Juruena	-10.2333	-58.4833	123	**MW148654**	**MW148692**	**MW148727**	**MW148710**
Neusticomys ferreirai	MZUSP s/n (MTR 25579)	Brazil	Parque Nacional Pacaás Novos	-10.7830	-63.6270	124	**MW148663**	**MW148696**		**MW148716**
Neusticomys peruviensis	MUSM 9214	Peru	Parque Nacional del Manu, Puesto de Vigilancia de Pakitza	-11.9500	-71.2800	125				
Neusticomys peruviensis	MUSA 19658	Peru	Paucartambo; Villa Carmen	-12.8870	-71.3933	126	**MW148665**	**MW148698**	**MW148733**	
Chibchanomys sp.	MUSA 13864	Peru	Urubamba, Aguas calientes, Wiñaywayna	-13.1858	-72.5428	127	**MW148639**	**MW148685**	**MW148723**	**MW148707**
Chibchanomys sp.	MUSA 18964	Peru	Urubamba, Aguas calientes, Wiñaywayna	-13.1910	-72.5368	128	**MW148640**	**MW148686**	**MW148724**	
Chibchanomys sp.	MUSA 18965	Peru	Urubamba, Aguas calientes, Wiñaywayna	-13.1910	-72.5368	128				
Ichthyomys stolzmanni stolzmanni	MUSA 16431	Peru	Jatumpampa	-13.3447	-74.4508	129				
Ichthyomys stolzmanni stolzmanni	MUSA 17792	Peru	Jatumpampa	-13.3447	-74.4508	129	**MW148648**	**MW148689**	**MW148725**	**MW148708**
Ichthyomys stolzmanni stolzmanni	MUSA 18919	Peru	Jatumpampa	-13.3447	-74.4508	129	**MW148649**			
Ichthyomys stolzmanni stolzmanni	MUSA 18920	Peru	Jatumpampa	-13.3447	-74.4508	129				
Ichthyomys stolzmanni stolzmanni	MUSA 18953	Peru	Urubamba, Aguas Calientes, Mandorpampa	-13.3447	-74.4508	129	**MW148650**			
Ichthyomys stolzmanni stolzmanni	MUSA 18954	Peru	Urubamba, Aguas Calientes, Mandorpampa	-13.3447	-74.4508	129				
Ichthyomys stolzmanni stolzmanni	MUSM 25807	Peru	Huamanga, Vinchos, Restaurant El Bagrecito	-13.6000	-74.7500	130		MF097744		
Ichthyomys stolzmanni stolzmanni	MUSM 25808	Peru	Piscigranja Arizona	-13.6000	-74.0500	131				

Ichthyomys stolzmanni stolzmanni	MUSM 25809	Peru	Piscigranja Arizona	-13.6000	-74.0500	131				
Neusticomys sp.	MSB 250000 (NK 29535)	Bolivia	0.5 km E Saynani, Rio Zongo	-16.1186	-68.0794	132	**MW148653**	**MW148691**	**MW148726**	**MW148709**
