# Supplementary material for: Systematics and diversification of the Ichthyomyini (Cricetidae, Sigmodontinae) revisited: evidence from molecular, morphological, and combined approaches
Source: PeerJ. 2023 Jan 13;11:e14319. doi: 10.7717/peerj.14319 (PMC9841913; doi:10.7717/peerj.14319)
Supplement: Supplemental Information 2 — Weights recorded for several ichthyomyine specimens, compiled from a variety of sources including specimen tags. * = see Table S1 in reference to Museum collection acronyms. ** A very young individual, reported as Chibchanomys sp. by Anderson (1997). [file peerj-11-14319-s002.docx]

| *Species* | Specimen* | Body mass (in g) |
| --- | --- | --- |
| *Anotomys leander* | UMMZ 126294 | 51 |
| *Anotomys leander* | UMMZ 126295 | 51 |
| *Anotomys leander* | UMMZ 126296 | 41 |
| *Anotomys leander* | UMMZ 155598 | 37 |
| *Anotomys leander* | UMMZ 155599 | 55 |
| *Anotomys leander* | UMMZ 155600 | 62 |
| *Anotomys leander* | UMMZ 155601 | 48 |
| *Anotomys leander* | UMMZ 155602 | 35 |
| *Chibchanomys n. sp.* | MSB 250000** | 18 |
| *Chibchanomys orcesi* | BMNH 82.817 | 35 |
| *Chibchanomys orcesi* | BMNH 84.349 | 37 |
| *Chibchanomys orcesi* | BMNH 82.816 | 41 |
| *Chibchanomys orcesi* | LSUMZ 14406 | 25 |
| *Chibchanomys trichotis* | UMMZ 156376 | 43 |
| *Chibchanomys trichotis* | UMMZ 156532 | 57 |
| *Ichthyomys hydrobates* | USNM 513625 | 50 |
| *Ichthyomys hydrobates* | UMMZ 156375 | 74 |
| *Ichthyomys orientalis* | DMMECN 4914 | 140 |
| *Ichthyomys pittieri* | USNM 324987 | 65 |
| *Ichthyomys pittieri* | MZUC 1000 | 70 |
| *Ichthyomys pittieri* | MHNLS 8114 | 114 |
| *Ichthyomys stolzmanni* | MUSA 18954 | 106 |
| *Ichthyomys stolzmanni* | MUSA 18919 | 102 |
| *Ichthyomys tweedii* | UMMZ 126300 | 96 |
| *Ichthyomys tweedii* | UMMZ 155782 | 99 |
| *Ichthyomys tweedii* | UMMZ 155783 | 40 |
| *Ichthyomys tweedii* | UMMZ 155784 | 42 |
| *Ichthyomys tweedii* | UMMZ 155785 | >100 |
| *Ichthyomys tweedii* | UMMZ 155786 | >100 |
| *Ichthyomys tweedii* | UMMZ 155787 | >100 |
| *Ichthyomys tweedii* | UMMZ 155788** | 35 |
| *Ichthyomys tweedii* | MNCR-M2106 | 125 |
| *Ichthyomys tweedii* | USNM 461078 | 123 |
| *Ichthyomys tweedii* | USNM 460684 | 127 |
| *Ichthyomys tweedii* | USNM 461094 | 155 |
| *Neusticomys ferreirai* | MN 74004 | 18.2 |
| *Neusticomys ferreirai* | MZUSP s/n (X1M27) | 24 |
| *Neusticomys ferreirai* | MZUSP 32093 | 25 |
| *Neusticomys ferreirai* | UFMT 1265 | 31 |
| *Neusticomys ferreirai* | MPEG 42457 | 32 |
| *Neusticomys ferreirai* | MZUSP 32092 | 34 |
| *Neusticomys ferreirai* | MPEG 40560 | 36 |
| *Neusticomys ferreirai* | MPEG 41844 | 37 |
| *Neusticomys monticolus* | UV 11249 | 24 |
| *Neusticomys monticolus* | UV 13704 | 28 |
| *Neusticomys monticolus* | UMMZ 126297 | 38 |
| *Neusticomys monticolus* | UMMZ 126298 | 32 |
| *Neusticomys monticolus* | UMMZ 126299 | 31 |
| *Neusticomys monticolus* | UMMZ 155604 | 36 |
| *Neusticomys monticolus* | UMMZ 155605 | 38 |
| *Neusticomys monticolus* | UMMZ 155606 | 24 |
| *Neusticomys monticolus* | UMMZ 155789 | 38 |
| *Neusticomys monticolus* | UMMZ 155790 | 38 |
| *Neusticomys monticolus* | UMMZ 155791 | 30 |
| *Neusticomys monticolus* | UMMZ 155792 | 11 |
| *Neusticomys monticolus* | UMMZ 155793 | 42 |
| *Neusticomys monticolus* | UMMZ 155794 | 50 |
| *Neusticomys oyapocki* | MPEG 34251 | 21 |
| *Neusticomys oyapocki* | INPA 5151 | 25 |
| *Neusticomys oyapocki* | ISEM V-1647 | 38 |
| *Neusticomys oyapocki* | AMNH 267597 | 43 |
| *Neusticomys oyapocki* | INPA 5154 | 44 |
| *Neusticomys oyapocki* | MNHN 1977.775 | 47 |
| *Neusticomys oyapocki* | INPA 5141 | 50 |
| *Neusticomys venezuelae* | AMNH 257345 | 58 |
| *Neusticomys venezuelae* | AMNH 257344 | 66 |
| *Rheomys hartmanni* | USNM 565826 | 18 |
| *Rheomys mexicanus* | OAXMA 2077 | 88 |
| *Rheomys raptor* | UMMZ 111985 | 50.5 |
| *Rheomys raptor* | UMMZ 111986 | 43.5 |
| *Rheomys raptor* | UMMZ 111987 | 38.7 |
| *Rheomys raptor* | UMMZ 112300 | 40.2 |
| *Rheomys raptor* | UMMZ 112301 | 44.6 |
| *Rheomys raptor* | KU 159017 | 30 |
| *Rheomys thomasi* | ROM 101294 | 29 |
| *Rheomys underwoodi* | UMMZ 115459 | 42.5 |
| *Rheomys underwoodi* | UMMZ 115460 | 72.2 |
