# Supplementary material for: Systematics and diversification of the Ichthyomyini (Cricetidae, Sigmodontinae) revisited: evidence from molecular, morphological, and combined approaches
Source: PeerJ. 2023 Jan 13;11:e14319. doi: 10.7717/peerj.14319 (PMC9841913; doi:10.7717/peerj.14319)
Supplement: Supplemental Information 3 — Sample size, mean and range values of the ratio of toothrow length to condylobasal length in ichthyomyine rodents. N = sample size. Original measurements taken by HZP or derived from several sources. [file peerj-11-14319-s003.docx]

| Species | N | Mean | Range |
| --- | --- | --- | --- |
| *N. oyapocki* | 4 | 0.11 | 0.10 – 0.12 |
| *I. pittieri* | 7 | 0.12 | 0.09 – 0.13 |
| *I. s. orientalis* | 3 | 0.13 | 0.12 – 0.14 |
| *I. stolzmanni* | 8 | 0.13 | 0.12 – 0.16 |
| *I. tweedii* | 9 | 0.13 | 0.14 – 0.14 |
| *N. ferreirai* | 4 | 0.14 | 0.13 – 0.15 |
| *Neusticomys* n. sp. | 2 | 0.14 | 0.14 – 0.15 |
| *N. mussoi* | 3 | 0.15 | 0.14 – 0.17 |
| *N. peruviensis* | 4 | 0.15 | 0.14 – 0.17 |
| *I. h. hydrobates* | 13 | 0.15 | 0.12 – 0.18 |
| *I. h. soderstromi* | 8 | 0.15 | 0.14 – 0.17 |
| *N. venezuelae* | 4 | 0.16 | 0.15 – 0.16 |
| *R. thomasi* | 9 | 0.16 | 0.15 – 0.19 |
| *“Chibchanomys”* n. sp. | 4 | 0.17 | 0.17 – 0.18 |
| *C. orcesi* | 4 | 0.17 | 0.17 – 0.18 |
| *C. trichotis* | 6 | 0.17 | 0.13 – 0.22 |
| *N. monticolus* | 13 | 0.17 | 0.16 – 0.18 |
| *N. vossi* | 5 | 0.17 | 0.17 – 0.18 |
| *R. mexicanus* | 11 | 0.18 | 0.16 – 0.20 |
| *R. raptor* | 4 | 0.18 | 0.17 – 0.20 |
| *A. leander* | 6 | 0.19 | 0.17 – 0.20 |
| *R. underwoodi* | 3 | 0.19 | 0.17 – 0.21 |
