# Supplementary figures and images for: Systematics and diversification of the Ichthyomyini (Cricetidae, Sigmodontinae) revisited: evidence from molecular, morphological, and combined approaches
Source: PeerJ. 2023 Jan 13;11:e14319. doi: 10.7717/peerj.14319 (PMC9841913; doi:10.7717/peerj.14319)

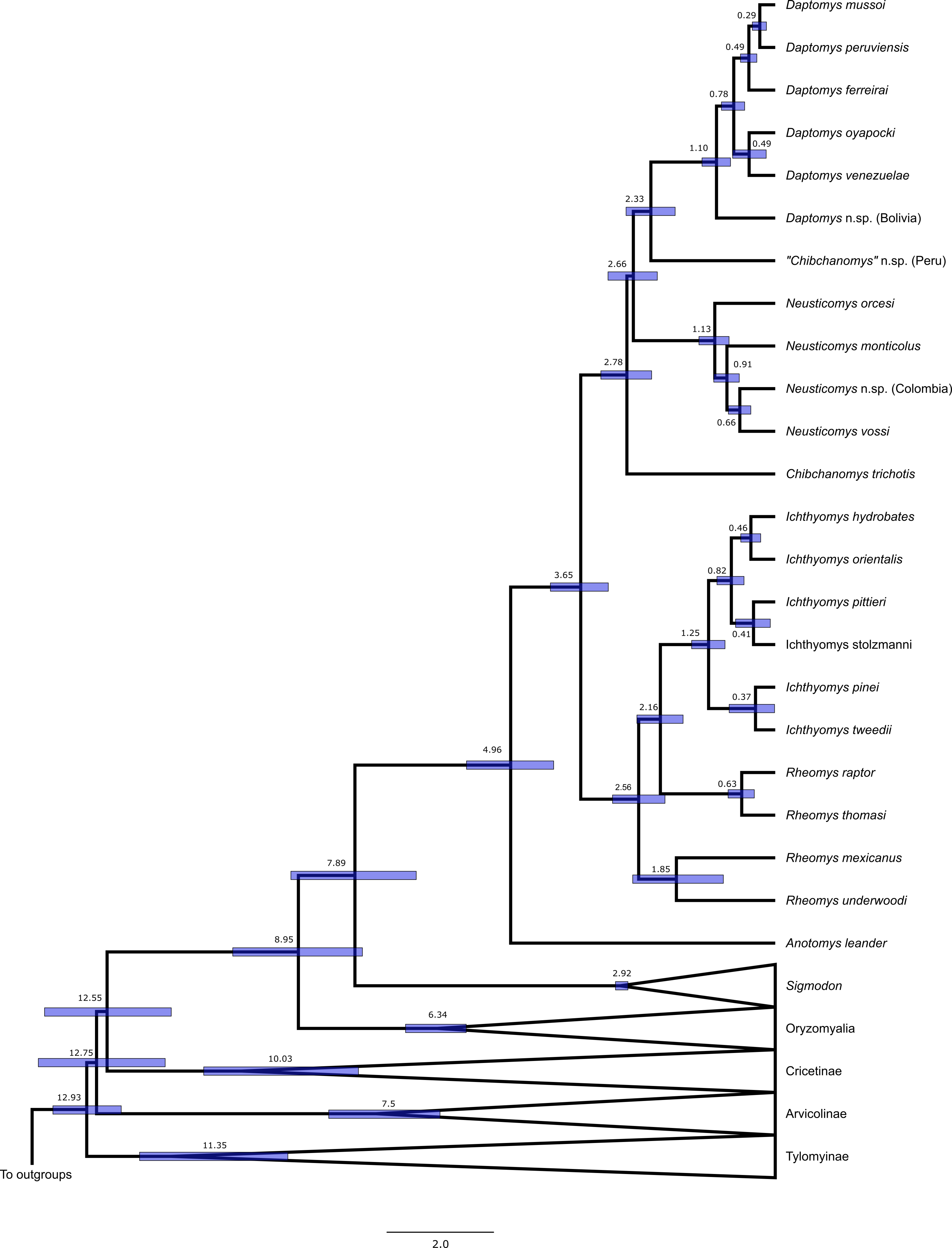

Supplement: Supplemental Information 5 — Bayesian chronogram of a majority-rule consensus obtained from combined autocorrelated clock analysis with FBD model. Node bars depict the uncertainty (95% HPDs) of age estimates. [file peerj-11-14319-s005.png]
